# USP18 promotes nasopharyngeal carcinoma radioresistance via TRIM29 oligomerization and ubiquitination

**DOI:** 10.1038/s41418-025-01615-3

**Published:** 2025-11-11

**Authors:** Jia-Yi Lin, Jun-Xiang Chen, Zi-Chen Qiu, Wei-Wei Zhang, Qing-Jie Li, Jun-Yan Li, Xu Jiang, Yu-Han Hu, Shi-Wei He, Shan Zhang, Ying-Qin Li, Na Liu, Jun Ma, Yin Zhao, Rui Guo

**Affiliations:** 1 Department of Radiation Oncology, Sun Yat-sen University Cancer Center; State Key Laboratory of Oncology in South China; Collaborative Innovation Center for Cancer Medicine; Guangdong Key Laboratory of Nasopharyngeal Carcinoma Diagnosis and Therapy, Guangzhou, 510060 China; 2 Department of Experimental Research, Sun Yat-sen University Cancer Center; State Key Laboratory of Oncology in South China; Collaborative Innovation Center for Cancer Medicine; Guangdong Key Laboratory of Nasopharyngeal Carcinoma Diagnosis and Therapy, Guangzhou, 510060 China

**Keywords:** Protein aggregation, Cancer

## Abstract

Radiotherapy, which induces DNA damage to control the progression of local tumors, is the mainstay therapy for nasopharyngeal carcinoma (NPC). However, almost a fifth of patients undergo recurrence. Evidence suggests that ubiquitination is crucial in DNA damage repair (DDR) signaling. In this study, we reveal that the ubiquitin specific peptidase 18 (USP18) is significantly overexpressed in resistant NPC tissues and correlates inversely with NPC cell radiosensitivity. Our findings indicate that USP18 interacts with tripartite motif containing 29 (TRIM29), facilitating its K27-linked ubiquitination independent of USP18’s catalytic activity. USP18 functions as a scaffold, recruiting the E3 ubiquitin ligase tripartite motif containing 21 (TRIM21), which directly ubiquitinates TRIM29 at Lys561. This process promotes TRIM29 oligomerization and nuclear translocation, which enhances DDR in NPC cells after radiotherapy. Clinically, high USP18 levels are associated with worse patient prognosis. Our findings underscore the critical role of USP18 in modulating DDR signaling and radiosensitivity in NPC, suggesting that targeting the USP18-TRIM21-TRIM29 axis may represent a novel strategy to enhance the efficacy of radiotherapy for patients with NPC.

## Introduction

Nasopharyngeal carcinoma, originating from the nasopharyngeal epithelium, is highly prevalent in East and Southeast Asia, particularly in southern China [1]. Radiotherapy is the primary treatment for NPC because this cancer is notably sensitive to radiation. However, radioresistance results in almost a fifth of patients undergoing recurrence [2]. The key cytotoxic mechanism of radiotherapy is DNA damage, which initials DDR signaling pathways involving multiple repair proteins [3]. Thus, targeting DDR signaling pathways has become an attractive strategy for overcoming tumor radioresistance [4].

Protein ubiquitination is widespread in eukaryotes, involving the E1 ubiquitin-activating enzyme, the E2 ubiquitin-conjugating enzyme, and the E3 ubiquitin ligase, while deubiquitinases (DUBs) can reversed the process by removing ubiquitin and ubiquitin-like (Ubl) signals from substrates [5, 6]. Ubiquitination and deubiquitylation exert critical functions in DDR signaling. For example, E3 ubiquitin ligase RNF8, RNF126 and RNF138 can ubiquitinate Ku80 to facilitate the non-homologous end-joining (NHEJ)-mediated DDR [7–9]. RNF126 also ubiquitinates MRE11 to increase its DNA exonuclease activity, thereby promoting DDR via the homologous recombination pathway [10]. RNF168 catalyzes the ubiquitination of H2A, facilitating the nuclear localization of 53BP1 and enhancing NHEJ-mediated DDR efficiency [11]. These studies illustrate the essential role of E3 ligases in DDR signaling; however, the role of DUBs in this process is poorly understood. Previously, we demonstrated that USP44 deubiquitinates and stabilizes TRIM25, facilitating Ku80 ubiquitination and degradation, and inhibiting irradiation-induced NHEJ-mediated DDR in NPC [12]. Additionally, Adan et al. has reported that deficiency of USP18 sensitized chronic myeloid leukemia cells to irradiation [13]. The function and mechanism of DUBs in DDR signaling and radioresistance remain largely unknown.

Herein, by mapping the radioresistance profile of NPC through proteome and transcriptome sequencing, we identified USP18, a ISG15-specific DUB with high expression in resistant NPC tissues, which correlated negatively with prognosis of NPC cell radiosensitivity. Furthermore, USP18 acts as a scaffold to recruit TRIM21, which directly ubiquitinates TRIM29 at Lys561. This process promotes TRIM29 oligomerization and nuclear translocation, thereby facilitating irradiation-induced DDR signaling. Clinically, higher USP18 expression correlates with poor prognosis in patients with NPC. This study uncovers the critical role of USP18 in DDR signaling and radiosensitivity regulation, suggesting that targeting the USP18-TRIM21-TRIM29 axis may represent a novel strategy to enhance radiotherapy efficacy in patients with NPC.

## Results

### USP18 is highly expressed in NPC and negatively correlates with radiosensitivity

**Fig. 1:**
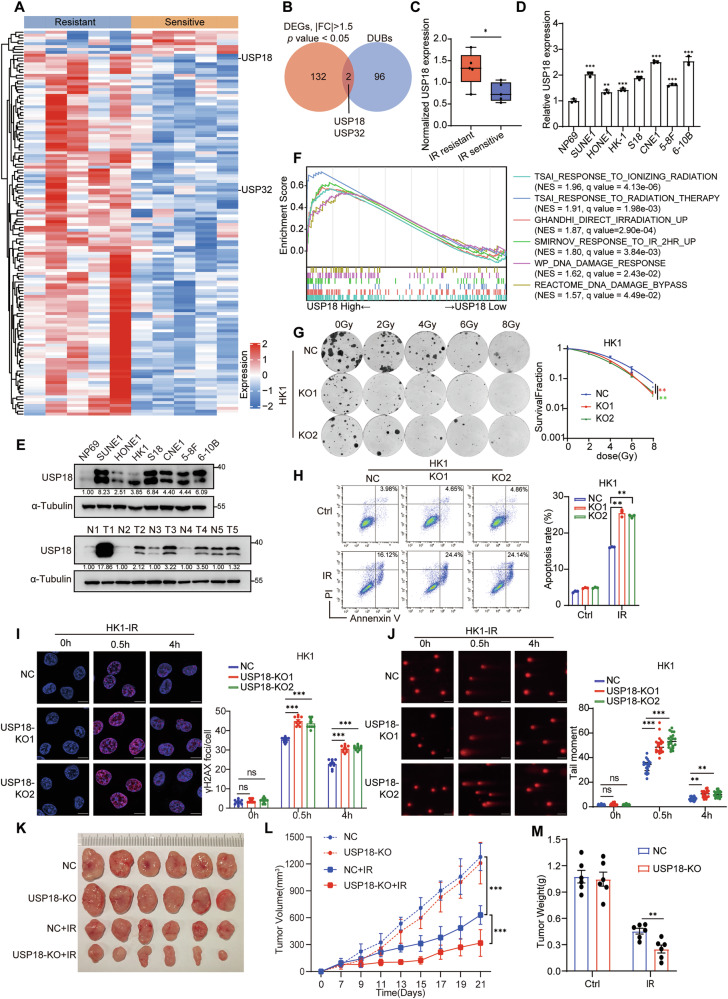
USP18 is highly expressed in NPC and impairs radiosensitivity. **A** Heatmap of differentially expressed proteins ( | fold change | > 1.5 and *p* < 0.05) identified by proteome sequencing in five paired NPC biopsy tissues from patients with (irradiation-resistant) or without (irradiation-sensitive) relapse after radiotherapy. **B** Venn diagram showing common proteins between differentially expressed proteins identified by proteome sequencing and DUBs from the iUUCD database. **C** Normalized protein expression level of USP18 in these five paired NPC tissues. **D** RT-qPCR analysis of relative USP18 mRNA expression in NP69 and various NPC cell lines (SUNE1, HONE1, HK-1, S18, 5-8 F, 6-10B and CNE1). **E** Western blot analysis of USP18 and α-Tubulin protein expression in NP69 and NPC cell lines, along with normal (N) and NPC (T) tissues. **F** GSEA of the GSE102349 dataset revealed enrichment of radiation response-related and DNA damage response pathways in the USP18 high-expression group. **G** Clonogenic assays and survival fraction curves of USP18-KO or NC HK1 cells after exposure to varying IR doses. **H** Flow cytometry analysis of apoptosis rates in USP18-KO or NC HK1 cells with or without 8 Gy IR exposure for 48 h. Representative images and quantitative analysis of γH2AX foci **I**, comet assay and tail moments **J** in USP18-KO or NC HK1 cells treated with 8 Gy IR for 0 h, 0.5 h and 4 h. Scale bars, 10 μm, *n* = 10 **I**. Scale bars, 100μm, n = 20 **J**. Macroscopic images **K**, average tumor volume **L**, and average weight **M** of excised tumors in each group (*n* = 6). Data are presented as mean ± SD in **D, G** to **J, L** and **M**; *p < 0.05, **p < 0.01, ***p < 0.001; *p* values were determined using two-way ANOVA; *n* = 3 independent experiments. The unprocessed images of the blots are shown in Fig. S11.

To map the radioresistance profile of NPC, we conducted proteome sequencing on five paired NPC biopsy tissues from patients with or without relapse following radiotherapy (Table S1). We identified 126 upregulated proteins and 8 downregulated proteins in the irradiation-resistant group *vs*. the irradiation-sensitive group **(**Fig. 1A**)**. Certain highly expressed proteins, for example, RPL22L1, SOX9, and CXCL13, promote therapy resistance, metastasis, and tumor growth in cancers [14–17] **(**Fig. S1A**)**. There were two DUBs among them, USP18 and USP32 **(**Fig. 1B, C). Across three public datasets (GSE53819 [18], GSE12452 [19] and GSE180272 [20]), USP18, but not USP32, was consistently highly expressed in NPC tissues **(**Fig. S1B-D**)**. Further examination of four paired NPC biopsy tissues from patients with or without relapse after radiotherapy in GSE102349 [21], indicated notable upregulation of USP18 (but not USP32) in the irradiation-resistant group **(**Fig. S1E**)**. Additional assessment showed that USP18 mRNA and protein levels were elevated in NPC cell lines compared with those in normal nasopharyngeal epithelial cells (NP69) **(**Fig. 1D, E**)**. Similarly, USP18 protein levels were increased in NPC tissues compared with those in normal nasopharyngeal tissues **(**Fig. 1E**)**. Moreover, *USP18* was highly expressed in other solid tumors **(**Fig. S1F**)**. Gene set enrichment analysis (GSEA) suggested that *USP18* expression correlated positively with pathways related to radiation therapy and DDR **(**Fig. 1F**)**. Additionally, USP18 mRNA and protein levels were significantly induced in a time-dependent manner following irradiation **(**Fig. S1G, H**)**. As irradiation activates IFN signaling [22], we found that USP18 expression was also induced by IFNβ treatment **(**Fig. S1I, J**)**. Collectively, these data demonstrate that high expression of in NPC correlates negatively with radiosensitivity.

### USP18 impairs the NPC cell radiosensitivity in vitro and in vivo

To confirm USP18’s function in NPC radiosensitivity, we generated *USP18*-knockout (KO) SUNE1 and HK1 cell lines using the clustered regularly interspaced short palindromic repeats (CRISPR)/CRISPR-associated protein 9 (CAS9) technique. Genomic sequencing and western blotting validated the efficiency of USP18-KO **(**Fig. S2A, B**)**. The clonogenic survival rate of USP18-KO cells was significantly lower than that of the control cells after irradiation **(**Fig. 1G, S2C**)**. Additionally, USP18 deficiency slightly enhanced cell apoptosis without irradiation treatment, but significantly increased apoptosis after irradiation compared with control cells **(**Fig. 1H, S2D**)**. The formation of γH2AX foci, a marker of DNA damage [23], significantly increased in the USP18-KO group following irradiation treatment **(**Fig. 1I, S2E**)**. Additionally, comet assays were performed to assess the level of DNA damage and the results showed a longer tail moment in the USP18-KO cells, indicating increased DNA damage **(**Fig. 1J, S2F**)**. Thus, USP18 deficiency impairs DDR signaling following radiation exposure.

Previous research highlighted USP18’s crucial role in regulating IFN-I signaling [24, 25]. We assessed whether blocking IFN-I signals affected USP18-mediated radiosensitivity. Results showed that USP18-KO significantly increased NPC cell apoptosis after irradiation, while inhibiting JAK1/2 (Ruxolitinib) or blocking IFNAR1 (Anifrolumab) did not alter apoptotic cell proportion **(**Fig. S3A**)**. Immunofluorescence and comet assays also revealed no significant differences in γH2AX foci or comet tail moment in USP18-KO cells, treated with or without the two agents **(**Fig. S3B, C**)**. Furthermore, we examined the expression of interferon-stimulated genes (ISGs) in NPC cells, including IFIT1, IFIT2, MX1, ISG15, OAS1, and CXCL10. The results showed that irradiation treatment induced the expression of ISGs, whereas USP18 deficiency synergized with irradiation to induce the expression of MX1 and OAS1 without affecting other genes **(**Fig. S3D**)**. Additionally, except for the notable upregulation of IFI27L2, USP18 and ISG20L2 in the irradiation-resistant group, the expression of common ISGs showed no significantly alteration in the proteomics data **(**Fig. S3E**)**. Collectively, these results indicate that USP18 primarily modulates the DNA damage repair pathway rather than IFN-I signaling in NPC cells upon irradiation treatment.

Next, we obtained an irradiation-resistant HONE1 cell line (HONE1-IR-R) for further study [26] and observed that USP18 was highly expressed compared to parental HONE1 cells **(**Fig. S4A**)**. *USP18* knockdown in HONE1-IR-R cells markedly inhibited the post-irradiation clonogenic ability and increased the proportion of apoptotic cells **(**Fig. S4B–D**)**. To evaluate the role of USP18 in radiosensitivity in vivo, we generated subcutaneous tumor xenograft models. Knockout of USP18 significantly reduced tumor growth after irradiation, with no significantly changes observed without irradiation **(**Fig. 1K–M**)**. Together, these results demonstrate that USP18 impairs the radiosensitivity of NPC cells in vitro and in vivo.

### USP18 interacts with TRIM29

**Fig. 2:**
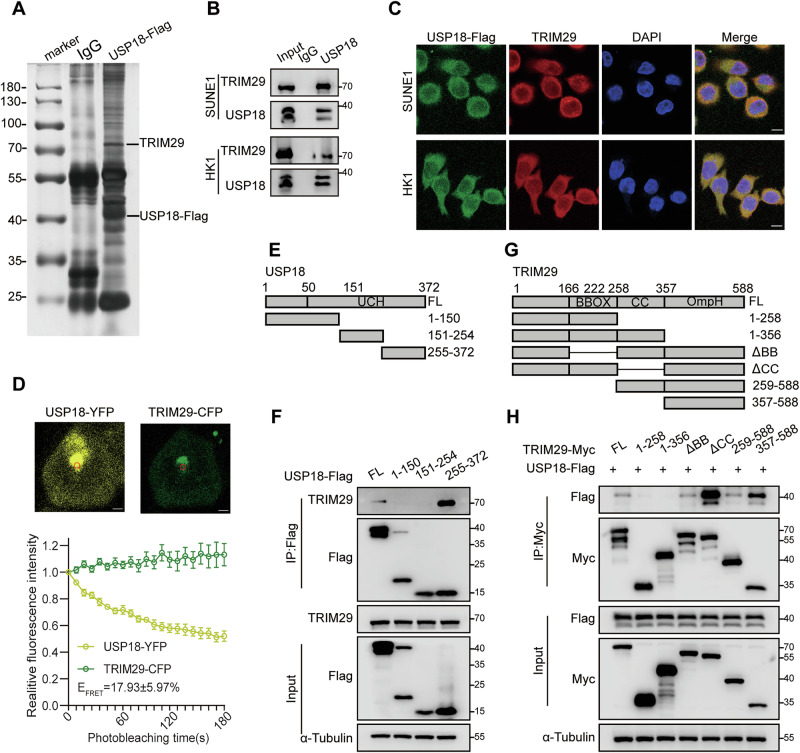
USP18 interacts with TRIM29. **A** Silver staining of SDS-PAGE gels displaying immunoprecipitated proteins from SUNE1 cells overexpressing USP18-Flag. The proteins of interest are indicated. **B** Co-IP with anti-USP18 antibody in NPC cells showing interaction between endogenous USP18 and TRIM29. **C** Immunofluorescence staining depicting the cellular localization of exogenous Flag-USP18 (green) and endogenous TRIM29 (red). Scale bars: 10 μm. **D** FRET assay in HEK293T cells transfected with USP18-YFP and TRIM29-CFP. Representative cells are shown, and the FRET analysis region is indicated. The decrease in USP18-YFP (acceptor) fluorescence and increase in TRIM29-CFP (donor) fluorescence during YFP photobleaching were analyzed in 10 cells, with FRET efficacy (E_FRET_) reported as the mean ± SEM. Scale bar: 2 μm. **E** Schematic representation of USP18-Flag truncation mutants. **F** Co-IP assay (anti-Flag) of USP18-Flag truncation mutants with endogenous TRIM29 in SUNE1 cells. **G** Schematic representation of TRIM29-Myc truncation mutants. **H** Co-IP assay (anti-Myc) between TRIM29-Myc truncations and USP18-Flag in HEK293T cells co-transfected with both. Results are representative of three independent experiments **B** and **C, F, H**. The unprocessed images of the blots are shown in Fig. S11.

To determine how USP18 regulates NPC cell radiosensitivity, we performed immunoprecipitation-mass spectrometry (IP-MS) analysis, which identified TRIM29 as a potential target of USP18 **(**Figs. 2A, S5A, Table S2**)**. TRIM29 promotes the assembly of DDR proteins on damaged chromatin, thus facilitating DDR [27, 28]. Endogenous immunoprecipitation assays verified the interaction between USP18 and TRIM29 in SUNE1 and HK1 cells **(**Fig. 2B**)**. Additionally, immunofluorescence staining revealed the cytoplasmic co-localization of USP18 and TRIM29 **(**Fig. 2C**)**. To further validate the USP18–TRIM29 interaction, we conducted the fluorescence resonance energy transfer (FRET) assay, which is independent of antibodies and allows non-radiative energy transfer from an excited fluorescent donor cyan fluorescent protein (CFP) to an acceptor fluorophore yellow fluorescent protein (YFP) in very close proximity [29, 30]. The results demonstrated that the fluorescence intensity of the donor TRIM29-CFP increased, while the acceptor USP18-YFP was photobleached, resulting in a FRET efficiency (E_FRET_) of 17.93 ± 5.97%, thereby confirming interactions between TRIM29-CFP and USP18-YFP proteins **(**Figs. 2D and S5B**)**. Furthermore, we constructed truncation mutants of USP18 [31] and conducted co-immunoprecipitation assays, which revealed that the USP18 ubiquitin carboxyl-terminal hydrolase (UCH) domain interacted with TRIM29 **(**Fig. 2E, F**)**. Similarly, co-immunoprecipitation with generated truncation mutants of TRIM29 [32] indicated that the C-terminal OmpH domain, but not the N-terminal, B-box (BB), or coiled-coil (CC) domains, interacted with USP18 **(**Fig. 2G, H**)**. Notably, deletion of the CC domain of TRIM29 significantly enhanced its interaction with USP18 (Fig. 2H). Thus, TRIM29 and USP18 interact via the UCH domain of USP18 and the OmpH domain of TRIM29.

### USP18 promotes K27-linked ubiquitination of TRIM29 independent of its catalytic activity

**Fig. 3:**
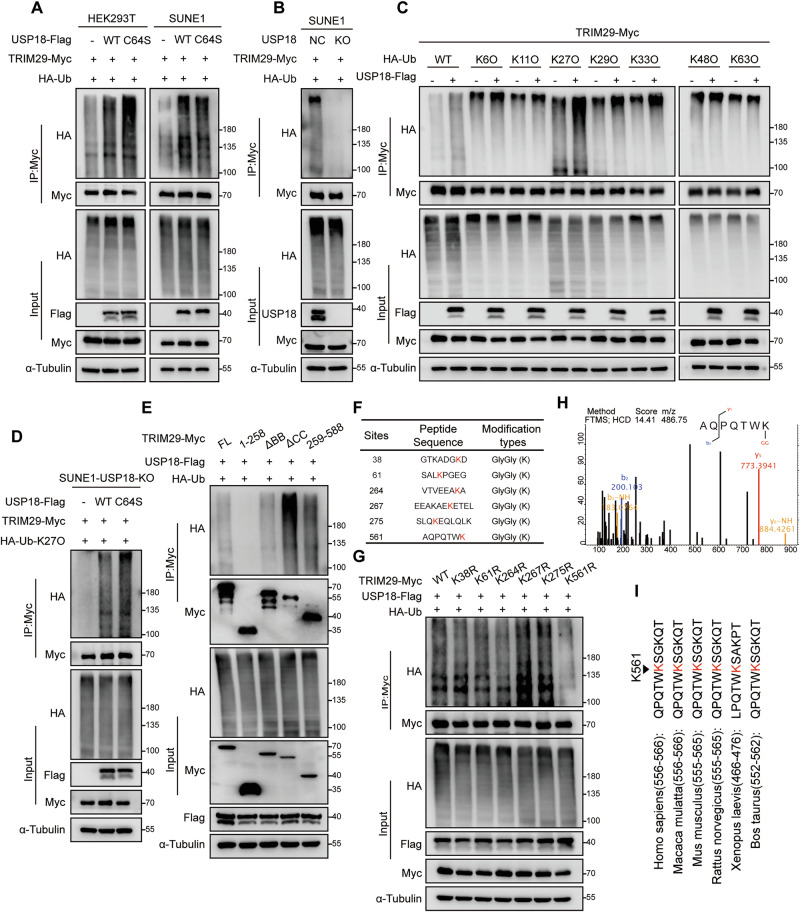
USP18 promotes K27-linked ubiquitination of TRIM29 independent of its catalytic activity. **A** Denatured-IP assay (anti-Myc) and western blotting (anti-Myc, HA, and α-Tubulin) in HEK293T and SUNE1 cells transfected with TRIM29-Myc, HA-Ub, and either vector, USP18-Flag WT, or C64S mutant. **B** Denatured-IP assay (anti-Myc) and western blot analysis (anti-Myc, HA, USP18, and α-Tubulin) in USP18-WT and USP18-KO SUNE1 cells transfected with TRIM29-Myc and HA-Ub. **C** Denatured-IP assay (anti-Myc) and western blot analysis (anti-Myc, HA, and α-Tubulin) in HEK293T cells transfected with TRIM29-Myc along with vector or USP18-Flag plus HA-Ub-WT or HA-Ub-KO mutants retaining only specific tyrosine sites of ubiquitin (K6O, K11O, K27O, K29O, K33O, K48O, and H63O). **D** K27O-linked ubiquitination level of TRIM29-Myc in USP18-KO SUNE1 cells supplemented with vector, USP18-Flag WT, or C64S mutant. **E** Ubiquitination levels of TRIM29-Myc truncations in HEK293T cells co-transfected with USP18-Flag and HA-Ub. **F** Mass spectrometry analysis of TRIM29 ubiquitination sites in SUNE1 cells co-transfected with TRIM29-Myc and HA-Ub-K27O. **G** Ubiquitination levels of TRIM29-Myc WT and KR mutants in HEK293T cells transfected with USP18-Flag and HA-Ub-K27O. **H** Mass spectrum of the K561 site on TRIM29. **I** Sequence alignment of the K561 site within TRIM29 orthologs across species. Results are representative of three independent experiments **A–E, G**. The unprocessed images of the blots are shown in Fig. S11.

To investigate whether USP18 regulates the ubiquitination of TRIM29, we constructed a catalytically inactive USP18 mutant (C64S) [31]. Overexpression of both wild-type USP18 (WT) and USP18 (C64S) significantly enhanced, rather than removed, the ubiquitin chain of TRIM29 **(**Fig. 3A**)**. In addition, reintroducing USP18 (WT) and USP18 (C64S) into USP18-KO NPC cells did not result in significant changes in the ISGylation of TRIM29 between the WT and C64S groups **(**Fig. S6A**)**. We further overexpressed USP18, ISG15, E1, E2 and E3 ligases [33] in HEK293T cells and the results consistently confirmed our findings **(**Fig. S6B**)**, suggesting that USP18 promotes ubiquitination but not deISGylation of TRIM29, independent of its catalytic activity. Conversely, USP18 knockout greatly decreased TRIM29’s ubiquitination level **(**Fig. 3B**)**. Exploration of the specific type of ubiquitin chain on TRIM29 influenced by USP18 demonstrated that USP18 primarily enhanced the level of K27O-linked ubiquitin chains on TRIM29 **(**Fig. 3C**)**. This enhancement also occurred after USP18 (C64S) overexpression **(**Fig. S6C**)**. To further validate USP18’s effect on TRIM29 ubiquitination, we performed rescue assays by reintroducing vector, WT, or C64S into USP18-KO cells: re-expressing USP18 WT or C64S significantly increased the K27O-linked ubiquitin chain level on TRIM29, which was reduced by USP18-KO **(**Fig. 3D**)**. Next, we analyzed various TRIM29 truncation mutants and discovered that the C terminus, especially with the CC domain depleted, had enriched ubiquitination upon USP18 overexpression **(**Fig. 3E**)**. IP-MS analysis identified six lysine residues as potential ubiquitination sites on TRIM29 **(**Fig. 3F**)**. Subsequent co-immunoprecipitation assays using Lys/Arg (K/R) substitution mutants of TRIM29 revealed that the K561R mutant significantly resisted USP18-mediated ubiquitination **(**Fig. 3G, H**)**, indicating that K561 in the C-terminus is the principal site of USP18-mediated ubiquitination. Notably, K561 is conserved across TRIM29 orthologs in various species **(**Fig. 3I**)**, and Akimov et al. have identified K561 as a potential ubiquitination site [34]. Collectively, these results indicate that USP18 promotes K27-linked ubiquitination at K561 of TRIM29, independent of its catalytic activity.

### USP18 recruits TRIM21 to increase K27-linked ubiquitination of TRIM29

**Fig. 4:**
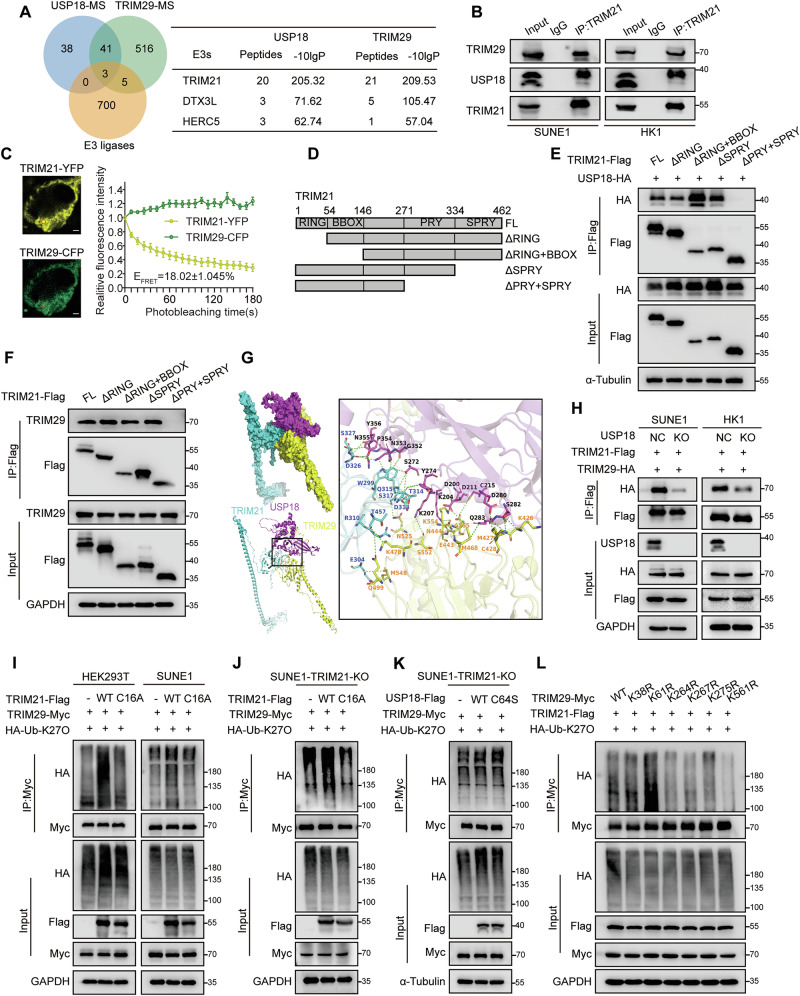
USP18 recruits TRIM21 to increase K27-linked ubiquitin chains on TRIM29. **A** Venn diagram showing common genes from mass spectrometry (MS) analysis of immunoprecipitated proteins from USP18-Flag and TRIM29-Myc in SUNE1 cells, along with E3 ligases from the iUUCD database. It indicates the number of TRIM21, DTX3L, and HERC5 peptides identified. **B** Co-immunoprecipitation (Co-IP) using anti-TRIM21 antibody in SUNE1 and HK1 cells demonstrated interactions among endogenous TRIM21, USP18, and TRIM29. **C** FRET assay in HEK293T cells transfected with TRIM21-YFP and TRIM29-CFP, showing representative cell images and the region used for FRET analysis. FRET efficacy (E_FRET_) is reported as mean ± SEM (*n* = 10). Scale bar: 2 μm. **D** Schematic representation of TRIM21-Flag truncation mutants. **E** Co-IP (anti-Flag) and western blot analysis (anti-Flag, HA, and α-Tubulin) in SUNE1 cells transfected with TRIM21-Flag truncation mutants and USP18-HA. **F** Co-IP (anti-Flag) and western blot analysis (anti-Flag, TRIM29, and GAPDH) in SUNE1 cells transfected with TRIM21-Flag truncation mutants. **G** Molecular docking of USP18, TRIM21, and TRIM29 as a ternary complex, with predicted amino acid linkages shown as dotted lines. **H** Co-IP (anti-Flag) and western blot analysis (anti-Flag, HA, USP18, and GAPDH) in WT or USP18-KO SUNE1 and HK1 cells co-transfected with TRIM21-Flag and TRIM29-HA. **I** Denatured-IP assay (anti-Myc) and western blotting analysis (anti-Myc, HA, Flag, and GAPDH) in HEK293T and SUNE1 cells transfected with TRIM29-Myc and HA-Ub-K27O plus vector, TRIM21-Flag WT, or C16A mutant. **J, K** Denatured-IP assay (anti-Myc) and western blotting analysis (anti-Myc, HA, Flag, and GAPDH) in TRIM21-KO SUNE1 cells transfected with TRIM29-Myc and HA-Ub-K27O plus vector or TRIM21-Flag WT or C16A mutant **J** or USP18-Flag WT or C64S mutant **K**. **L** Denatured-IP assay (anti-Myc) and western blotting analysis (anti-Myc, HA, Flag, and GAPDH) in HEK293T cells transfected with TRIM21-Flag and HA-Ub-K27O plus TRIM29-Myc WT or KR mutants. Data are representative of three independent experiments **B, E** and **F, H** to **L**. The unprocessed images of the blots are shown in Fig. S11.

USP18 itself does not exhibit E3 ubiquitin ligase activity; however, it acts as a scaffold to recruit other enzymes [31, 35]. To investigate whether USP18 similarly acts as a scaffold for TRIM29, we performed an IP-MS analysis of TRIM29 interaction partners in SUNE1 cells **(**Table S3**)**. We cross-referenced the IP-MS results with the findings for USP18 and known E3 ubiquitin ligases from the iUUCD database, which identified three E3 ligases, with TRIM21 being the most prevalent **(**Fig. 4A**)**. Endogenous co-immunoprecipitation assays confirmed interactions among TRIM21, USP18 and TRIM29 **(**Fig. 4B**)**. FRET assays further verified that TRIM21 interacted with both USP18 and TRIM29 **(**Figs. 4C, S7A**)**. A previous study identified two USP18 isoforms: the full length USP18-GCG36 mutant and the shorter N-terminal truncated isoform, USP18-sf [36]. The results from immunofluorescence assays showed that USP18-WT and USP18-GCG36 mutant were primarily located in the cytoplasm, with minimal presence in the nucleus, while USP18-sf was found in both cytoplasm and nucleus **(**Fig. S7B-C, green), consistent with previous study [36]. Additionally, USP18 colocalized with TRIM29 or TRIM21 mainly in the cytoplasm, instead of nucleus. Co-immunoprecipitation assays with TRIM21 truncation mutants showed that the TRIM21 PRY domain was required for its interaction with USP18 and TRIM29 **(**Fig. 4D–F**)**. Additionally, the C terminal regions of USP18 and TRIM29 were found to interact with TRIM21 **(**Fig. S7D, E**)**. Moreover, the ubiquitination of TRIM29, particularly with CC domain depletion, was enriched upon TRIM21 overexpression **(**Fig. S7F**)**. Structural simulation and molecular docking analyses revealed the specific amino acid interactions among TRIM21, USP18, and TRIM29, consistent with the truncation experiments **(**Fig. 4G, Table S4**)**. Notably, the TRIM21–TRIM29 interaction was substantially weaker in USP18-KO cells compared with that in USP18-WT cells, suggesting that USP18 served as a scaffold in this complex **(**Fig. 4H**)**.

To determine if TRIM21 regulates TRIM29 ubiquitination, we created an enzymatically inactive TRIM21 mutant (TRIM21-C16A) and performed co-immunoprecipitation [29]. The results showed that TRIM21-WT, but not TRIM21-C16A, promoted K27O-linked ubiquitination of TRIM29 **(**Fig. 4I**)**, which was corroborated by rescue assays in TRIM21-KO cells **(**Fig. 4J**)**. To confirm USP18’s role as a scaffold for TRIM21-mediated ubiquitination of TRIM29, we re-expressed vector, USP18-WT, or USP18-C64S in TRIM21-KO cells and performed co-immunoprecipitation assays. This revealed that USP-18-mediated TRIM29 ubiquitination relies on TRIM21, because it was unaffected in the absence of TRIM21 **(**Fig. 4K**)**. Furthermore, TRIM21, like USP18, facilitated K27O-linked ubiquitination of TRIM29 at K561 **(**Fig. 4L**)**. Together, these results demonstrate that USP18 recruits TRIM21 to increase the K27-linked ubiquitin chain on TRIM29.

### TRIM29 ubiquitination promotes its oligomerization and nuclear translocation

**Fig. 5:**
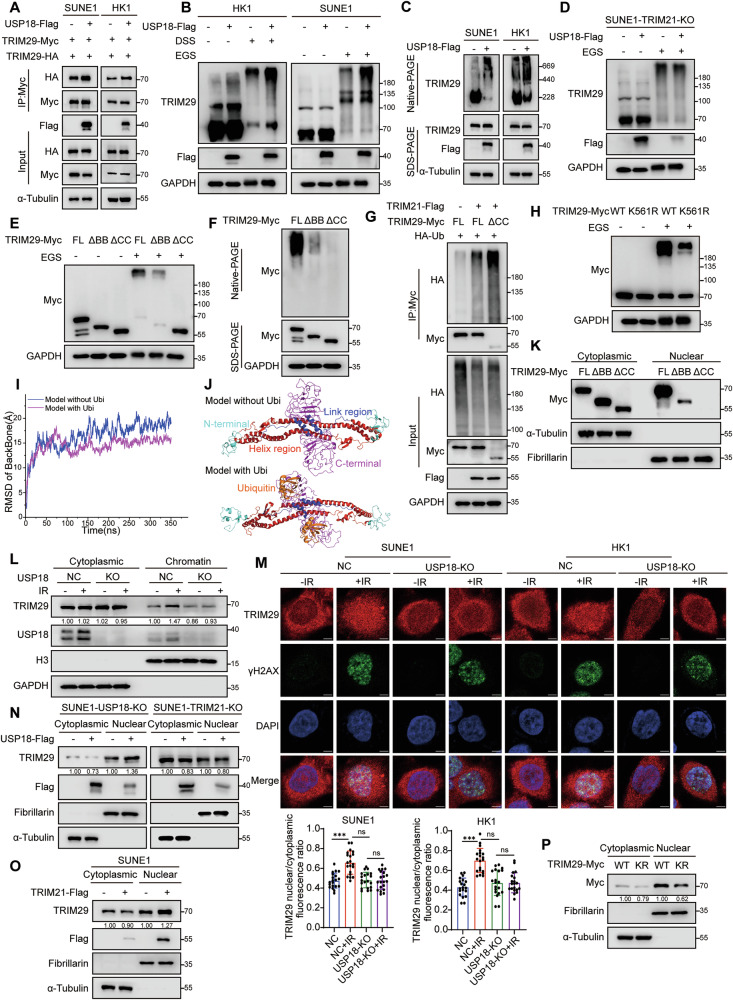
Ubiquitination of TRIM29 promotes oligomerization and facilitates nuclear translocation. **A** Co-IP assay (anti-Myc) and western blotting analysis (anti-Flag, HA, Myc, and α-Tubulin) in SUNE1 and HK1 cells transfected with TRIM29-HA and TRIM29-Myc, with or without USP18-Flag. **B** Western blot analysis (anti-Flag, TRIM29, and GAPDH) of lysates from SUNE1 and HK1 cells with or without USP18 overexpression, treated with DSS (5 mM) or EGS (5 mM) for 15 min. **C** Native-PAGE (top) and SDS-PAGE (bottom) immunoblotting (anti-Flag, TRIM29, and α-Tubulin) analysis of TRIM29 aggregation in SUNE1 and HK1 cells transfected with vector or USP18-Flag. **D** Western blot analysis (anti-Flag, TRIM29, and GAPDH) of lysates from TRIM21-KO SUNE1 cells transfected with vector or USP18-Flag, treated with EGS (5 mM) for 15 min. **E** Western blot analysis (anti-Myc and GAPDH) of lysates from HEK293T cells transfected with TRIM29-Myc and its truncation mutants ΔBB and ΔCC, treated with EGS. **F** Native-PAGE and SDS-PAGE analysis (anti-Myc and GAPDH) of HEK293T cells transfected with TRIM29-Myc and its truncation mutants ΔBB and ΔCC. **G** Denatured-IP and western blot analysis demonstrated the ubiquitination levels of TRIM29-Myc and the ΔCC mutant in HEK293T cells expressing TRIM21-Flag. **H** EGS treatment of lysates from SUNE1 cells transfected with TRIM29-Myc WT or K561R mutant followed by immunoblot analysis (anti-Myc and GAPDH). **I** RMSD variation of the TRIM29 dimer model with or without ubiquitin during a 350 ns MD simulation stabilized after approximately 200 ns. **J** Stable structures of the TRIM29 dimer model obtained from MD simulations, with the N-terminal, C-terminal, Helix, and Link regions colored cyan, red, blue, and magenta, respectively. **K** Cytoplasmic and nuclear extraction from HEK293T cells transfected with TRIM29-WT, ΔBB, and ΔCC mutants, analyzed via immunoblot (anti-Myc, Fibrillarin, and α-Tubulin). **L** Chromatin and cytoplasmic extraction from WT and USP18-KO HK1 cells treated with or without IR (8 Gy) for 24 h, analyzed by immunoblot (anti-TRIM29, USP18, H3, and GAPDH). **M** Immunofluorescence staining showing TRIM29 distribution in SUNE1 or HK1 WT and USP18-KO cells treated with or without IR (8 Gy) for 24 h. The nuclear/cytoplasmic fluorescence ratio was calculated using ImageJ software. Scale bars: 5 μm, *n* = 20. **N** Immunoblot analysis of cytoplasmic and nuclear proteins from SUNE1 or TRIM21-KO SUNE1 cells transfected with or without USP18-Flag (anti-TRIM29, Flag, Fibrillarin, and α-Tubulin). **O** Immunoblot analysis of cytoplasmic and nuclear proteins from SUNE1 cells transfected with TRIM21-Flag WT. **P** Immunoblot analysis of cytoplasmic and nuclear proteins from SUNE1 cells transfected with TRIM29-Myc WT or K561R. Data are representative of three independent experiments **A–H, K, L, N–P**. Results are presented as mean ± SD; ***p < 0.001, ns: not significant; *p* values were determined using two-way ANOVA. The unprocessed images of the blots are shown in Fig. S11.

Next, we investigated the impact of ubiquitination on TRIM29 stability. Overexpression or knockout of USP18 did not alter TRIM29 protein levels **(**Fig. S8A, B**)**. It was suggested that TRIM29 might oligomerize through its zinc finger motifs and a leucine zipper motif [37]. We hypothesized that TRIM29 ubiquitination might affect its oligomerization. TRIM29 oligomerization was markedly enhanced by USP18 or TRIM21 overexpression, as shown by increased monomer interactions and higher-order oligomer formation in NPC cells **(**Figs. 5A, B, S8C, D**)**. Furthermore, native-PAGE assays confirmed TRIM29 aggregation upon USP18 or TRIM21 overexpression **(**Figs. 5C, S8E**)**, which was abolished in TRIM21-depleted cells **(**Fig. 5D**)**, indicating that TRIM21 is required for USP18-mediated TRIM29 oligomerization.

Next, we investigated the domain responsible for TRIM29 oligomerization. Depletion experiments demonstrated that the CC domain of TRIM29 was crucial for oligomerization, with the BB domain also playing a significant role, as confirmed by both standard and native-PAGE assays **(**Fig. 5E, F**)**. These findings are consistent with previous reports that the BB and CC domains within TRIM family proteins are mainly responsible for their dimerization and oligomerization [38, 39]. Remarkably, the ubiquitination levels of TRIM29-ΔCC were higher when induced by USP18 or TRIM21 compared with those of TRIM29-WT **(**Fig. 5G, S8F**)**. The enhanced interaction between USP18 and TRIM29-ΔCC mutant underscored this finding **(**Fig. 2H**)**, and aligned with previous data on the ubiquitination of TRIM29-ΔCC **(**Figs. 3E, S7F**)**. Moreover, TRIM29-K561R formed fewer dimers and oligomers than TRIM29-WT **(**Figs. 5H, S8G, H**)**.

We conducted molecular dynamic (MD) simulations to explore whether TRIM29, alone or with ubiquitin linked to its K561 site, could form a dimer. The TRIM29 dimer was modeled based on the crystalline structure of the homologous TRIM72 dimer [38, 40] **(**Fig. S9A**)**. Analysis showed the fluctuation of root mean square error (RMSD) in the model with ubiquitin were smaller **(**Fig. 5I**)**, suggesting that ubiquitin stabilizes the TRIM29 dimer. The B-Factor was consistently lower in the ubiquitin model, particularly affecting the helix region **(**Fig. S9B**)**. Secondary structure analysis indicated that ubiquitin facilitates the formation of more helix secondary structures in this region **(**Fig. S9C**)**. Post-MD simulations confirmed stable structures, with the helix and C-terminal regions closer in the ubiquitin model, enhancing binding strength between TRIM29 monomers **(**Fig. 5J**)**. Moreover, the MM-PBSA calculated binding free energies indicate that the enhanced binding primarily stems from these regions **(**Fig. S9D, Table S5**)**. Collectively, these findings highlight the critical role of K561 ubiquitination in TRIM29 dimerization and oligomerization.

Since TRIM29 has a reported role in facilitating DDR, we investigated whether ubiquitination and oligomerization influenced its nucleocytoplasmic translocation. Both TRIM29-WT and TRIM29-ΔBB showed nuclear translocation, whereas the TRIM29-ΔCC did not, underscoring the necessity of the CC domain for this process **(**Fig. 5K**)**. We further explored whether TRIM29 relocates from the cytoplasm to damaged chromatin upon irradiation and the impact of USP18 on this translocation. Chromatin/cytoplasmic separation experiments demonstrated a significant increase in TRIM29’s chromatin presence post-irradiation, which was diminished in USP18-KO cells **(**Fig. 5L**)**. Immunofluorescence assays confirmed TRIM29 nuclear accumulation, showing colocalization with γH2AX following irradiation, indicating that USP18 mediated TRIM29’s nuclear translocation in response to irradiation **(**Fig. 5M**)**. Moreover, in USP18-KO cells re-expressing USP18, irradiation increased nuclear TRIM29 levels, which effect was absent in TRIM21-KO cells **(**Fig. 5N**)**. Furthermore, TRIM21 overexpression significantly raised nuclear TRIM29 levels upon irradiation, implying that TRIM21 was required for irradiation-induced TRIM29 nuclear translocation **(**Fig. 5O**)**. Additionally, TRIM29-K561R showed reduced nuclear translocation compared with TRIM29-WT **(**Fig. 5P**)**. In conclusion, we demonstrate that USP18 recruits TRIM21 to ubiquitinate TRIM29, facilitating its oligomerization and nuclear translocation.

### USP18 impairs radiosensitivity by facilitating DDR through TRIM29

**Fig. 6:**
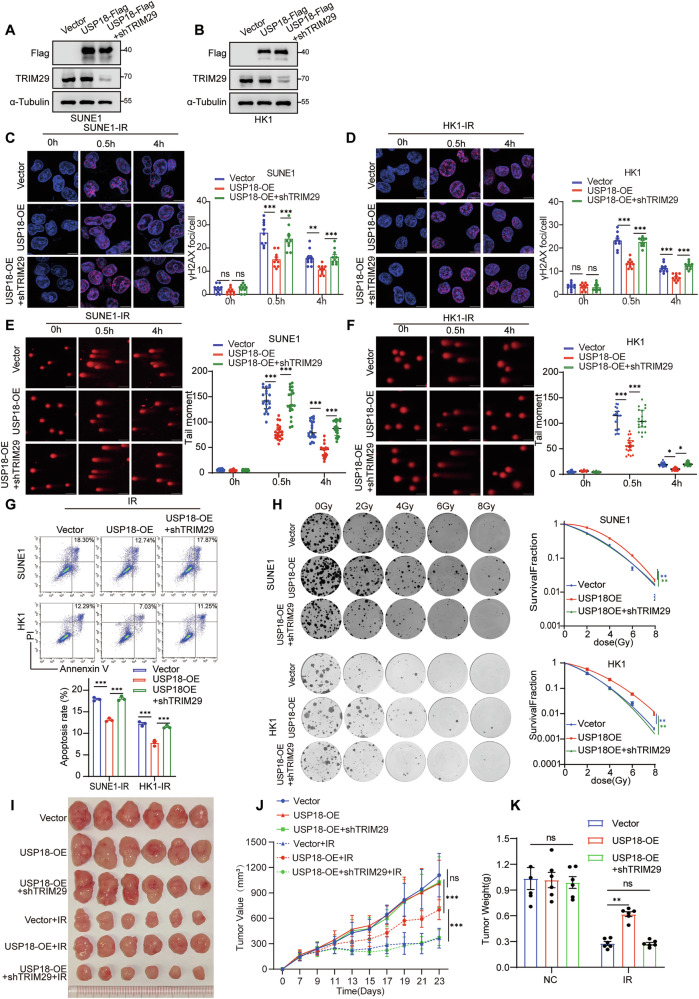
USP18 impairs radiosensitivity by facilitating DNA damage repair through TRIM29. **A, B** Western blot analysis (anti-Flag, TRIM29, and α-Tubulin) in SUNE1 and HK1 cells stably expressing vector, USP18-Flag, or USP18-Flag plus shTRIM29. **C, D** Representative images and quantitative analysis of γH2AX foci in SUNE1 and HK1 cells treated with 8 Gy IR for 0, 0.5, or 4 h. Scale bars: 10 μm, *n* = 10. **E, F** Representative comet images and quantitative analysis of tail moments for DNA damage in SUNE1 or HK1 cells treated with 8 Gy IR for 0, 0.5, or 4 h. Scale bars: 100 μm, *n* = 20. **G** Flow cytometry analysis of apoptosis rates in SUNE1 or HK1 cells exposed to 8 Gy IR. **H** Clonogenic assays of SUNE1 and HK1 cells following exposure to indicated IR doses. Macroscopic images **I**, average tumor volume **J**, and average weight **K** of excised tumors in each group (*n* = 6). Data are presented as mean ± SD; *p < 0.05, **p < 0.01, ***p < 0.001, with *p* values determined using two-way ANOVA; *n* = 3 independent experiments. The unprocessed images of the blots are shown in Fig. S11.

TRIM29 has been reported to promote DDR [28, 41]; therefore, we constructed short hairpin RNAs (shRNAs) targeting *TRIM29* and performed flow cytometry, which showed an increased fraction of apoptotic cells upon *TRIM29* knockdown **(**Fig. S10A, B**)**, thus verifying the protective role of TRIM29 in resisting irradiation. Next, we established stable cell lines overexpressing USP18, with or without *TRIM29* knockdown, to validate that USP18-mediated TRIM29 regulation weakens radiosensitivity **(**Fig. 6A, B**)**. USP18 overexpression reduced the number of γH2AX foci, whereas *TRIM29* knockdown reversed this reduction **(**Fig. 6C, D**)**. Additionally, experiments in TRIM21-KO cells did not show regulation by USP18 and TRIM29 **(**Fig. S10C–E**)**, suggesting that USP18 mediates DDR through TRIM29 in a TRIM21-dependent manner. Subsequent comet assays showed that USP18 overexpression resulted in a shorter tail moment, indicating reduced DNA damage, which returned to baseline upon *TRIM29* knockdown **(**Fig. 6E, F**)**. Furthermore, USP18 decreased apoptosis and enhanced survival in a clonogenic survival assay following irradiation, which were reversed by *TRIM29* knockdown **(**Fig. 6G, H**)**. Notably, USP18 overexpression also promoted the growth of subcutaneous tumor xenograft after irradiation, which was inhibited by TRIM29 knockdown **(**Fig. 6I–K**)**. These results underscored the positive role of the USP18-TRIM29 axis in DDR, which vanished in TRIM21-KO cells. Together, these findings illustrate that USP18 impairs radiosensitivity in NPC cells through TRIM21-mediated regulation of TRIM29.

### Elevated USP18 levels are associated with worse prognosis in patients with NPC

**Fig. 7:**
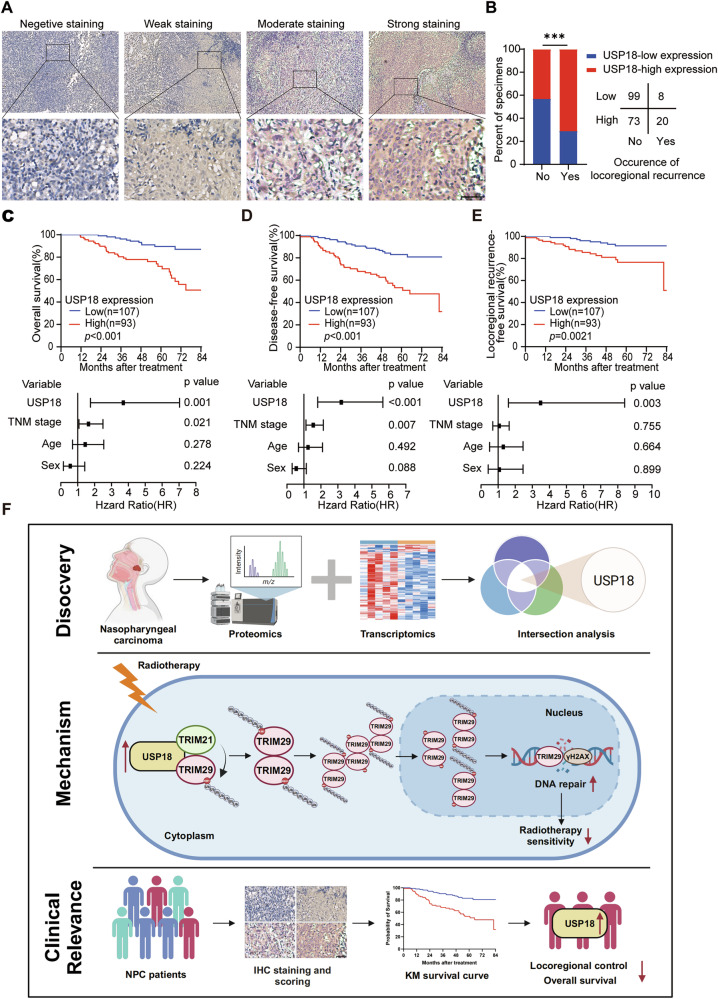
Elevated levels of USP18 are associated with worse prognosis for NPC patients. **A** Representative image of IHC staining showing USP18 protein expression in 200 NPC tissues. Scale bars: 50 μm. **B** Correlation of locoregional recurrence status with USP18 expression levels detected by IHC; *p* value determined using the two-tailed χ² test. Kaplan-Meier analysis (top) and forest plots of multivariate Cox regression analyses (bottom) for overall survival **C**, disease-free survival **D**, and locoregional recurrence-free survival **E** based on USP18 expression levels; *p* values from Kaplan-Meier analysis were calculated using the log-rank test. **F** Proposed working model of the USP18-TRIM21-TRIM29 axis in regulating NPC radiosensitivity and the clinical relevance of USP18 in NPC patients.

To explore the clinical relevance of USP18 in patients with NPC, we performed immunohistochemical staining on 200 NPC tissue samples, categorizing them by USP18 staining intensity **(**Fig. 7A**)**. Correlating with clinical data **(**Table S6**)**, we divided patients into high or low USP18 expression groups and found that high USP18 levels correlated significantly with locoregional recurrence **(**Fig. 7B**)**. Kaplan–Meier analysis revealed worse overall survival, disease-free survival, and locoregional recurrence-free survival in the USP18-high group. Multivariate Cox regression analysis identified USP18 as an independent risk factor for poor NPC prognosis **(**Fig. 7C–E**;** Table S7**)**. Thus, high USP18 expression predicts poor prognosis and increased recurrence risk in NPC.

## Discussion

Although evidence has revealed the vital function of E3s in DDR signaling and radioresistance, the exact role of DUBs in these processes remains unclear. Our study identified the a ISG15-specific deubiquitinase USP18 through proteomic and transcriptomic sequencing, verifying its negative regulation of radiosensitivity by impairing DDR signaling in NPC. Specifically, USP18 acts as a scaffold to recruit TRIM21, which ubiquitinates TRIM29 at K561, thereby facilitating its oligomerization and nuclear translocation, and leading to enhanced DDR signaling and radioresistance. Importantly, elevated USP18 levels in patients with NPC are associated with worse prognosis. Our findings provide broader insights into the role of DUBs and E3s in DDR signaling regulation and identify novel targets to potentially reverse NPC radioresistance.

USP18 is a ISG15-specific deubiquitinase, which removes ISG15 rather than ubiquitin from multiple substrates [42]. For example, USP18 deISGylates BECN1, enhancing its K63-linked ubiquitination and promoting autophagy [33]. Similarly, USP18 deISGylates ADAR, sustaining its ability to combine and reduce dsDNA, which enhances the sensitivity of chronic myeloid leukemia cells to irradiation [13]. Additionally, USP18 functions as a scaffold, independent of its catalytic activity, to facilitate signal transduction. For example, USP18 binds to the intracellular domain of IFNAR2, inhibiting its recruitment and activation of JAK1, thus negatively regulating interferon signaling [43]. Nuclear USP18 inhibits the binding of the STAT1-STAT2 complex to chromatin, impairing the expression of interferon-stimulated genes [44]. USP18 also acts as a scaffold to recruit USP20, allowing it to deubiquitinate and stabilize STING [31]. Likewise, USP18 recruits MIB2 to ubiquitinate and degrade GSDMD [45]. These diverse functions of USP18 highlight the multifaceted roles of DUBs in biological process beyond their catalytic activity. In this study, USP18, as a scaffold, recruits TRIM21 ubiquitinating TRIM29 and facilitating DDR signaling and radioresisitance in NPC. This emphasizes the scaffolding role of USP18 and expands the substrates of TRIM21, further demonstrating the complexity of ubiquitination regulatory networks in tumor progression.

TRIM proteins participate widely in cancer regulation, autoimmunity diseases, and viral infection [46]. The TRIM family is named for its N-terminal Ring domain, one to two B-box (BB) domains, and a coiled-coil (CC) domain (RBCC module). The RING domain provides E3 ligase activity, while the CC domain is crucial to form antiparallel dimers necessary to maintain enzyme activity and binding to nucleic acid chains [47]. The CC domain of TRIM28 facilitates its connection with KRAB, regulating transposable element transcription [39]. Similarly, TRIM19 oligomerization via its CC domain supports the assembly of PML nuclear bodies, enhancing their affinity for nascent DNA response elements and contributing to acute promyelocytic leukemia [48]. The BB domain function remains unclear; however, it mediates the aggregation of TRIM dimers into higher-order oligomers, as seen with TRIM72 polymerization on phospholipid membranes to aid membrane repair [40]. The structural diversity of TRIMs has attracted increased attention, highlighting them as potential therapeutic targets.

Unconventionally, TRIM29 lacks the N-terminal RING, and its B-box domain has weak catalytic activity [49]. Its ability to form dimers or oligomers was previously unknown. Herein, we found that TRIM29-WT forms dimers and oligomers. However, without its CC domain, TRIM29 could not oligomerize or enter the nucleus, although some TRIM29 remained in the nucleus even without the BB domain. These findings indicated that the CC domain primarily mediates TRIM29 dimerization and oligomerization, possibly aided by the BB domain. Our results suggest that oligomeric TRIM29 translocates into the nucleus to promote DDR signaling, which requires USP18- and TRIM21- mediated ubiquitination. However, the exact mechanism of TRIM29’s nuclear import remains to be determined.

TRIM29 was first identified in ataxia-telangiectasia cells as a negative regulator of irradiation sensitivity [27, 50]. Subsequent studies have shown that TRIM29 is crucial in DDR signaling regulation. TRIM29 promotes pancreatic cancer cell proliferation by activating Wnt/β-catenin signaling, independent of irradiation, while phosphorylation by MK2 post-irradiation enhances radioresistance [27]. In addition, TRIM29 recruits the BRCA1-associated surveillance complex, DNA-PKcs, and the TIP60 complex, to chromatin, facilitating DNA double-strand break repair [28]. TRIM29 also helps the nuclear import of RNF8, pivotal for γH2AX ubiquitination and DDR signaling promotion [41]. These findings underline TRIM29’s significant role in DDR signaling and radioresistance, as corroborated by our study. Additionally, TRIM29 influences innate and adaptive immunity by degrading STING, thus enhancing DNA virus infection [32]. In gastric cancer, TRIM29 ubiquitinates and degrades IGF2BP1, reducing *PDL1* mRNA stability to enhance antitumor immunity [51]. These diverse functions are likely associated with TRIM29’s distribution in the cytosol and nucleus, reinforcing the importance of factors influencing its localization, as supported by our identification of USP18 and TRIM21 as key components in TRIM29’s nuclear translocation and DDR signaling.

Unlike TRIM29, TRIM21 possesses a canonical N-terminal RING domain, endowing it with E3 ligase activity and its involvement in a various cellular process [46]. TRIM21 promotes K63-linked ubiquitination of CLASPIN, inhibiting CHK1 activation and leading to replication fork instability, which can result in tumorigenesis [52]. TRIM21 also ubiquitinates and degrades SNAIL, thereby restraining EMT and breast cancer progression [53]. Previously, we revealed that TRIM21 ubiquitinates and degrades VDAC2, thereby inhibiting mitochondrial DNA release in NPC [29]. The diversity of TRIM21 substrates accounts for its various functions, although its role in DDR signaling remained unclear. Herein, TRIM21 targeted and ubiquitinated TRIM29, thereby promoting its oligomerization and nuclear translocation. This facilitated DDR signaling, and expanding our understanding of TRIM21’s regulatory mechanisms in NPC progression.

Ubiquitination is involved in various cellular processes [6]. K27-linked ubiquitination, a specific subtype, plays a role in innate immunity, DNA damage repair, and signal transduction [54]. It enhances innate immunity against DNA viruses by facilitates TBK1 recruitment and its movement to perinuclear microsomes via STING ubiquitination [55]. TRIM23-mediated K27-linked ubiquitination of NEMO is essential for inflammatory responses to RNA viruses [56]. MARCH5 promotes K27-linked ubiquitination and degradation of the common cytokine receptor γ chain, impairing anti-tumor immunity [57]. RNF168 promotes K27-linked ubiquitination of H2A, crucial for DDR activation [58, 59]. Our study reveals that K27-linked ubiquitination of TRIM29 is vital for DDR in NPC, offering deeper insights into this post-translational modification’s role in irradiation-induced DDR.

In conclusion, we elucidated the essential role of the USP18-TRIM21-TRIM29 axis in regulating DDR signaling and radioresistance in NPC **(**Fig. 7F**)**. High USP18 expression correlates with poor prognosis for patients with NPC, suggesting it as a promising biomarker and target for NPC treatment.

## Materials and methods

### Clinical specimens

For proteome sequencing, five paired fresh-frozen NPC tissue samples from patients with or without relapse after radiotherapy were collected retrospectively at the Sun Yat-sen University Cancer Center (SYUCC, Guangzhou, China) from November 2016 to September 2019. All included patients were diagnosed pathologically with non-metastatic NPC, and samples were matched by sex, age, and TNM stage, as defined by the American Joint Committee on Cancer (AJCC) Cancer Staging Manual (8^th^ edition). Importantly, radiotherapy was delivered to all patients, and biopsy tissues were collected before treatment. Radiosensitive patients were defined as those with no residual lesions at 6 weeks post-radiotherapy and no recurrence within five years. Conversely, radioresistant patients exhibited residual lesions at more than 6 weeks post-radiotherapy or recurrence within two years. Table S1 shows the patients’ clinical features.

For immunohistochemical staining, 200 paraffin-embedded specimens of locoregionally advanced NPC were collected for clinical validation between November 2004 and December 2009 from SYUCC. These patients had not received any antitumor therapy before biopsy, and all samples were re-staged according to the 8^th^ AJCC manual. Details on patient clinical features are available in Table S6. The study was approved by the Institutional Ethical Review Boards of SYUCC (SL-G2023-194-01) with informed consent exempted.

### Cell culture

The study utilized NP69 normal nasopharyngeal epithelial cells; various NPC cell lines (SUNE1, HONE1, HK1, S18, 5-8 F, 6-10B, and CNE1); and HEK293T cells. As described previously [12, 29, 60–62], keratinocyte serum-free medium added with bovine pituitary extract was used to culture NP69 cells. RPMI-1640 medium (Gibco, USA) with 10% fetal bovine serum (FBS, ExCell Bio, China) was used to culture NPC cells, while HEK293T cells were maintained in DMEM (Gibco) containing 10% FBS. TRIM21-KO cells were described previously [29]. NPC cells were irradiated with a single 8 Gy fraction employing an RS-2000-PRO-225 Biological Irradiator (1.832 Gy/min; Rad Source Technologies, USA), unless otherwise specified. The cells used in the experiments were mycoplasma negative.

### Plasmids and transfection

Plasmids expressing USP18, TRIM21, and TRIM29 were constructed by cloning into phage-puro-Flag, pSin-EF2-puro or pCMV-CFP/YFP vectors. This yielded USP18-Flag, TRIM21-Flag, TRIM21-Myc, TRIM29-Myc, TRIM29-HA, USP18-YFP, TRIM21-CFP, TRIM21-YFP and TRIM29-CFP plasmids. PRK-HA-Ub and mutants were detailed in earlier works [61, 63]. The shRNA sequences targeting TRIM29 were ligated into pLKO.1-RFP to construct the PLKO.1-shTRIM29 plasmid. USP18 single guide RNAs (sgRNAs) were sourced from Benchling’s online tool (https://www.benchling.com/crispr) and cloned into pLentiCRISPRv2. The phage-puro-Flag, pSin-EF2-puro, pCMV, and pLentiCRISPRv2 empty vectors were utilized as described previously [12, 29, 60]. The shRNA, sgRNA, and siRNA sequences used in this research are listed in Table S8. Transfections were carried out employing Lipofectamine 3000 (Thermo Scientific, USA) following the supplier’s guidelines. Western blotting and quantitative real-time reverse transcription PCR (RT–qPCR) were used to assess the transfection efficiency at 24–48 h post-transfection.

### CRISPR/Cas9 gene editing

PMD2.G, PSPAX2, and pLentiCRISPRv2-sgNC or sgUSP18 plasmids were co-transfected into HEK293T cells. After 48 h of culture, the supernatant was collected, filtered through a 0.45 μm filter and centrifuged at 4000 × *g* for 30 min at 4 °C in a 50 ml concentration column. The concentrated lentivirus was stored at −80 °C. Subsequently, 40 μl of lentivirus was used to infect confluent SUNE1 and HK1 cells cultured in a 6-well plate and incubated for 48 h. Infected cells were then selected using 1 μg/ml puromycin (Beyotime, China) for 1 week and single-cell clones were isolated in 96-well plates. *USP18* knockout efficiency was confirmed using western blot and sequencing. Genomic DNA was PCR-amplified using primers flanking the sgRNA, cloned into the pClone007 vector (Tsingke biotechnology, China), and sequenced.

### Western blotting

Lysis buffer (Beyotime) containing phosphatase and protease inhibitors (Beyotime) was used for cell lysis. After centrifugation, the supernatants were heated at 95 °C for 10 min in SDS loading buffer, resolved using SDS–PAGE, and then electrophoretically transferred onto polyvinylidene fluoride membranes (Millipore, USA). Non-specific binding to the membrane was blocked using 5% w/v skim milk, followed by overnight incubation with primary antibodies at 4 °C. Following a 1 h incubation at room temperature (RT) with horseradish peroxidase (HRP)-conjugated secondary antibodies, the immunoreactive protein bands were visualized and quantified. Table S9 lists the antibodies employed.

### Co-Immunoprecipitation Assay and MS analysis

Lysis buffer was used to break the cells and incubated with primary antibodies at 4 °C overnight. Pierce^TM^ Protein A/G Magnetic Beads (Thermo Scientific) were then incubated with the immune complexes for one hour at RT to promote capture, rinsed using immunoprecipitation wash buffer and eluted with loading buffer. The eluates were subsequently resolved using SDS–PAGE, visualized using a Fast Silver Stain Kit (Beyotime), and analyzed by Wininnovate Biotechnology (China).

### Proteome sequencing

The proteome sequencing of five paired NPC tissues was conducted by Jingjie PTM BioLabs (China). Initially, the samples were lysed in a buffer containing 1% protease inhibitor cocktail, 8 M urea, 50 mM Nicotinamide, and 3 μM Trichostatin A. Subsequently, Trichloroacetic acid was added to a final concentration of 20% to precipitate the proteins, which were then washed with acetone and redissolved in 200 mM Tetraethylammonium bromide. Trypsin (Promega, USA) was added to the mixture at a mass ratio of 1:50 for overnight digestion. Next day, the sample was reduced using 5 mM dithiothreitol, alkylated using 11 mM iodoacetamide, and desalted using a Strata X solid phase extraction column.

The peptides were analyzed using liquid chromatography-tandem mass spectrometry (LC–MS/MS) as described previously [64]. The mobile phase comprised solvent A (0.1% formic acid, 2% acetonitrile) and solvent B (0.1% formic acid in acetonitrile). Peptides were separated using the NanoElute Ultra High-Pressure Liquid Chromatography (UHPLC) system (Bruker Daltonics, USA) with following solvent gradient: 0–15 min, 9%–24% B; 15–17 min, 24%–35% B; 17–19 min, 35%–80% B; 19–20 min, 80% B. The peptides were then subjected to timsTOF HT MS (Bruker Daltonics). The full MS scan range was set from 300 to 1500 m/z, and each cycle consisted of 21 Parallel Accumulation-Serial Fragmentation-MS/MS scans.

Data obtained from Data Dependent Acquisition (DDA) was analyzed using the Pulsar search engine. Tandem mass spectra were searched against the Homo_sapiens_9606_SP_20231220 Fasta database (20429 entries). Missing values in the proteomics were estimated using the random forest imputation algorithm in the missForest R package (version 1.5) [65]. Protein intensity was normalized to the average within each group. The false discovery rate for proteins, peptides, and peptide-spectrum matches was controlled at less than 1%. Differentially abundant proteins ( | fold change | ≥ 1.5 and *p* < 0.05) were identified between resistant and sensitive groups using a two-tailed Student’s *t*-test.

### Denature ubiquitin assay

Denature-immunoprecipitation assays were performed as described previously (11,54). After 24 h of co-transfection with ubiquitin, cells were lysed and denatured in 1% SDS for 5 min at 95 °C. The lysates were then processed via immunoprecipitation using anti-c-Myc Magnetic Beads (MCE, USA) at 4 °C overnight. Following the binding reaction, the bead-bound complex was washed thoroughly and eluted. The resulting eluate was analyzed using western blotting.

### Flow cytometry analysis of cell apoptosis

The apoptosis rate in each sample was assessed using a Cell Apoptosis Kit (Keygen Biotech, China). Specifically, cells were seeded in culture plate for 12 h and then subjected to 8 Gy irradiation or left entreated. After 48 h, the cells were collected, washed twice using PBS, and resuspended in 500 μl of binding buffer containing 5 μl propidium iodide (PI), and 5 μl Annexin V-FITC. Flow cytometry was conducted using a CytoFLEX flow cytometer and analyzed using CytExpert 2.2 software (both Beckman Coulter). We categorized the cells as follows: Viable cells = FITC^−^ PI^−^; early apoptotic cells = FITC^+^ PI^−^; and late apoptotic/necrotic cells = FITC^+^ PI^+^. The apoptosis rates presented include both early and late apoptotic cells.

### Clonogenic assay

SUNE1 or HK1 cells (200–10,000) were seeded into 12-well plates and exposed to a specified dose of irradiation, followed by incubation for 10–14 days to facilitate the formation of single-cell colonies. After fixation with methanol, the cells were stained with hematoxylin. Colonies comprising more than 50 cells were quantified to assess cell survival. A linear-quadratic model was employed to analyze the cell survival curves, described as: surviving fraction = exp ( − αD − βD^2^), where D represents the irradiation dose, while α and β denote the proportional constants for the linear and quadratic effect, respectively.

### In vivo animal experiment

We acquired BALB/c nude mice (female, 4–6 weeks old) from Beijing Vital River Experimental Animal Technology (China), which were housed in a specific pathogen-free facility at SYUCC. The mice were randomly assigned into each group and injected subcutaneously with 2 × 10^6^ SUNE1 cells, and the tumor volume and bodyweight were monitored every two days from day 7. When the tumor diameter reached approximately 5 mm, the tumors were treated with either 8 Gy irradiation or left untreated. The formula for the tumor volume was: length × width^2^ × 0.5, with the constraint that the largest volume did not exceed 1500 mm^3^. After 21 days, the mice were euthanized, and the tumors were excised and weighed. All tumors were subsequently processed by paraffin embedding and sectioned for further analysis. Herein, the Experimental Animal Ethics Committee of SYUCC approved the animal experiments (L025501202303010) and complied with the Declaration of Helsinki. Animal pain and distress were minimized to the greatest extent possible. The maximum tumor diameter permitted by our ethics committee was 20 mm, a limit that was not exceeded in our trial.

### Immunofluorescence

Cells were seeded onto coverslips, fixed using 4% paraformaldehyde, permeabilized employing 0.25% Triton X-100, and blocked using 3% bovine serum albumin (BSA). The cells were then incubated with primary antibodies overnight at 4 °C, followed by a 1 h incubation with secondary antibodies at RT. After Hoechst staining, the coverslips were sealed. Fluorescence images were captured using an confocal laser scanning microscope (LSM980, Germany) controlled by ZEN blue software (both Zeiss).

### FRET assay

For the FRET assay, HEK293T cells were co-transfected with either pCMV-USP18-YFP and pCMV-TRIM29-CFP, pCMV-TRIM21-YFP and pCMV-TRIM29-CFP, or pCMV-USP18-YFP and pCMV-TRIM21-CFP. After 24 h, the cells were fixed, and a Zeiss LSM980 confocal microscope was utilized to photobleach the acceptor (YFP) and quantify the intensity of the donor (CFP) in the regions where CFP and YFP colocalized. The FRET efficiency (E_FRET_) was calculated using the formula: E_FRET_ = 1 − (F_prebleach_/F_postbleach_), where F_prebleach_ and F_postbleach_ represent the initial and final fluorescence intensity of the donor (CFP).

### Nuclear and cytoplasmic extraction

The NE-PER Nuclear and Cytoplasmic Extraction Reagents (Thermo Scientific) were used to extract nuclear and cytoplasmic proteins according to the manufacturer’s instructions. Western blotting was performed to assess the levels of TRIM29 and USP18. Tubulin and fibrillarin were used as loading controls for the cytoplasmic and nuclear fractions, respectively.

### Chromatin and cytoplasmic extraction

The ChromaFlash Chromatin Extraction Kit (Epigentek, USA) was used for cell chromatin extraction according to the manufacturer’s protocol. Subsequently, western blotting was performed to evaluate the levels of TRIM29 and USP18, with Tubulin and histone H3 serving as markers for cytoplasmic and chromatin localization, respectively.

### Crosslinking-mediated oligomer assay

For the crosslinking-mediated oligomer assays, cells were harvested and lysed. To crosslink the cell lysate, either 5 mM Ethylene glycol-bis (EGS, Sangon Biotech, China) or Disuccinimidyl suberate (DSS, Thermo Scientific) were added. After incubating at RT for 15 min, the reaction was quenched using 40 mM Tris-HCl buffer for an additional 30 min. The resulting mixture was analyzed using western blotting.

### Comet assay

A Comet Assay DNA Damage Detection Kit (Keygen Biotech) was employed. Cells exposed to 8 Gy of irradiation were collected at specified time points. Then, 100 µl of 1% normal melting point agarose (NMA), melted at 90 °C, was used to cover the object slide smoothly, and then solidified at 4 °C. Next, 1 × 10^4^ cells were added with 75 µl of 0.7% low melting point agarose (LMA) at 70 °C, and overlaid on the first agarose layer. The slide was stored at 4 °C for 10 min before being covered with an additional 75 µl of 0.7% LMA as the third layer. Subsequently, the cells were lysed using lysis buffer at 4 °C for 1 h, followed by incubation in alkaline electrophoresis buffer (300 mmol/l NaOH, 1 mmol/l EDTA) at RT for 30 min. Following electrophoresis at 25 V for 20 min, the slide was neutralized in 0.4 mM Tris-HCl (pH 7.5) and stained with PI. Fluorescence images were acquired under an inverted fluorescence microscope (NIKON Eclipse Ti2-U) with excitation wavelengths of 515–560 nm. Tail moments of 20 cells per group were quantified using CaspLab-Comet Assay Software (caslab.com).

### RT-qPCR

Total RNA was extracted from the samples using RNeasy kits (Beyotime) and reverse transcribed to cDNA using M-MLV reverse transcriptase and random primers (Promega). Subsequently, qPCR analysis was conducted using SYBR Green Master mix (Thermo Scientific) on either a CFX96 Touch Real-Time PCR Detection System (Bio-Rad) or a LightCycler 480 System (Roche, Switzerland). The 2^−ΔΔCT^ method was employed to quantify relative gene expression. Table S8 details the primer sequences used.

### Immunohistochemistry

Paraffin sections were deparaffinized and rehydrated through a series of graded alcohols, preincubated with 3% hydrogen peroxide, followed by heat-induced epitope retrieval. Nonspecific binding sites were then blocked using 3% BSA before incubation with primary antibodies, followed by labeling with HRP-conjugated secondary antibodies (BOSTER, USA). The sections were then subjected to diaminobenzidine (BOSTER) staining and hematoxylin counterstaining. AxioVision Rel.4.6 computerized image analysis system (Carl Zeiss, Germany) was employed to acquire images. Two experienced pathologists assessed the sections using the immunoreactive score (IRS) system. We classified the staining intensity as: negative staining = 0, weak staining = 1, moderate staining =2, and strong staining = 3. The positive tumor cell percentage was categorized as 1 (< 10%), 2 (10–35%), 3 (35–70%), and 4 (> 70%). The staining intensity multiplied by the positive cell proportion determined the IRS score. Patients were classified into USP18 high or low group by the median of IRS score. Table S9 lists the antibodies employed for immunohistochemistry.

### Bioinformatic analysis

Differentially expressed genes (with |fold change | ≥ 2 and *p* < 0.05) between NPC tumors and normal tissues were identified in the GSE12452 [19], GSE53819 [18], and GSE180272 [20] datasets using the limma package from Bioconductor (https://www.bioconductor.org/) in R Studio. GSEA was performed on the GSE102349 dataset, comparing samples with high (*n* = 57) and low (*n* = 56) *USP18* expression, using GSEA software (version 4.2.3). Additionally, Kyoto Encyclopedia of Genes and Genomes (KEGG) pathway analysis was performed utilizing the Database for Annotation, Visualization, and Integrated Discovery (DAVID) tool (https://david.ncifcrf.gov/) and the OmicsBean workbench website (https://www.omicshare.com/).

### Molecular docking and molecular dynamic (MD) simulation

Wecomput Technology (China) was employed to perform and analyze the MD simulations and molecular docking. The full-length structures of TRIM21 and USP18 were predicted using AlphaFold2, and the structures (PDB codes: P19474 and Q9UMW8) were downloaded from Uniprot (https://www.uniprot.org/). The K165–P585 region of TRIM29 was modeled using the crystal structure of TRIM72 (PDB code: 7XT2) as a template within the Molecular Operating Environment software (MOE, Chemical Computing Group ULC, version 2022.02). All docking simulations were performed using the HDOCK server (http://hdock.phys.hust.edu.cn/), employing a hybrid algorithm integrating both template-based and template-free docking methods to predict protein-protein interactions. The interactions within the TRIM21-TRIM29-USP18 complex were further analyzed using MOE software and visualized using PyMol Version 2.5.0 (Schrödinger, LLC.).

The dimer structure of human TRIM29 (modeled without ubiquitin) was constructed based on the known dimer structure of human TRIM72. To create the model with ubiquitin, the C-terminus of the crystal structure of WT ubiquitin (PDB code: 1YJ1) was connected to K561 of the TRIM29 dimer. The MD simulations of both models were conducted using the Amber 20 package. The Amber FF14SB force field was utilized to describe standard residues, while the force field for the modified K561 residue was derived by training the lysine dipeptide, substituting the amide group in the side chain with acetamide. The partial charges were determined using the restrained electrostatic potential method at the HF/6-31 G* level with the Gaussian16 package (Gaussian, Inc.). The Transferable intermolecular potential 3 points model was chosen to describe water behavior in both models. All preparations were executed using the tleap function in Amber 20. During the MD simulations, unreasonable contacts between atoms were minimized using the conjugate gradient method. Subsequently, the heating process was conducted under the constant volume (NVT) ensemble to gradually increase the model temperature to 300 K over 100 ps. An equilibrium process under the constant pressure and constant temperature (NPT) ensemble was utilized to adjust the system density to 1 kg/l. Under NPT ensemble, 350 ns of MD simulations were carried out. Structures from the last 50 ns were selected for further binding free energy calculations using the MM-PBSA package in Amber 20.

### Statistics analysis

SPSS version 26.0 (IBM Corp.) and GraphPad Prism version 9.1.0 (GraphPad Inc.) were utilized to carry out the statistical analyses. Data from a minimum of three independent experiments are shown as the mean ± standard deviation (SD) or mean ± standard error of the mean (SEM). Statistical significance was set at *p* < 0.05. An unpaired two-tailed Student’s *t*-test was utilized to assess differences between two groups, whereas one-way ANOVA or two-way ANOVA was employed for comparisons involving multiple groups. Survival curves for distinct patient groups were constructed employing the Kaplan–Meier method, and differences between these curves were compared employing the log-rank test. Clinical characteristics across groups were evaluated using either the chi-squared (χ^2^) test or nonparametric tests as appropriate. Additionally, independent prognostic factors were identified employing univariate and multivariate Cox proportional hazards models.

## Supplementary information

Supplementary Figure

Supplementary Table

## Source data

Source data

## Data Availability

The key raw data have been uploaded to the Research Data Deposit public platform (http://www.researchdata.org.cn, RDDB2025124785) and are available from the corresponding author upon reasonable request. Mass spectrometry data of proteome sequencing (PXD056802) have been uploaded to the ProteomeXchange Consortium through the PRIDE partner repository.
