# Supplementary material for: USP18 promotes nasopharyngeal carcinoma radioresistance via TRIM29 oligomerization and ubiquitination
Source: Cell Death Differ. 2025 Nov 11;33(5):988–1003. doi: 10.1038/s41418-025-01615-3 (PMC13156314; doi:10.1038/s41418-025-01615-3)
Supplement: Supplementary file 1 — Supplementary Figure [file 41418_2025_1615_MOESM1_ESM.pdf]

Supplementary Figure

USP18 promotes nasopharyngeal carcinoma radioresistance via TRIM29 oligomerization and ubiquitination

Jia-Yi Lin et al.

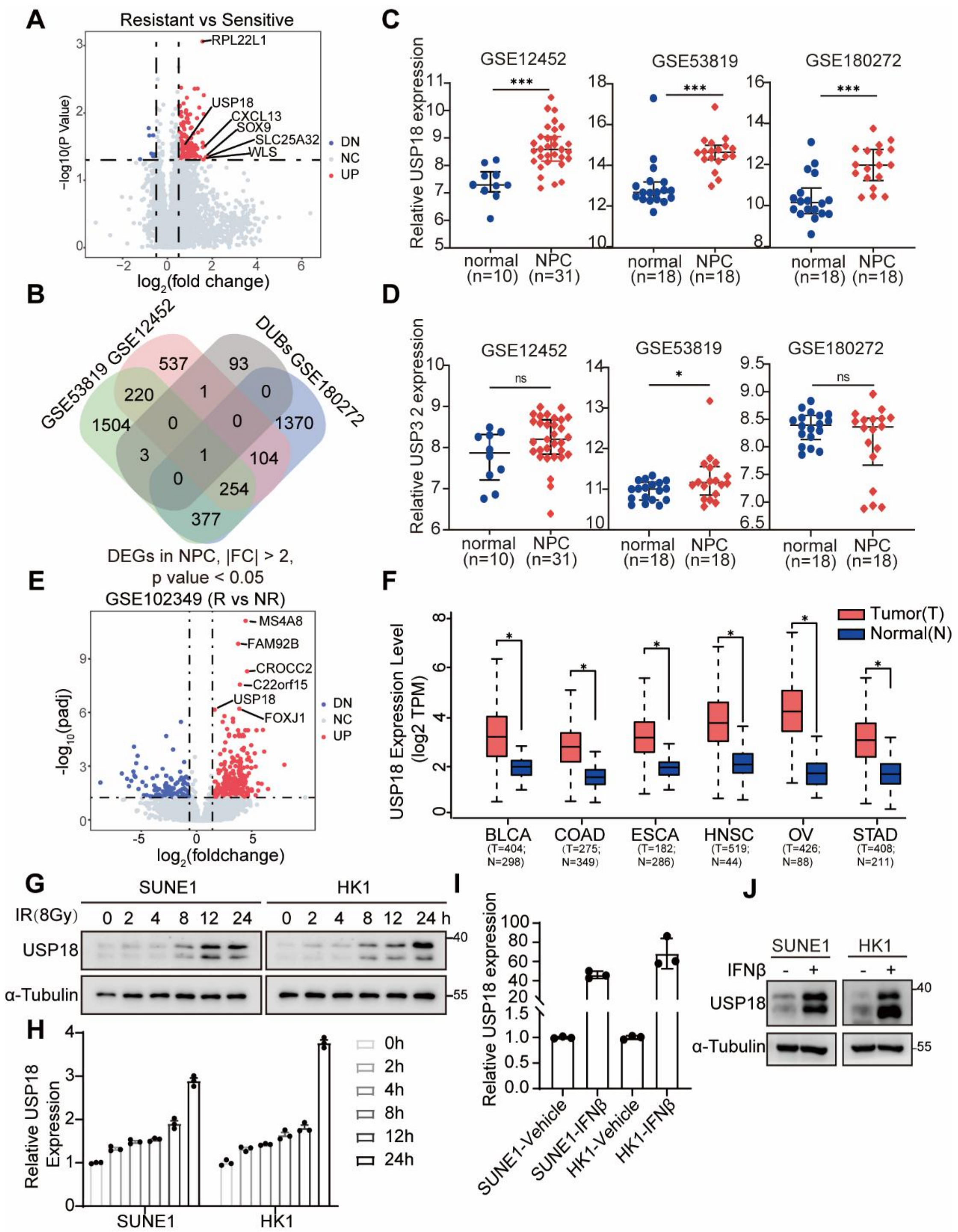

Fig. S1 USP18 is highly expressed in NPC and correlates with radiosensitivity.

A Volcanic map of differentially expressed proteins from proteome sequencing (|fold change |> 1.5 and *p* value < 0.05).

B Venn diagram of common genes among differentially expressed genes in normal and NPC tissues of GSE12452, GSE53819 and GSE180272 datasets and DUBs from iUUCD database.

C, D Relative mRNA levels of USP18 (C) and USP32 (D) in normal and NPC tissues in GSE12452, GSE53819 and GSE180272 datasets.

E Volcanic of differentially expressed genes (|fold change| > 2 and *p* value < 0.05)

between 4 paired NPC tissues with or without relapse matched by sex, age, T, N, and overall stage in GSE102349 dataset.

F The mRNA expression of USP18 in different normal and tumor tissues from TCGA database processed with Gene Expression Profiling Interactive Analysis (GEPIA).

G, H Western blot (G) and RT-qPCR (H) analyzed the protein and mRNA expression of USP18 in SUNE1 and HK1 cells treated with 8 Gy-IR for the indicated time point.

I, J RT-qPCR (I) and western blot (J) analysis of relative USP18 expression in SUNE1 and HK-1 treated with IFNβ (60ng/ml) for 24 hours.

Data were presented as the mean ± SD; $*p < 0.05$, $***p < 0.001$, ns: no significant and the *p* values were determined using the two-tailed Student's t-test. The unprocessed images of the blots are shown in Fig. S11.

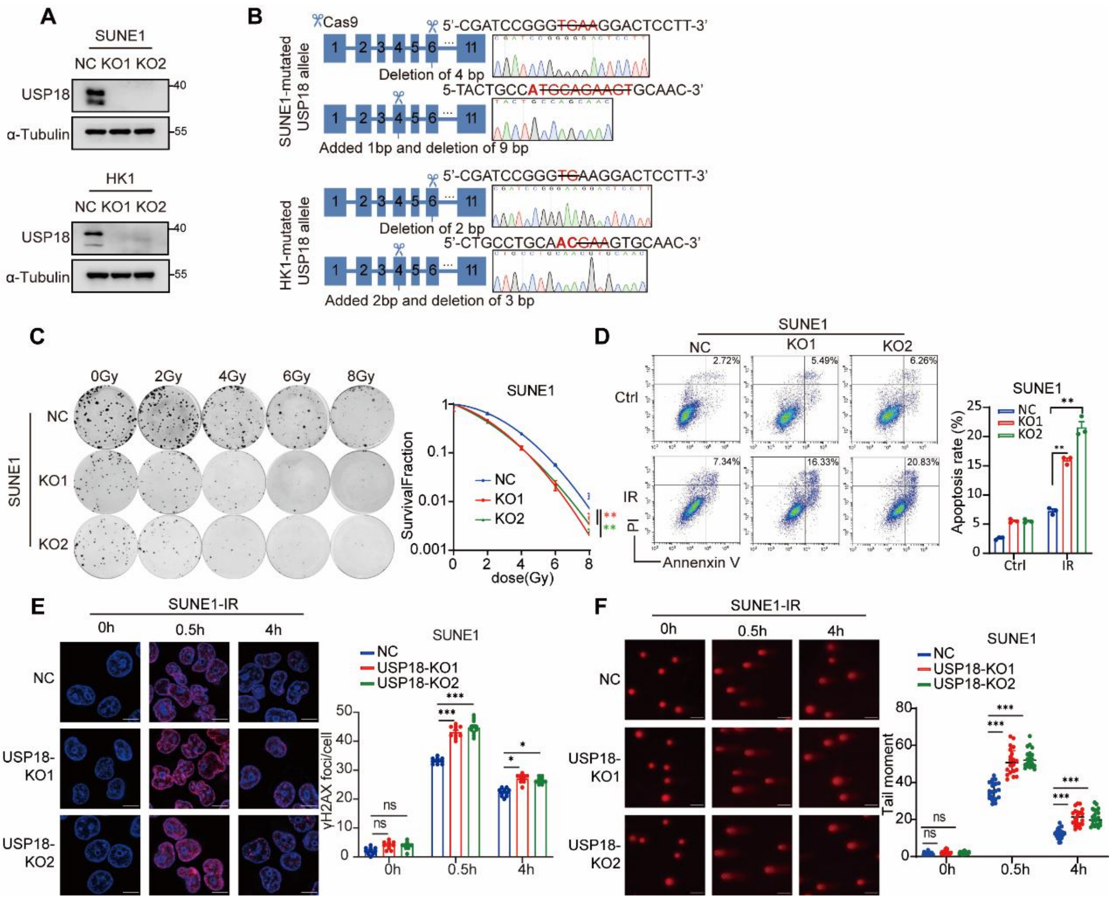

Fig. S2 The abolition of USP18 protein expression in NPC cells through CRISPR/Cas9-mediated gene editing enhances radiosensitivity.

A Western blot analyzed the expression of USP18 in NC and USP18-KO SUNE1 and HK1 cells.

B Strategy and amplicon sequencing of CRISPR/Cas9-mediated editing of human USP18 gene in SUNE1 and HK1 cells.

C Clonogenic assays and survival fraction curves of USP18-KO or NC SUNE1 cells after exposure to the indicated IR dose.

D Flow cytometry analyzing the apoptosis rate of USP18-KO or NC HK1 cells with or without exposure to 8 Gy IR after 48 h.

E, F Representative images and quantitative analysis of the number of γH2AX foci (E), comet assay and tail moments (F) in USP18-KO or NC HK1 cells treated with 8 Gy IR for 0h, 0.5h and 4h. (E)Scale bars: 10 μm, n = 10. (F) Scale bars: 100μm, n=20.

Data were presented as the mean ± SD in C-F; *$p < 0.05$, **$p < 0.01$, ***$p < 0.001$ and the *p* values were determined using the two-way ANOVA analysis. The unprocessed images of the blots are shown in Fig. S11.

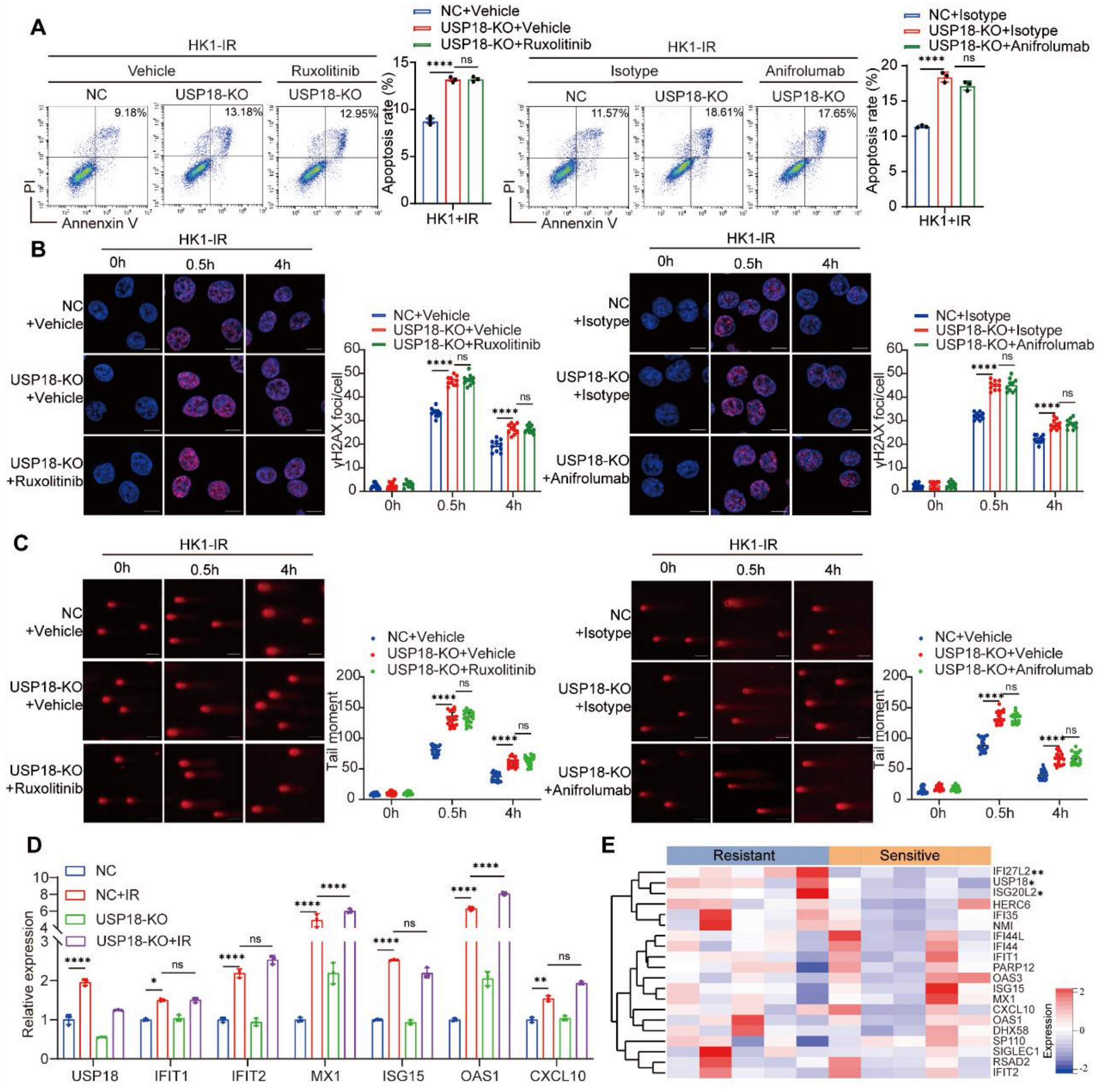

Fig. S3 USP18 impairs radiosensitivity in NPC independent of IFN-1 pathway.

A Flow cytometry analysis of apoptosis rates in USP18-KO or NC HK1 cells pre-treated with Ruxolitinib (1μM) or Anifrolumab (10 μg/ml) for 2 hours and 8 Gy IR exposure for 48 hours.

B, C Representative images and quantitative analysis of γH2AX foci (B), comet assay and tail moments (C) in USP18-KO or NC HK1 cells pre-treated with Ruxolitinib (1μM) or Anifrolumab (10 μg/ml) for 2h and 8 Gy IR exposure for 0h, 0.5h and 4h. Scale bars, 10 μm, n = 10 (B). Scale bars, 100μm, n=20 (C).

D RT-qPCR analysis of relative mRNA expression of USP18, IFIT1, IFIT2, MX1, ISG15, OAS1 and CXCL10 in USP18-KO or NC HK1 cells treated with Gy IR exposure for 48 hours.

E Heatmap of IFN induced genes identified by proteome sequencing in five paired NPC biopsy tissues from patients with (IR-resistant) or without (IR-sensitive) relapse after radiotherapy.

Data are presented as mean ± SD in A to D; *$p < 0.05$, **$p < 0.01$, ***$p < 0.001$; p values were determined using two-way ANOVA; n = 3 independent experiments.

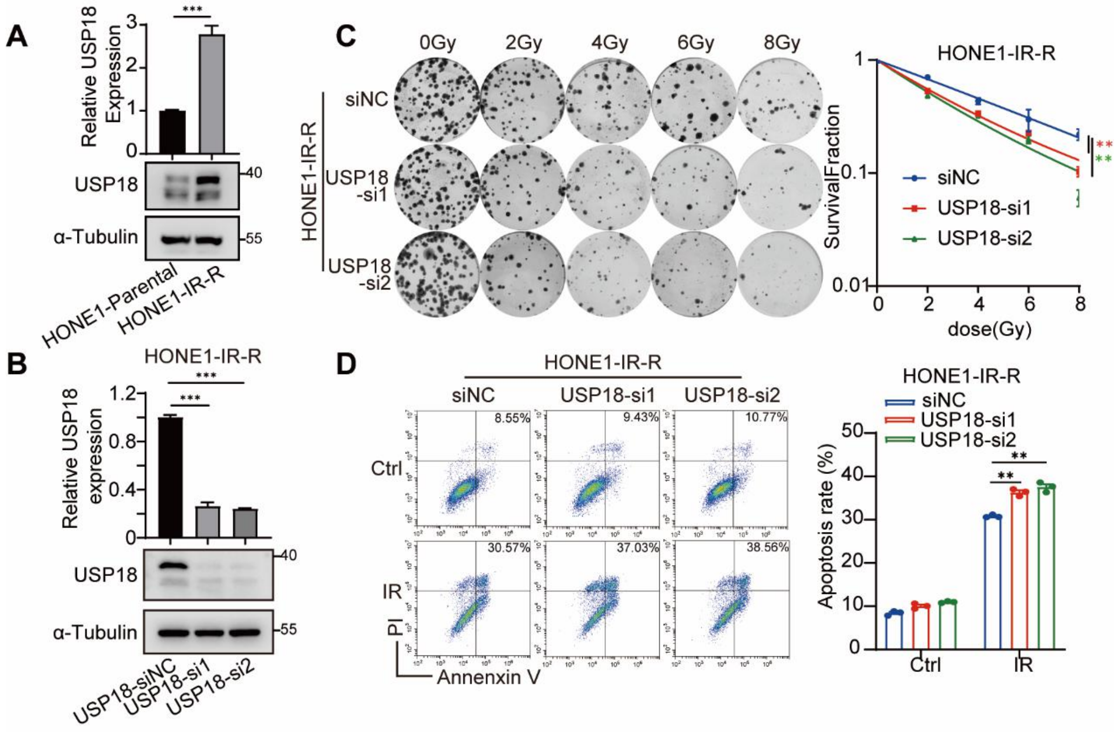

Fig. S4 USP18 is upregulated after IR and impairs radiosensitivity.

A RT-qPCR and western blot analysis of relative USP18 mRNA and protein expression in HONE1-Parental and radioresistant HONE1 cells (HONE1-IR-R).

B Western blot and RT-qPCR validated the efficiency of USP18-siRNA in HONE1-IR-R cells.

C Clonogenic assays and survival fraction curves of HONE1-IR-R cells that knocked-down of USP18 and were exposure to the indicated IR dose.

D Flow cytometry analyzing the apoptosis rate of HONE1-IR-R cells knocked-down of USP18 and were exposure with or without 8 Gy IR for 48h.

Data were presented as the mean ± SD; ***p* < 0.01, ****p* < 0.001, and the *p* values were determined using the two-way ANOVA analysis; n = 3 independent experiments. The unprocessed images of the blots are shown in Fig. S11.

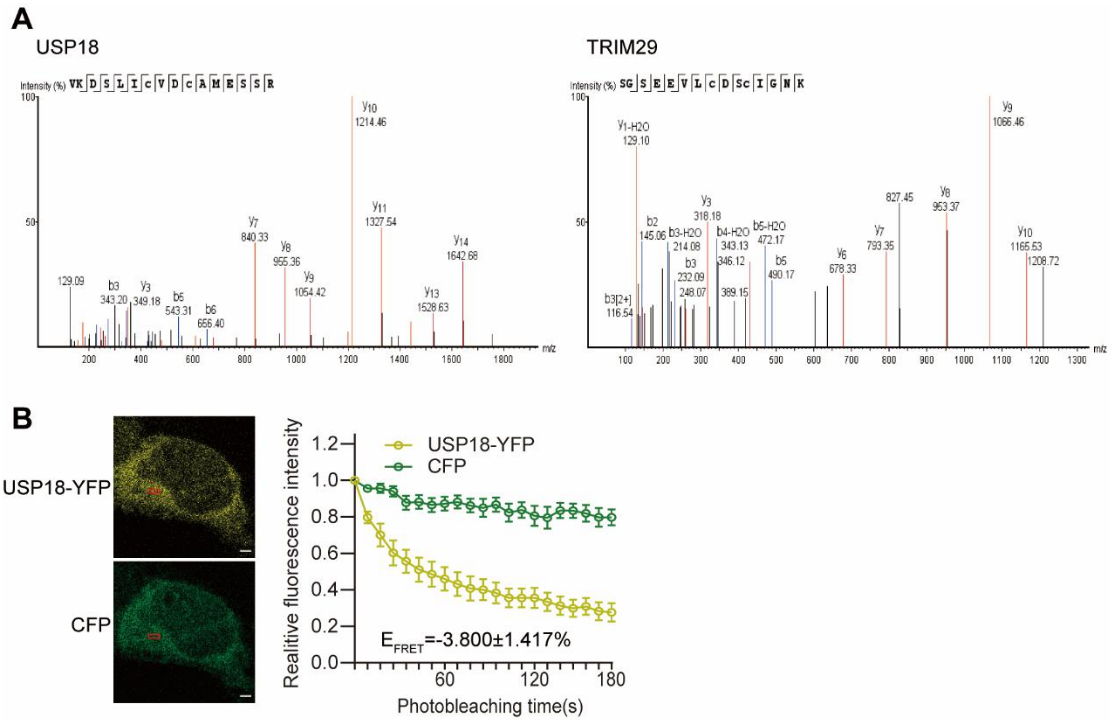

Fig. S5 USP18 interacts with TRIM29.

A Mass spectrum of USP18 and TRIM29 analyzed by the IP-MS result in SUNE1 cells transfected with USP18-Flag.

B FRET assay in HEK293T cells transfected with uncoupled-CFP and USP18-YFP and the region of FRET analysis was indicated. Scale bar, 2 μm. The decrease in USP18-YFP fluorescence and the stable uncoupled-CFP fluorescence during YFP-photobleaching in 10 cells. The FRET efficacy (E_{FRET}) was reported. The data are presented as the mean ± SEM.

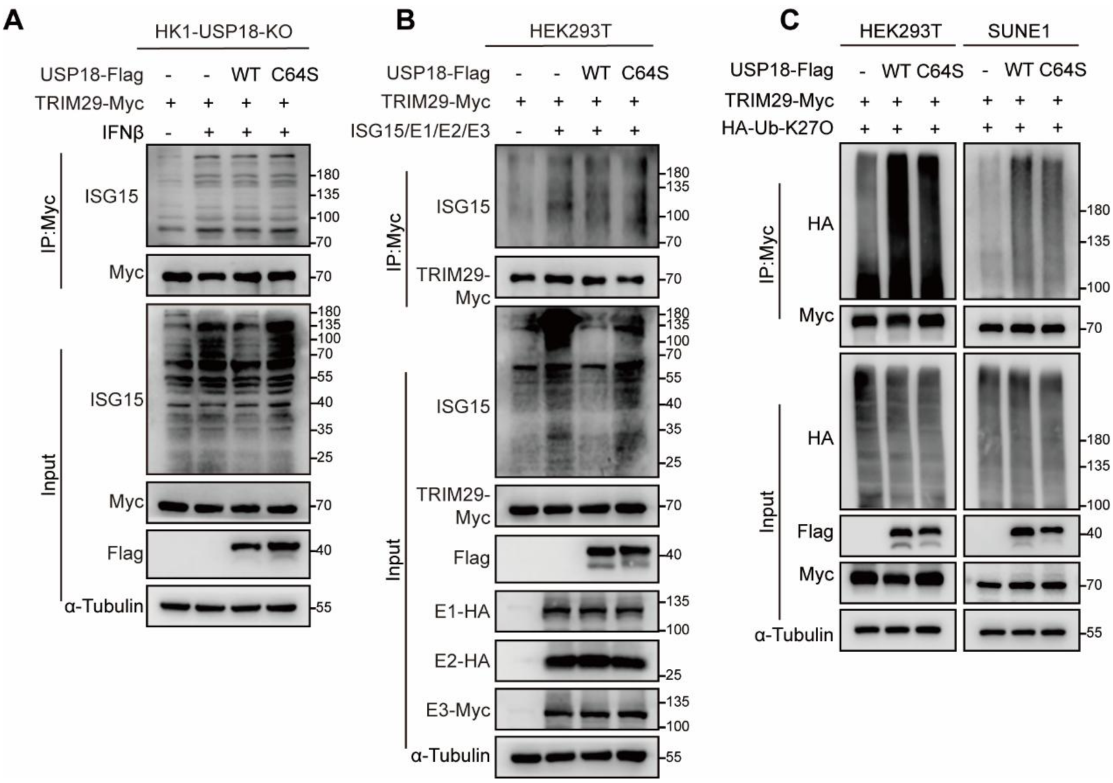

Fig. S6 USP18 promotes the K27-linked ubiquitination of TRIM29 without affecting its ISGylation.

A Co-IP assay (anti-Myc) and western blotting analysis (anti-Flag, Myc, ISG15 and α-Tubulin) in SUNE1 and HK1 USP18-KO cells transfected with TRIM29-Myc and USP18-Flag and treated with IFNβ (60ng/ml) for 24 hours.

B Co-IP assay (anti-Myc) and western blotting analysis (anti-Flag, HA, Myc, ISG15 and α-Tubulin) in HEK293T cells transfected with TRIM29-Myc, USP18-Flag, His-ISG15, UBA7-HA(E1), UBCH8-HA(E2) and HERC5-HA(E3).

C The denatured-IP assay (anti-Myc) and western blotting analysis (anti-Myc, HA, Flag and α-Tubulin) in HEK293T and SUNE1 cells transfected with TRIM29-Myc, HA-Ub-K27O plus vector or USP18- WT or C64S mutant.

The results are representative of three independent experiments. The unprocessed images of the blots are shown in Fig. S11.

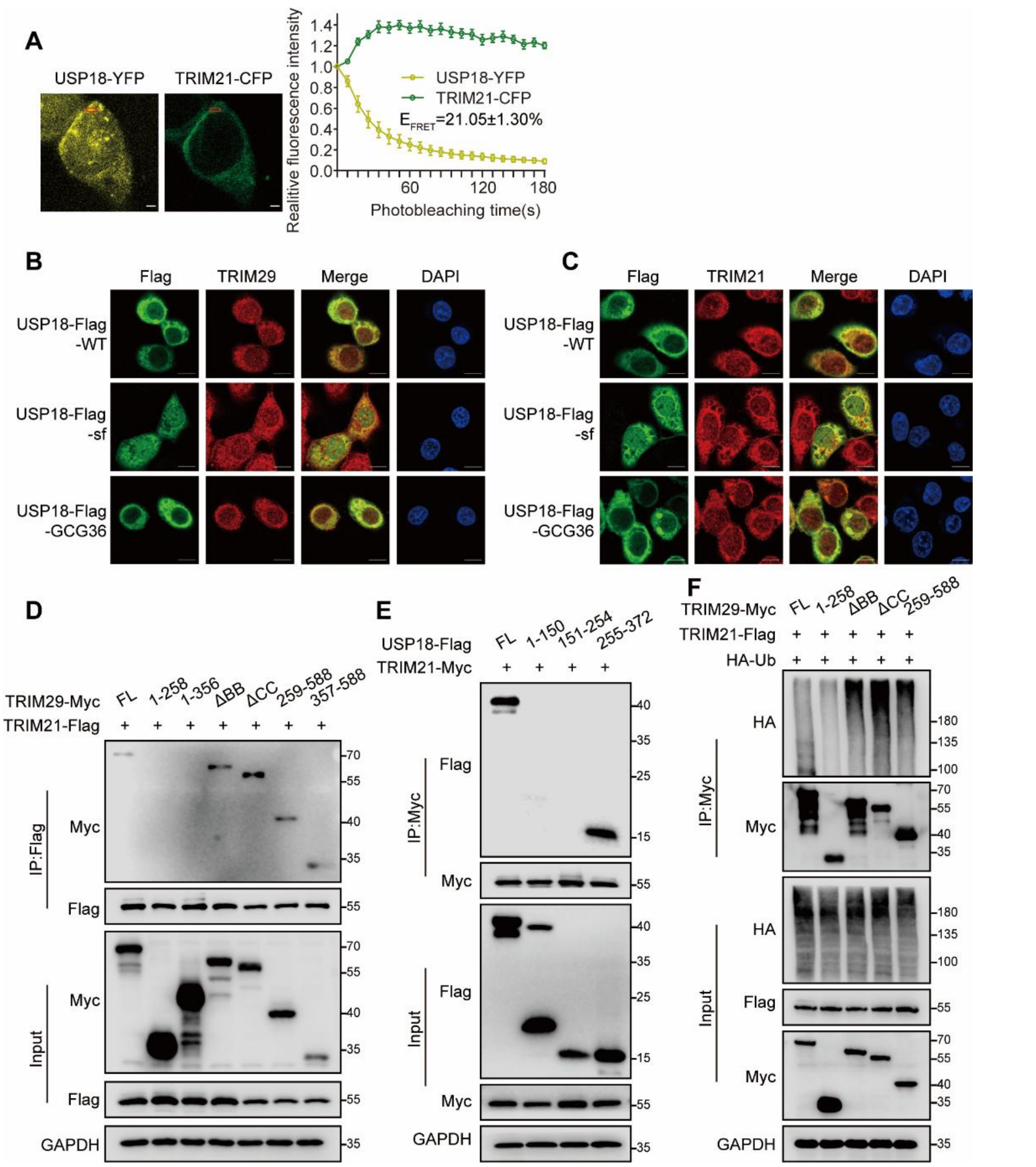

Fig. S7 USP18 recruits TRIM21 to promote the ubiquitination of TRIM29.

A The FRET assay in HEK293T cells transfected with USP18-YFP and TRIM21-CFP. Representative cells and photobleached region were indicated. The FRET efficacy (E_{FRET}) was reported. The data are presented as the mean ± SEM. Scale bar: 2 μm, n = 10.

B, C Immunofluorescence staining depicting the cellular localization of exogenous Flag-USP18 or its truncation (green) and endogenous TRIM29 (red) (B) or endogenous TRIM21 (red) (C) in HK1 USP18-KO cells. Scale bars: 10 μm.

D, E The co-IP (anti-Myc or anti-Flag) assay respectively between exogenous TRIM21 and TRIM29-Myc truncations (D) or USP18-Flag truncations (E) in HEK293T cells.

F Ubiquitination level of TRIM29-Myc truncations in HEK293T cells transfected with TRIM21-Flag and HA-Ub.

The results are representatives of three independent experiments in B-F. The unprocessed images of the blots are shown in Fig. S11.

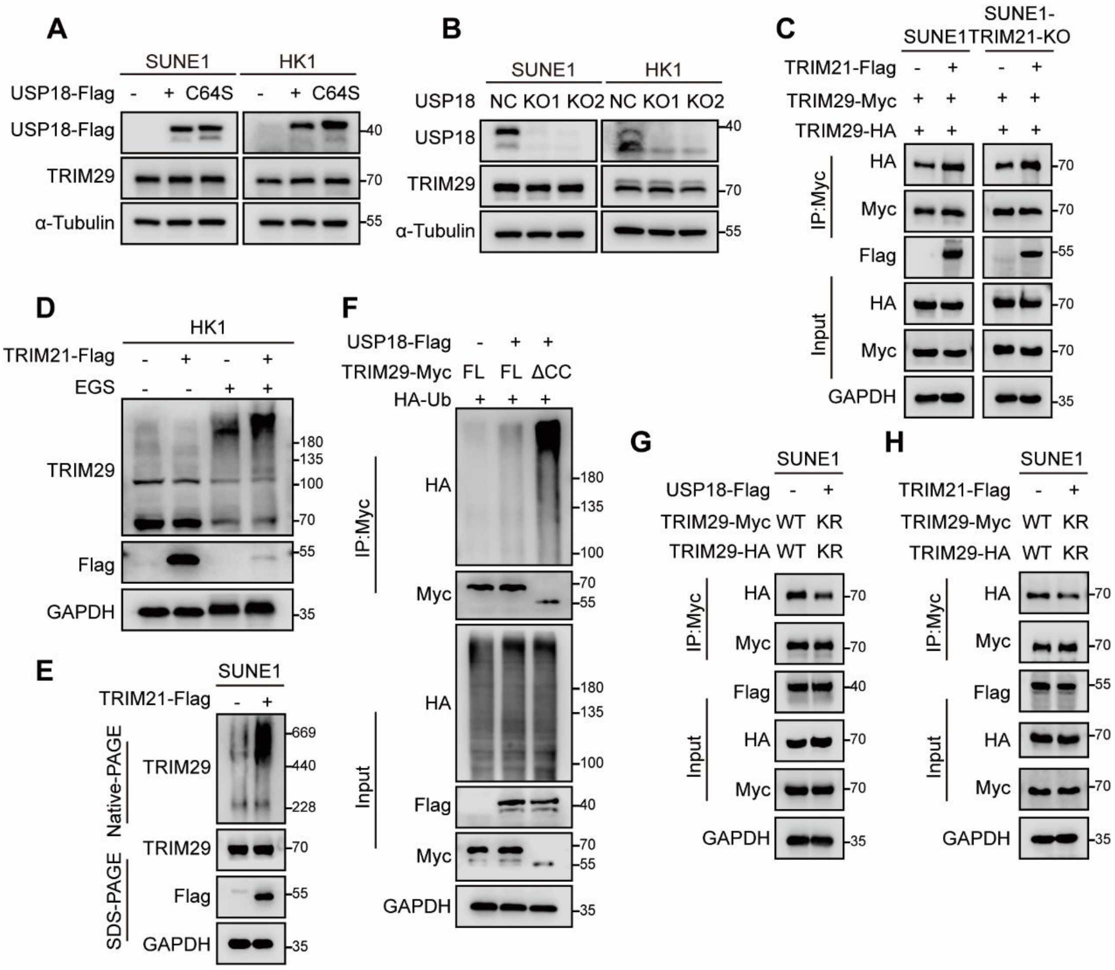

Fig. S8 The ubiquitination of TRIM29 promotes its oligomerization.

A TRIM29 and USP18 protein level in SUNE1 and HK1 cells transfected Vector, USP18- WT or C64S mutant.

B TRIM29 and USP18 protein level in USP18-KO SUNE1 and HK1 cells.

C Interaction between TRIM29 monomers (TRIM29-HA and TRIM29-Myc) in SUNE1 or SUNE1-TRIM21-KO cells with TRIM21 overexpressing or not.

D Western blot (anti-TRIM29, Flag and GAPDH) assays in HK1 cells with TRIM21 overexpressing treated with EGS.

E The protein lysis of SUNE1 cells transfected with TRIM21 was analyzed by Native-PAGE (top) and SDS-PAGE (bottom) immunoblot (anti-TRIM29, Flag and GAPDH).

F Ubiquitination level of TRIM29-WT and ΔCC mutant in HEK293T overexpressing TRIM21-Flag and HA-Ub.

G, H Interaction between TRIM29 WT (TRIM29-HA-WT and TRIM29-Myc-WT) or K561R mutant (TRIM29-HA-KR and TRIM29-Myc-KR) monomers in SUNE1 cells with USP18-overexpressing or TRIM21-overexpressing or not.

The results are representatives of three independent experiments. The unprocessed images of the blots are shown in Fig. S11.

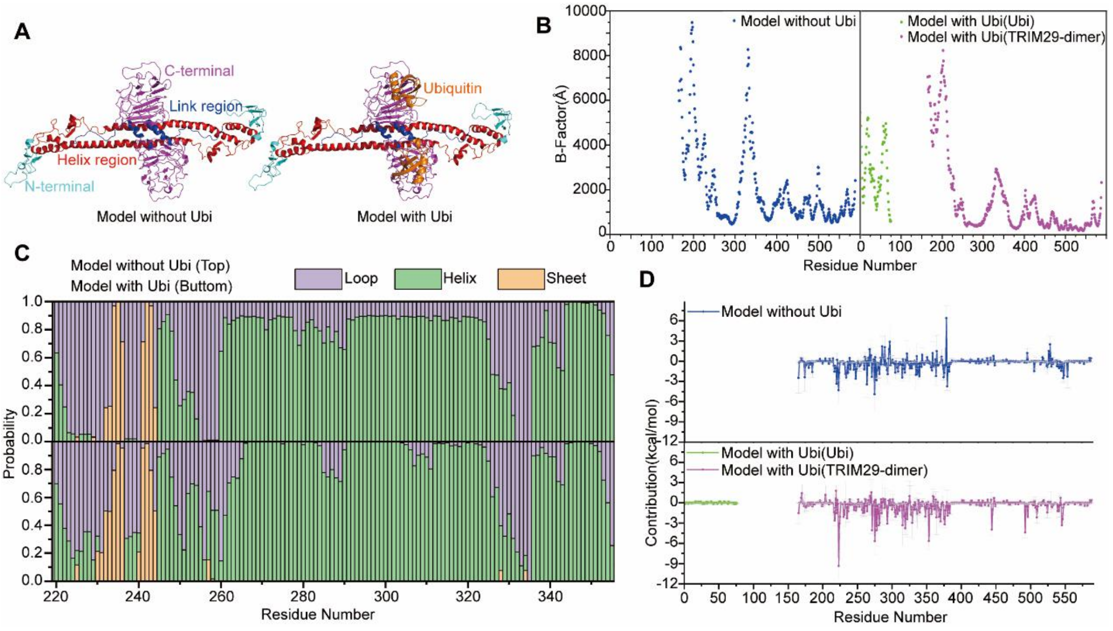

Fig. S9 Molecular dynamic simulation for TRIM29 dimer models with or without ubiquitin.

A The constructed TRIM29 dimer model with or without Ubi based on the crystal structure of TRIM72. The N-Terminal region, C-Terminal region, Helix region as well as the Link region were colored as cyan, red, blue and magenta, respectively.

B The residue B-Factor in TRIM29 dimer model with or without ubiquitin during the MD simulations, especially within the helix region (residues F220–V356 within the BB and CC domains).

C Secondary structure analysis of the Helix region in the two model during the MD simulations.

D Molecular Mechanics Poisson−Boltzmann Surface Area (MM-PBSA) calculated the binding free energies (−331.41 kcal/mol for the model with ubiquitin versus −209.16 kcal/mol without ubiquitin) and the residue energy decomposition of the two model during the MD simulation.

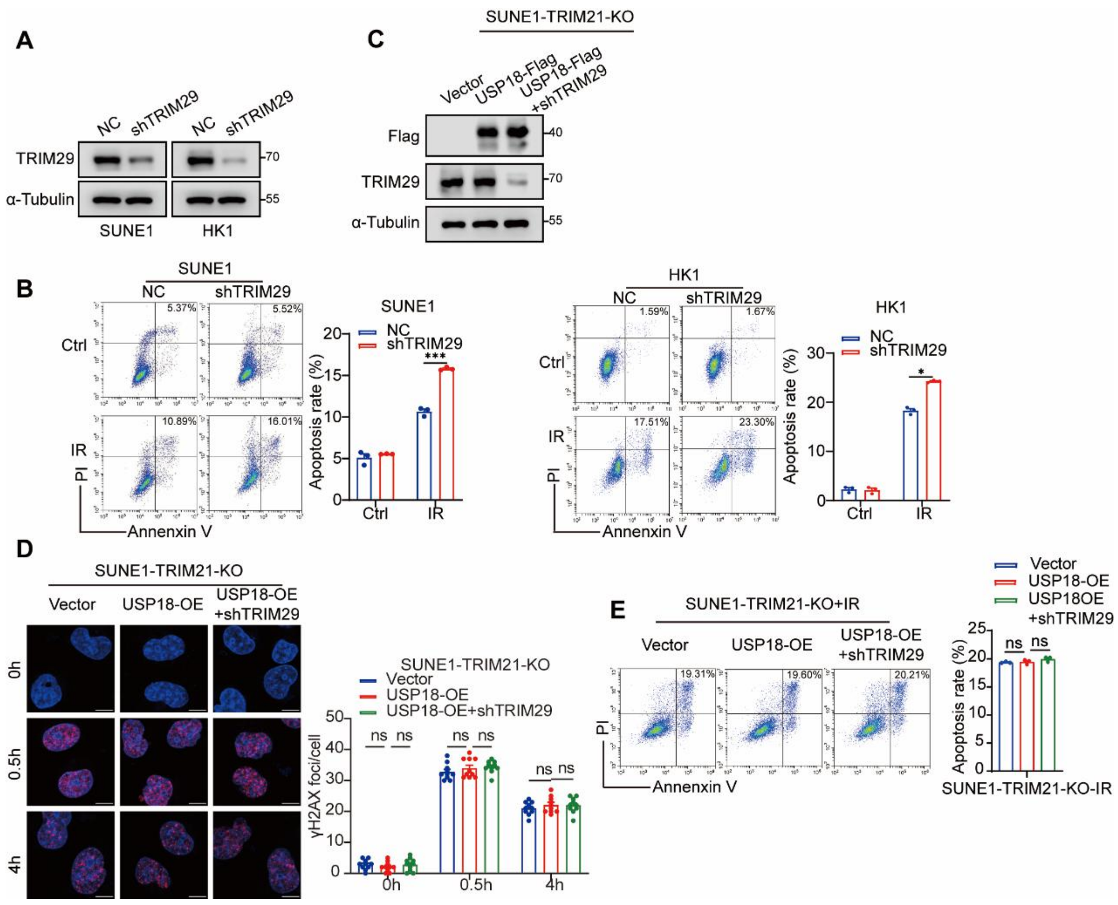

Fig. S10 USP18 impairs radiosensitivity by facilitating the DNA damage repair through TRIM29

A The protein level of TRIM29 and α-Tubulin in SUNE1 and HK1 cells stably knocked -down of TRIM29.

B Flow cytometry analyzing the apoptosis rate of SUNE1 or HK1 cells knocked-down of TRIM29 with or without exposure to 8 Gy-IR.

C The protein level of USP18-Flag, TRIM29 and α-Tubulin in TRIM21-KO SUNE1 cells transfected with vector, USP18-Flag or USP18-Flag plus shTRIM29.

D Representative images and quantitative analysis of the number of γH2AX foci in the indicated TRIM21-KO SUNE1 cells with or without 8 Gy-IR exposure. Scale bars, 10 μm, n = 10.

E Flow cytometry analyzing the apoptosis rate of indicated TRIM21-KO SUNE1cells with or without exposure to 8 Gy-IR.

Data were presented as the mean ± SD; **p* < 0.05, ****p* < 0.001, ns: no significant and the *p* values were determined using the two-tailed Student’s t-test in B or two-way ANOVA analysis in D, E; n = 3 independent experiments. The unprocessed images of the blots are shown in Fig. S11.

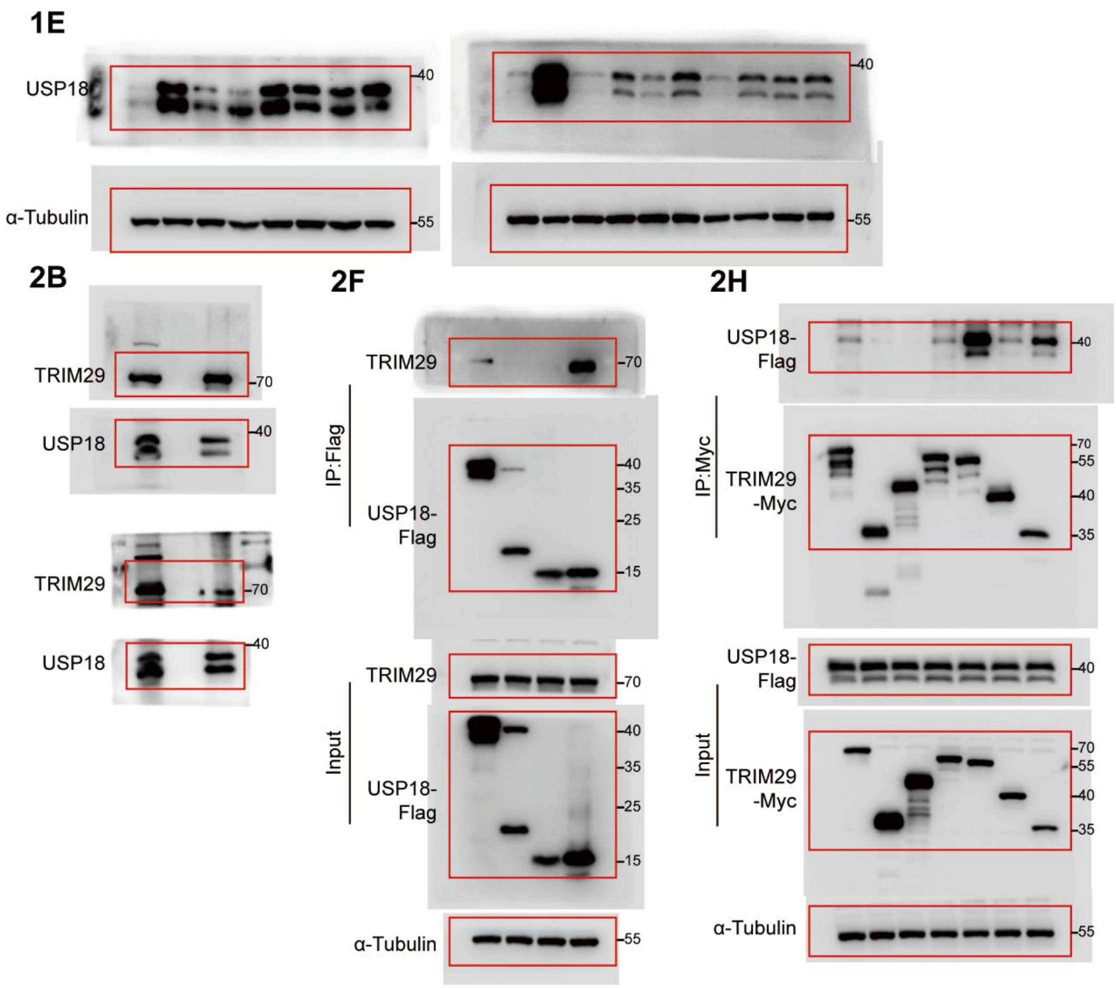

Fig. S11. (Unprocessed immunoblots)
Unprocessed immunoblots for indicated Figures panels.

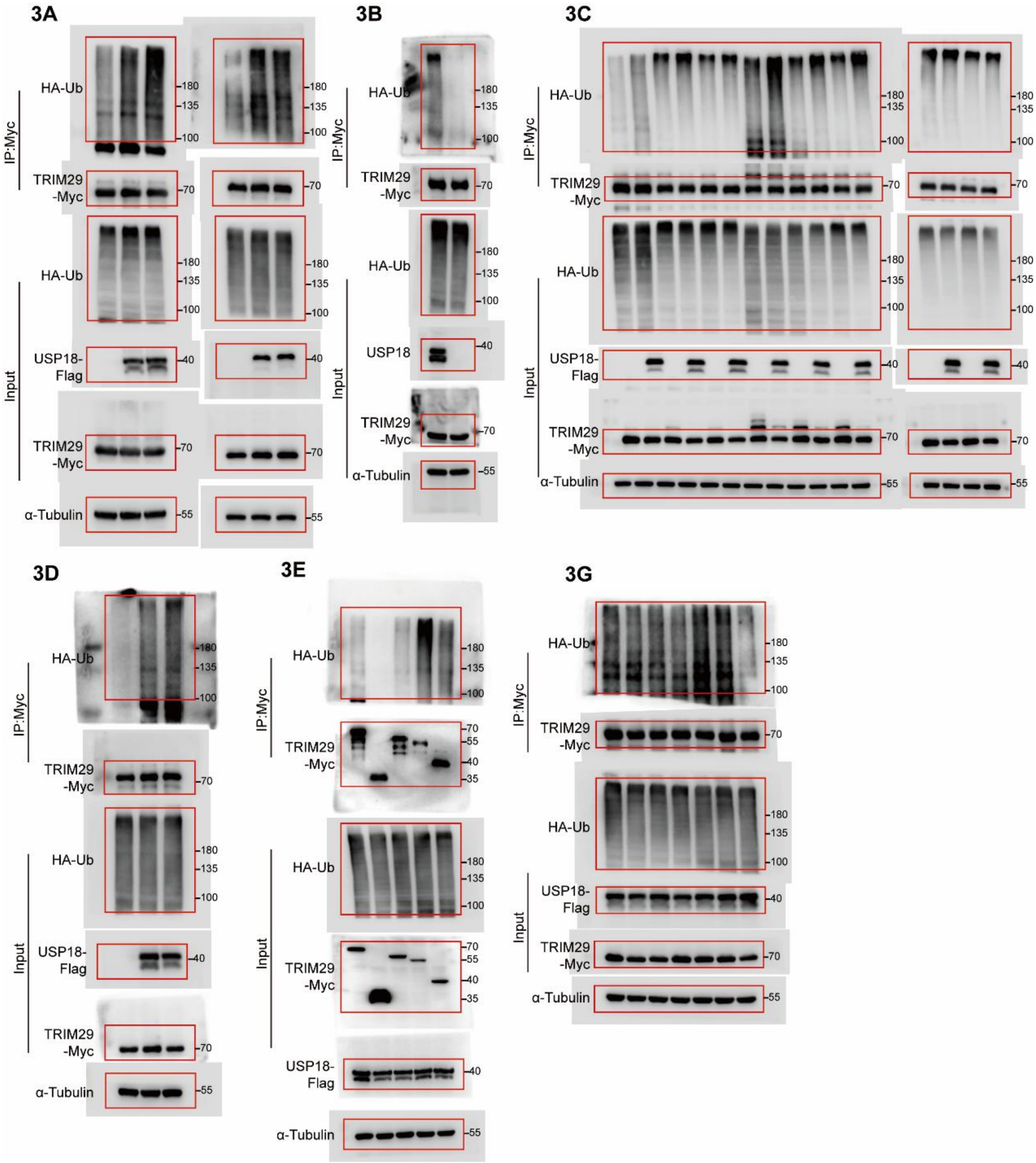

Fig. S11. (cont'd) (Unprocessed immunoblots (continued))
Unprocessed immunoblots for indicated Figures panels.

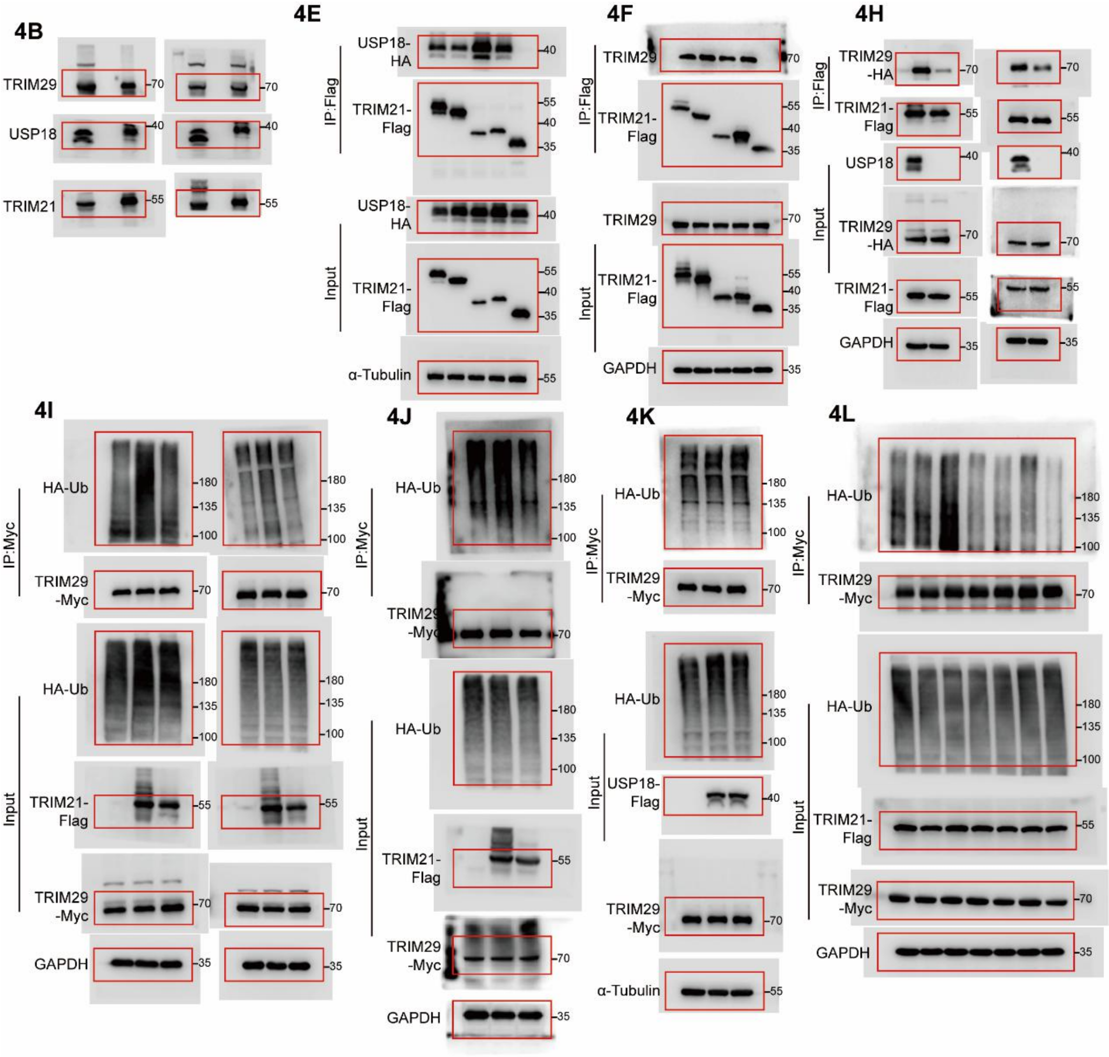

Fig. S11. (cont'd) (Unprocessed immunoblots (continued))
Unprocessed immunoblots for indicated Figures panels.

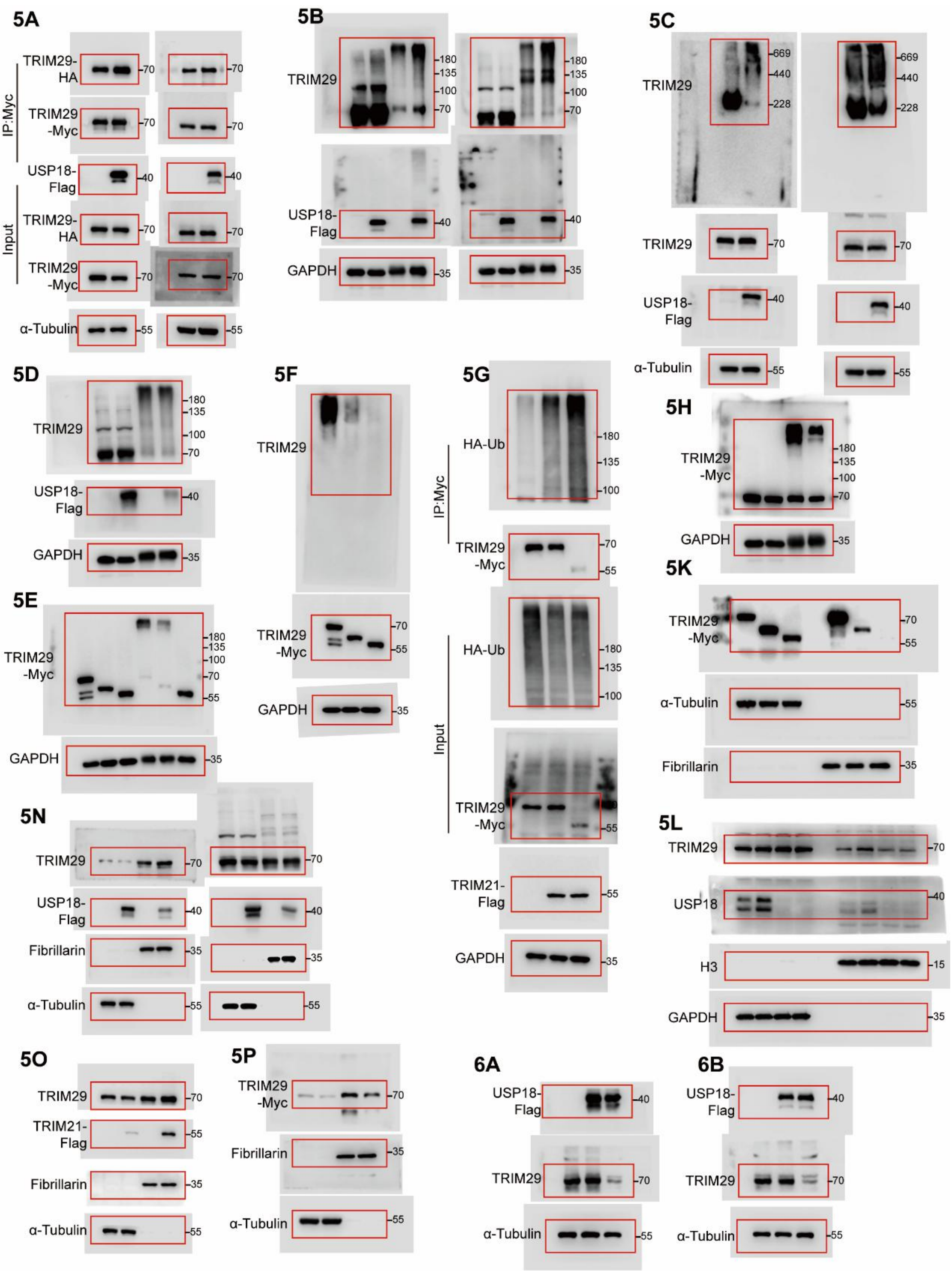

Fig. S11. (cont'd) (Unprocessed immunoblots (continued))
Unprocessed immunoblots for indicated Figures panels.

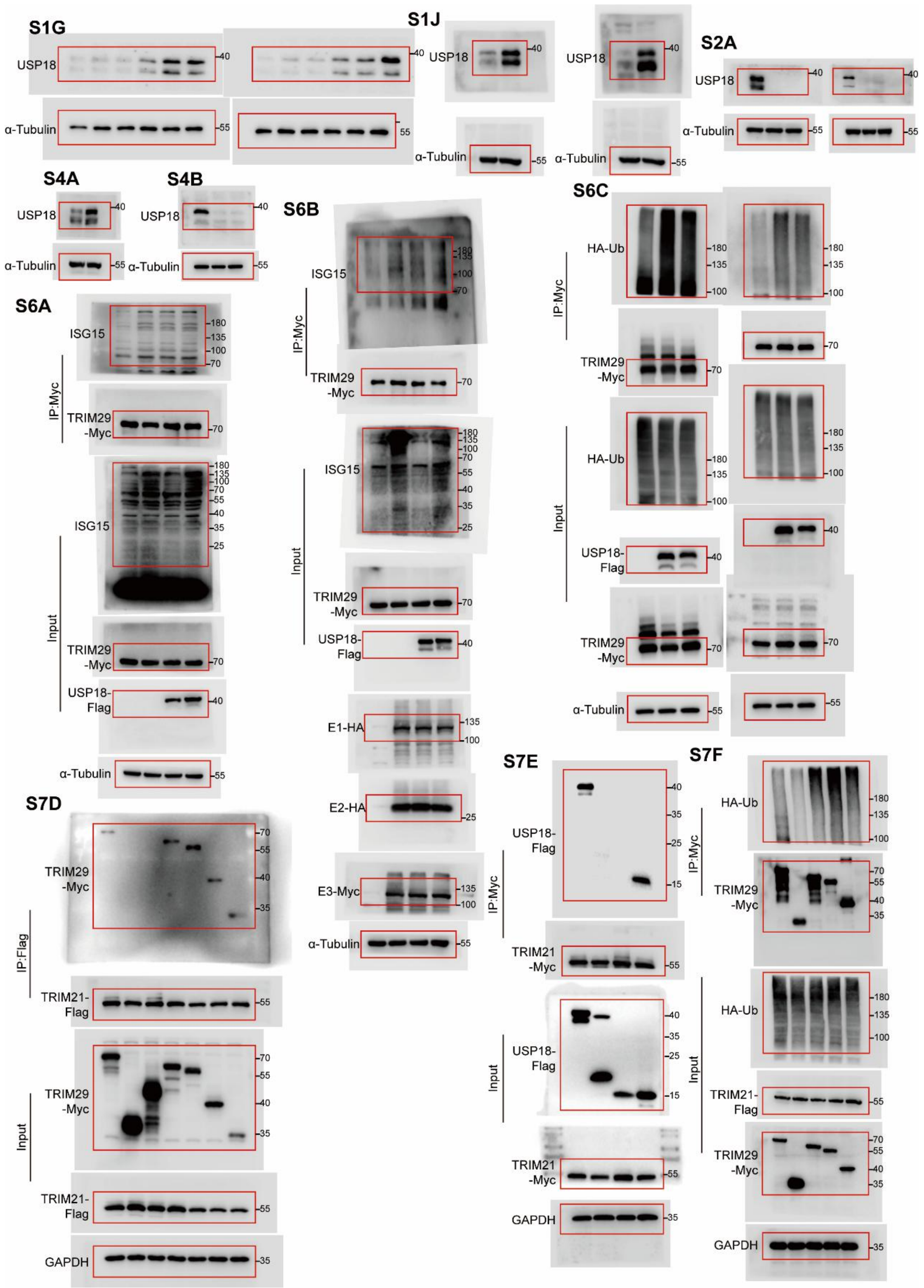

Fig. S11. (cont'd) (Unprocessed immunoblots (continued))
Unprocessed immunoblots for indicated Figures panels.

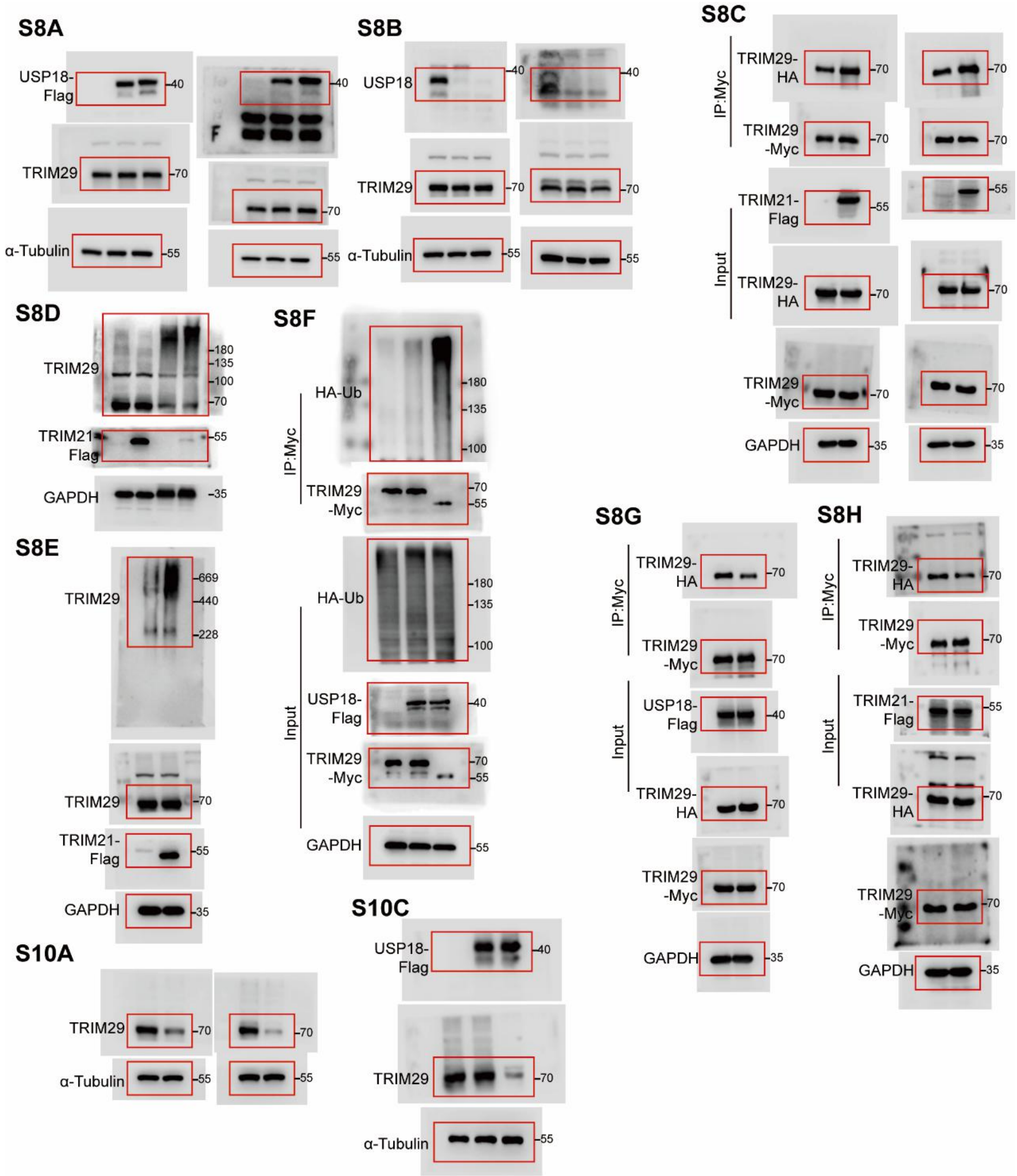

Fig. S11. (cont'd) (Unprocessed immunoblots (continued))
Unprocessed immunoblots for indicated Figures panels.
