# Supplementary material for: USP18 promotes nasopharyngeal carcinoma radioresistance via TRIM29 oligomerization and ubiquitination
Source: Cell Death Differ. 2025 Nov 11;33(5):988–1003. doi: 10.1038/s41418-025-01615-3 (PMC13156314; doi:10.1038/s41418-025-01615-3)
Supplement: Supplementary file 2 — Supplementary Table [file 41418_2025_1615_MOESM2_ESM.pdf]

# Supplementary Table

# USP18 promotes nasopharyngeal carcinoma radioresistance via TRIM29 oligomerization and ubiquitination

Jia-Yi Lin et al.

**Table S1. The clinical characteristics of 5 paired nasopharyngeal carcinoma samples.**

| Characteristic* | Resistant (n = 5) | Sensitive (n = 5) |
|---|---|---|
| Age (mean ± SD) | 37.80 ± 11.63 | 35.80 ± 10.64 |
| Sex | | |
| Male | 5 | 5 |
| T stage | | |
| T3 | 5 | 5 |
| N stage | | |
| N1 | 1 | 1 |
| N2 | 2 | 2 |
| N3 | 2 | 2 |
| TNM stage | | |
| III | 3 | 3 |
| IV | 2 | 2 |

* All patients were restaged according to the AJCC Cancer Staging Manual, 8$^{th}$ edition.

**Table S2. The results of IP-MS in SUNE1 cells that overexpressed with USP18.**

* The protein names are acquired from uniport database using the ID numbers (https://www.uniprot.org/).

| Accession* | -10 lgP | Area | Peptides | Description |
|---|---|---|---|---|
| P52732 | 187.69 | 1.87E+07 | 26 | Kinesin-like protein KIF11 OS=Homo sapiens OX=9606 GN=KIF11 PE=1 SV=2 |
| Q9UMW8 | 224.51 | 4.02E+08 | 24 | Ubl carboxyl-terminal hydrolase 18 OS=Homo sapiens OX=9606 GN=USP18 PE=1 SV=1 |
| P19474 | 205.32 | 2.70E+08 | 20 | E3 ubiquitin-protein ligase TRIM21 OS=Homo sapiens OX=9606 GN=TRIM21 PE=1 SV=1 |
| P62701 | 162.3 | 1.66E+07 | 16 | 40S ribosomal protein S4 X isoform OS=Homo sapiens OX=9606 GN=RPS4X PE=1 SV=2 |
| O95816 | 113.97 | 7.58E+06 | 8 | BAG family molecular chaperone regulator 2 OS=Homo sapiens OX=9606 GN=BAG2 PE=1 SV=1 |
| P31040 | 94.78 | 2.01E+06 | 7 | Succinate dehydrogenase [ubiquinone] flavoprotein subunit mitochondrial OS=Homo sapiens OX=9606 GN=SDHA PE=1 SV=2 |
| P09913 | 90.84 | 2.13E+06 | 8 | Interferon-induced protein with tetratricopeptide repeats 2 OS=Homo sapiens OX=9606 GN=IFIT2 PE=1 SV=1 |
| P08779 | 203.12 | 2.70E+06 | 24 | Keratin type I cytoskeletal 16 OS=Homo sapiens OX=9606 GN=KRT16 PE=1 SV=4 |
| P50914 | 121.15 | 1.11E+07 | 5 | 60S ribosomal protein L14 OS=Homo sapiens OX=9606 GN=RPL14 PE=1 SV=4 |
| Q9Y3U8 | 94.12 | 5.23E+06 | 5 | 60S ribosomal protein L36 OS=Homo sapiens OX=9606 GN=RPL36 PE=1 SV=3 |
| Q8WUM4 | 96.2 | 4.86E+06 | 5 | Programmed cell death 6-interacting protein OS=Homo sapiens OX=9606 GN=PDCD6IP PE=1 SV=1 |
| P29728 | 81.91 | 2.89E+05 | 7 | 2'-5'-oligoadenylate synthase 2 OS=Homo sapiens OX=9606 GN=OAS2 PE=1 SV=3 |
| P62266 | 113.21 | 7.26E+06 | 5 | 40S ribosomal protein S23 OS=Homo sapiens OX=9606 GN=RPS23 PE=1 SV=3 |
| Q07955 | 105.24 | 1.39E+06 | 5 | Serine/arginine-rich splicing factor 1 OS=Homo sapiens OX=9606 GN=SRSF1 PE=1 SV=2 |
| Q9UII4 | 62.74 | 7.67E+05 | 5 | E3 ISG15--protein ligase HERC5 OS=Homo sapiens OX=9606 GN=HERC5 PE=1 SV=2 |
| P43243 | 69.67 | 9.56E+05 | 4 | Matrin-3 OS=Homo sapiens OX=9606 GN=MATR3 PE=1 SV=2 |
| Q08J23 | 85.44 | 6.59E+05 | 4 | RNA cytosine C (5)-methyltransferase NSUN2 OS=Homo sapiens OX=9606 GN=NSUN2 PE=1 SV=2 |
| P62861 | 103.44 | 2.96E+07 | 3 | 40S ribosomal protein S30 OS=Homo sapiens OX=9606 GN=FAU PE=1 SV=1 |
| P35268 | 56.76 | 6.43E+06 | 3 | 60S ribosomal protein L22 OS=Homo sapiens OX=9606 GN=RPL22 PE=1 SV=2 |

| Accession | -10 lgP | Area | Peptides | Description |
|---|---|---|---|---|
| Q969Q0 | 62.15 | 6.25E+06 | 3 | 60S ribosomal protein L36a-like OS=Homo sapiens OX=9606 GN=RPL36AL PE=1 SV=3 |
| P83731 | 94.69 | 1.91E+06 | 3 | 60S ribosomal protein L24 OS=Homo sapiens OX=9606 GN=RPL24 PE=1 SV=1 |
| P61619 | 67.17 | 1.24E+06 | 3 | Protein transport protein Sec61 subunit alpha isoform 1 OS=Homo sapiens OX=9606 GN=SEC61A1 PE=1 SV=2 |
| Q8TDB6 | 71.62 | 9.38E+05 | 3 | E3 ubiquitin-protein ligase DTX3L OS=Homo sapiens OX=9606 GN=DTX3L PE=1 SV=1 |
| P53618 | 57.42 | 9.59E+05 | 3 | Coatomer subunit beta OS=Homo sapiens OX=9606 GN=COPB1 PE=1 SV=3 |
| Q14764 | 68.63 | 7.34E+05 | 3 | Major vault protein OS=Homo sapiens OX=9606 GN=MVP PE=1 SV=4 |
| P14136 | 106.65 | 2.38E+06 | 8 | Glial fibrillary acidic protein OS=Homo sapiens OX=9606 GN=GFAP PE=1 SV=1 |
| A0A075B6S2 | 140.76 | 3.90E+07 | 5 | Immunoglobulin kappa variable 2D-29 OS=Homo sapiens OX=9606 GN=IGKV2D-29 PE=3 SV=1 |
| P0C0S5 | 79.96 | 3.89E+05 | 4 | Histone H2A.Z OS=Homo sapiens OX=9606 GN=H2AZ1 PE=1 SV=2 |
| Q13751 | 85.47 | 9.96E+05 | 3 | Laminin subunit beta-3 OS=Homo sapiens OX=9606 GN=LAMB3 PE=1 SV=1 |
| Q9UQ35 | 71.44 | 5.53E+05 | 3 | Serine/arginine repetitive matrix protein 2 OS=Homo sapiens OX=9606 GN=SRRM2 PE=1 SV=2 |
| P08237 | 50.6 | 2.47E+05 | 3 | ATP-dependent 6-phosphofructokinase muscle type OS=Homo sapiens OX=9606 GN=PFKM PE=1 SV=2 |
| P46777 | 38.43 | 1.15E+06 | 2 | 60S ribosomal protein L5 OS=Homo sapiens OX=9606 GN=RPL5 PE=1 SV=3 |
| Q9NZ01 | 53.46 | 6.42E+05 | 2 | Very-long-chain enoyl-CoA reductase OS=Homo sapiens OX=9606 GN=TECR PE=1 SV=1 |
| P53985 | 51.18 | 5.92E+05 | 2 | Monocarboxylate transporter 1 OS=Homo sapiens OX=9606 GN=SLC16A1 PE=1 SV=3 |
| Q16629 | 57.14 | 5.87E+05 | 2 | Serine/arginine-rich splicing factor 7 OS=Homo sapiens OX=9606 GN=SRSF7 PE=1 SV=1 |
| P61106 | 81.34 | 8.88E+05 | 2 | Ras-related protein Rab-14 OS=Homo sapiens OX=9606 GN=RAB14 PE=1 SV=4 |
| P31689 | 67.62 | 5.30E+05 | 2 | DnaJ homolog subfamily A member 1 OS=Homo sapiens OX=9606 GN=DNAJA1 PE=1 SV=2 |
| Q5SSJ5 | 30.38 | 2.70E+05 | 2 | Heterochromatin protein 1-binding protein 3 OS=Homo sapiens OX=9606 GN=HP1BP3 PE=1 SV=1 |
| P60709 | 285.29 | 7.31E+07 | 42 | Actin cytoplasmic 1 OS=Homo sapiens OX=9606 GN=ACTB PE=1 SV=1 |
| Q14315 | 139.1 | 3.35E+05 | 8 | Filamin-C OS=Homo sapiens OX=9606 GN=FLNC PE=1 SV=3 |

| Accession | -10 lgP | Area | Peptides | Description |
|---|---|---|---|---|
| Q96SN8 | 46.31 | 1.52E+05 | 4 | CDK5 regulatory subunit-associated protein 2 OS=Homo sapiens OX=9606 GN=CDK5RAP2 PE=1 SV=5 |
| Q7Z745 | 43.49 | 0.00E+00 | 4 | Maestro heat-like repeat-containing protein family member 2B OS=Homo sapiens OX=9606 GN=MROH2B PE=2 SV=3 |
| A4UGR9 | 44.13 | 1.88E+06 | 3 | Xin actin-binding repeat-containing protein 2 OS=Homo sapiens OX=9606 GN=XIRP2 PE=1 SV=2 |
| Q9NUK0 | 49.41 | 1.76E+06 | 3 | Muscleblind-like protein 3 OS=Homo sapiens OX=9606 GN=MBNL3 PE=1 SV=2 |
| Q8WZ64 | 34.05 | 5.02E+05 | 3 | Arf-GAP with Rho-GAP domain ANK repeat and PH domain-containing protein 2 OS=Homo sapiens OX=9606 GN=ARAP2 PE=1 SV=3 |
| Q8IZT6 | 38.61 | 2.59E+05 | 3 | Abnormal spindle-like microcephaly-associated protein OS=Homo sapiens OX=9606 GN=ASPM PE=1 SV=2 |
| P28288 | 37.03 | 7.19E+04 | 3 | ATP-binding cassette sub-family D member 3 OS=Homo sapiens OX=9606 GN=ABCD3 PE=1 SV=1 |
| Q52LJ0 | 43.73 | 3.02E+04 | 3 | Protein FAM98B OS=Homo sapiens OX=9606 GN=FAM98B PE=1 SV=2 |
| Q8NCX0 | 45.43 | 3.93E+07 | 2 | Coiled-coil domain-containing protein 150 OS=Homo sapiens OX=9606 GN=CCDC150 PE=1 SV=2 |
| P06310 | 77.13 | 3.19E+06 | 2 | Immunoglobulin kappa variable 2-30 OS=Homo sapiens OX=9606 GN=IGKV2-30 PE=3 SV=2 |
| Q96FS4 | 42.84 | 2.49E+06 | 2 | Signal-induced proliferation-associated protein 1 OS=Homo sapiens OX=9606 GN=SIPA1 PE=1 SV=1 |
| A2VDJ0 | 28.37 | 1.67E+06 | 2 | Transmembrane protein 131-like OS=Homo sapiens OX=9606 GN=TMEM131L PE=1 SV=2 |
| P60763 | 48.4 | 6.93E+05 | 2 | Ras-related C3 botulinum toxin substrate 3 OS=Homo sapiens OX=9606 GN=RAC3 PE=1 SV=1 |
| Q9NZI8 | 45.31 | 5.95E+05 | 2 | Insulin-like growth factor 2 mRNA-binding protein 1 OS=Homo sapiens OX=9606 GN=IGF2BP1 PE=1 SV=2 |
| P18085 | 63.02 | 4.36E+05 | 2 | ADP-ribosylation factor 4 OS=Homo sapiens OX=9606 GN=ARF4 PE=1 SV=3 |
| Q9BXP5 | 44.78 | 3.61E+05 | 2 | Serrate RNA effector molecule homolog OS=Homo sapiens OX=9606 GN=SRRT PE=1 SV=1 |
| P56192 | 39.31 | 2.30E+05 | 2 | Methionine--tRNA ligase cytoplasmic OS=Homo sapiens OX=9606 GN=MARS1 PE=1 SV=2 |
| Q9UQP3 | 26.92 | 2.20E+05 | 2 | Tenascin-N OS=Homo sapiens OX=9606 GN=TNN PE=1 SV=2 |
| O00629 | 31.93 | 1.99E+05 | 2 | Importin subunit alpha-3 OS=Homo sapiens OX=9606 GN=KPNA4 PE=1 SV=1 |

| Accession | -10 lgP | Area | Peptides | Description |
|---|---|---|---|---|
| P22314 | 53.89 | 1.77E+05 | 2 | Ubiquitin-like modifier-activating enzyme 1 OS=Homo sapiens OX=9606 GN=UBA1 PE=1 SV=3 |
| Q8IXQ6 | 41.01 | 1.43E+05 | 2 | Protein mono-ADP-ribosyltransferase PARP9 OS=Homo sapiens OX=9606 GN=PARP9 PE=1 SV=2 |
| P06493 | 45.51 | 1.35E+05 | 2 | Cyclin-dependent kinase 1 OS=Homo sapiens OX=9606 GN=CDK1 PE=1 SV=3 |
| Q8IY18 | 34.97 | 1.01E+05 | 2 | Structural maintenance of chromosomes protein 5 OS=Homo sapiens OX=9606 GN=SMC5 PE=1 SV=2 |
| P46940 | 30.81 | 5.48E+04 | 2 | Ras GTPase-activating-like protein IQGAP1 OS=Homo sapiens OX=9606 GN=IQGAP1 PE=1 SV=1 |
| P01859 | 29.54 | 4.13E+06 | 1 | Immunoglobulin heavy constant gamma 2 OS=Homo sapiens OX=9606 GN=IGHG2 PE=1 SV=2 |
| A6NHT5 | 37.85 | 1.52E+06 | 1 | Homeobox protein HMX3 OS=Homo sapiens OX=9606 GN=HMX3 PE=1 SV=1 |
| P60468 | 36.75 | 6.19E+05 | 1 | Protein transport protein Sec61 subunit beta OS=Homo sapiens OX=9606 GN=SEC61B PE=1 SV=2 |
| Q99569 | 20.86 | 6.17E+05 | 1 | Plakophilin-4 OS=Homo sapiens OX=9606 GN=PKP4 PE=1 SV=2 |
| Q9UM00 | 24.98 | 3.08E+05 | 1 | Calcium load-activated calcium channel OS=Homo sapiens OX=9606 GN=TMCO1 PE=1 SV=2 |
| P62633 | 34.96 | 2.95E+05 | 1 | CCHC-type zinc finger nucleic acid binding protein OS=Homo sapiens OX=9606 GN=CNBP PE=1 SV=1 |
| P24539 | 23.9 | 2.18E+05 | 1 | ATP synthase F (0) complex subunit B1 mitochondrial OS=Homo sapiens OX=9606 GN=ATP5PB PE=1 SV=2 |
| P63208 | 49.93 | 2.09E+05 | 1 | S-phase kinase-associated protein 1 OS=Homo sapiens OX=9606 GN=SKP1 PE=1 SV=2 |
| P16885 | 23.25 | 1.69E+05 | 1 | 1-phosphatidylinositol 4 5-bisphosphate phosphodiesterase gamma-2 OS=Homo sapiens OX=9606 GN=PLCG2 PE=1 SV=4 |
| Q03252 | 26.54 | 1.66E+05 | 1 | Lamin-B2 OS=Homo sapiens OX=9606 GN=LMNB2 PE=1 SV=4 |
| P19623 | 33.22 | 1.40E+05 | 1 | Spermidine synthase OS=Homo sapiens OX=9606 GN=SRM PE=1 SV=1 |
| P49750 | 32.22 | 1.19E+05 | 1 | YLP motif-containing protein 1 OS=Homo sapiens OX=9606 GN=YLPM1 PE=1 SV=4 |
| P22234 | 27.76 | 1.06E+05 | 1 | Multifunctional protein ADE2 OS=Homo sapiens OX=9606 GN=PAICS PE=1 SV=3 |
| P62070 | 32.18 | 9.85E+04 | 1 | Ras-related protein R-Ras2 OS=Homo sapiens OX=9606 GN=RRAS2 PE=1 SV=1 |
| P25398 | 38.68 | 8.47E+04 | 1 | 40S ribosomal protein S12 OS=Homo sapiens OX=9606 GN=RPS12 PE=1 SV=3 |

| Accession | -10 lgP | Area | Peptides | Description |
|---|---|---|---|---|
| Q92974 | 28.06 | 7.38E+04 | 1 | Rho guanine nucleotide exchange factor 2 OS=Homo sapiens OX=9606 GN=ARHGEF2 PE=1 SV=4 |
| P62195 | 23.96 | 7.20E+04 | 1 | 26S proteasome regulatory subunit 8 OS=Homo sapiens OX=9606 GN=PSMC5 PE=1 SV=1 |
| Q86X55 | 30.33 | 1.85E+05 | 1 | Histone-arginine methyltransferase CARM1 OS=Homo sapiens OX=9606 GN=CARM1 PE=1 SV=3 |

**Table S3. The results of IP-MS in SUNE1 cells that overexpressed with TRIM29.**

* The protein names are acquired from uniport database using the ID numbers (https://www.uniprot.org/).

| Accession* | -10 lgP | Area | Peptides | Description |
|---|---|---|---|---|
| Q15149 | 446.81 | 3.11E+09 | 346 | Plectin OS=Homo sapiens OX=9606 GN=PLEC PE=1 SV=3 |
| P15924 | 334.19 | 2.62E+08 | 157 | Desmoplakin OS=Homo sapiens OX=9606 GN=DSP PE=1 SV=3 |
| P35580 | 344.98 | 1.57E+08 | 109 | Myosin-10 OS=Homo sapiens OX=9606 GN=MYH10 PE=1 SV=3 |
| Q13813 | 321.55 | 9.51E+07 | 107 | Spectrin alpha chain non-erythrocytic 1 OS=Homo sapiens OX=9606 GN=SPTAN1 PE=1 SV=3 |
| Q01082 | 304.14 | 7.70E+07 | 93 | Spectrin beta chain non-erythrocytic 1 OS=Homo sapiens OX=9606 GN=SPTBN1 PE=1 SV=2 |
| P78527 | 277.07 | 3.90E+07 | 73 | DNA-dependent protein kinase catalytic subunit OS=Homo sapiens OX=9606 GN=PRKDC PE=1 SV=3 |
| Q8WWI1 | 333.94 | 3.84E+08 | 70 | LIM domain only protein 7 OS=Homo sapiens OX=9606 GN=LMO7 PE=1 SV=3 |
| P21333 | 295.36 | 7.27E+07 | 65 | Filamin-A OS=Homo sapiens OX=9606 GN=FLNA PE=1 SV=4 |
| Q14134 | 320.31 | 4.62E+09 | 60 | Tripartite motif-containing protein 29 OS=Homo sapiens OX=9606 GN=TRIM29 PE=1 SV=2 |
| P60709 | 310.22 | 2.08E+08 | 51 | Actin cytoplasmic 1 OS=Homo sapiens OX=9606 GN=ACTB PE=1 SV=1 |
| P63261 | 310.13 | 7.59E+07 | 51 | Actin cytoplasmic 2 OS=Homo sapiens OX=9606 GN=ACTG1 PE=1 SV=1 |
| Q86V48 | 249.23 | 4.91E+07 | 39 | Leucine zipper protein 1 OS=Homo sapiens OX=9606 GN=LUZP1 PE=1 SV=2 |
| Q6ZRV2 | 263.93 | 5.87E+07 | 37 | Protein FAM83H OS=Homo sapiens OX=9606 GN=FAM83H PE=1 SV=3 |
| Q14573 | 214.09 | 2.61E+07 | 36 | Inositol 1 4 5-trisphosphate receptor type 3 OS=Homo sapiens OX=9606 GN=ITPR3 PE=1 SV=2 |
| Q9UHB6 | 239.39 | 1.05E+08 | 32 | LIM domain and actin-binding protein 1 OS=Homo sapiens OX=9606 GN=LIMA1 PE=1 SV=1 |
| Q14204 | 195.78 | 9.75E+06 | 32 | Cytoplasmic dynein 1 heavy chain 1 OS=Homo sapiens OX=9606 GN=DYNC1H1 PE=1 SV=5 |
| Q8WWM7 | 218.21 | 1.09E+08 | 30 | Ataxin-2-like protein OS=Homo sapiens OX=9606 GN=ATXN2L PE=1 SV=2 |
| P27708 | 244.2 | 2.88E+07 | 30 | CAD protein OS=Homo sapiens OX=9606 GN=CAD PE=1 SV=3 |
| Q00610 | 228.04 | 2.00E+07 | 29 | Clathrin heavy chain 1 OS=Homo sapiens OX=9606 GN=CLTC PE=1 SV=5 |

| Accession | -10 lgP | Area | Peptides | Description |
|---|---|---|---|---|
| Q6WCQ1 | 230.23 | 3.08E+07 | 28 | Myosin phosphatase Rho-interacting protein OS=Homo sapiens OX=9606 GN=MPRIP PE=1 SV=3 |
| O14974 | 190.84 | 2.73E+07 | 27 | Protein phosphatase 1 regulatory subunit 12A OS=Homo sapiens OX=9606 GN=PPP1R12A PE=1 SV=1 |
| P17987 | 231.97 | 9.42E+07 | 27 | T-complex protein 1 subunit alpha OS=Homo sapiens OX=9606 GN=TCP1 PE=1 SV=1 |
| P52272 | 232.05 | 6.63E+07 | 26 | Heterogeneous nuclear ribonucleoprotein M OS=Homo sapiens OX=9606 GN=HNRNPM PE=1 SV=3 |
| P17844 | 208.56 | 1.93E+07 | 25 | Probable ATP-dependent RNA helicase DDX5 OS=Homo sapiens OX=9606 GN=DDX5 PE=1 SV=1 |
| Q16891 | 203.83 | 3.85E+07 | 25 | MICOS complex subunit MIC60 OS=Homo sapiens OX=9606 GN=IMMT PE=1 SV=1 |
| Q09666 | 180.08 | 7.49E+06 | 24 | Neuroblast differentiation-associated protein AHNAK OS=Homo sapiens OX=9606 GN=AHNAK PE=1 SV=2 |
| P61978 | 203.81 | 1.16E+08 | 23 | Heterogeneous nuclear ribonucleoprotein K OS=Homo sapiens OX=9606 GN=HNRNPK PE=1 SV=1 |
| P06733 | 215.46 | 6.97E+07 | 22 | Alpha-enolase OS=Homo sapiens OX=9606 GN=ENO1 PE=1 SV=2 |
| P07437 | 237.17 | 4.40E+07 | 22 | Tubulin beta chain OS=Homo sapiens OX=9606 GN=TUBB PE=1 SV=2 |
| P04843 | 193.71 | 3.50E+07 | 21 | Dolichyl-diphosphooligosaccharide--protein glycosyltransferase subunit 1 OS=Homo sapiens OX=9606 GN=RPN1 PE=1 SV=1 |
| P50991 | 202.18 | 1.81E+07 | 21 | T-complex protein 1 subunit delta OS=Homo sapiens OX=9606 GN=CCT4 PE=1 SV=4 |
| P23246 | 207.36 | 4.03E+07 | 21 | Splicing factor proline- and glutamine-rich OS=Homo sapiens OX=9606 GN=SFPQ PE=1 SV=2 |
| Q16643 | 210.16 | 9.59E+07 | 21 | Drebrin OS=Homo sapiens OX=9606 GN=DBN1 PE=1 SV=4 |
| P19474 | 209.53 | 1.59E+08 | 21 | E3 ubiquitin-protein ligase TRIM21 OS=Homo sapiens OX=9606 GN=TRIM21 PE=1 SV=1 |
| P11021 | 199.86 | 2.37E+07 | 20 | Endoplasmic reticulum chaperone BiP OS=Homo sapiens OX=9606 GN=HSPA5 PE=1 SV=2 |
| P36578 | 186.08 | 3.80E+07 | 20 | 60S ribosomal protein L4 OS=Homo sapiens OX=9606 GN=RPL4 PE=1 SV=5 |
| P49411 | 209.21 | 7.19E+07 | 20 | Elongation factor Tu mitochondrial OS=Homo sapiens OX=9606 GN=TUFM PE=1 SV=2 |
| Q99959 | 171.15 | 1.68E+07 | 19 | Plakophilin-2 OS=Homo sapiens OX=9606 GN=PKP2 PE=1 SV=2 |
| P07900 | 206.57 | 6.77E+06 | 19 | Heat shock protein HSP 90-alpha OS=Homo sapiens OX=9606 GN=HSP90AA1 PE=1 SV=5 |
| Q9ULV4 | 189.66 | 5.13E+07 | 19 | Coronin-1C OS=Homo sapiens OX=9606 GN=CORO1C PE=1 SV=1 |

| Accession | -10 lgP | Area | Peptides | Description |
|---|---|---|---|---|
| O95425 | 165.78 | 9.57E+06 | 19 | Supervillin OS=Homo sapiens OX=9606 GN=SVIL PE=1 SV=2 |
| O60716 | 184.91 | 2.19E+07 | 19 | Catenin delta-1 OS=Homo sapiens OX=9606 GN=CTNND1 PE=1 SV=1 |
| P00966 | 203.66 | 4.61E+07 | 18 | Argininosuccinate synthase OS=Homo sapiens OX=9606 GN=ASS1 PE=1 SV=2 |
| P13639 | 171.89 | 1.57E+07 | 18 | Elongation factor 2 OS=Homo sapiens OX=9606 GN=EEF2 PE=1 SV=4 |
| P41250 | 174.17 | 4.55E+07 | 18 | Glycine--tRNA ligase OS=Homo sapiens OX=9606 GN=GARS1 PE=1 SV=3 |
| Q08211 | 179.87 | 2.55E+07 | 18 | ATP-dependent RNA helicase A OS=Homo sapiens OX=9606 GN=DHX9 PE=1 SV=4 |
| P62424 | 184.5 | 3.94E+07 | 17 | 60S ribosomal protein L7a OS=Homo sapiens OX=9606 GN=RPL7A PE=1 SV=2 |
| Q96PK6 | 201.63 | 4.04E+07 | 17 | RNA-binding protein 14 OS=Homo sapiens OX=9606 GN=RBM14 PE=1 SV=2 |
| P15880 | 172.1 | 5.05E+07 | 17 | 40S ribosomal protein S2 OS=Homo sapiens OX=9606 GN=RPS2 PE=1 SV=2 |
| Q9Y6K5 | 145.85 | 1.33E+07 | 17 | 2'-5'-oligoadenylate synthase 3 OS=Homo sapiens OX=9606 GN=OAS3 PE=1 SV=3 |
| Q7Z2W4 | 194.76 | 2.69E+07 | 17 | Zinc finger CCCH-type antiviral protein 1 OS=Homo sapiens OX=9606 GN=ZC3HAV1 PE=1 SV=3 |
| P63104 | 186.93 | 5.83E+07 | 17 | 14-3-3 protein zeta/delta OS=Homo sapiens OX=9606 GN=YWHAZ PE=1 SV=1 |
| Q9C0C2 | 177.99 | 9.61E+06 | 17 | 182 kDa tankyrase-1-binding protein OS=Homo sapiens OX=9606 GN=TNKS1BP1 PE=1 SV=4 |
| P78371 | 179.69 | 1.59E+07 | 17 | T-complex protein 1 subunit beta OS=Homo sapiens OX=9606 GN=CCT2 PE=1 SV=4 |
| P39023 | 179.96 | 7.30E+07 | 17 | 60S ribosomal protein L3 OS=Homo sapiens OX=9606 GN=RPL3 PE=1 SV=2 |
| P05023 | 149.21 | 3.64E+06 | 17 | Sodium/potassium-transporting ATPase subunit alpha-1 OS=Homo sapiens OX=9606 GN=ATP1A1 PE=1 SV=1 |
| Q13885 | 224.3 | 1.65E+05 | 17 | Tubulin beta-2A chain OS=Homo sapiens OX=9606 GN=TUBB2A PE=1 SV=1 |
| A5A3E0 | 195.08 | 1.42E+05 | 17 | POTE ankyrin domain family member F OS=Homo sapiens OX=9606 GN=POTEF PE=1 SV=2 |
| P49368 | 158.21 | 8.00E+07 | 16 | T-complex protein 1 subunit gamma OS=Homo sapiens OX=9606 GN=CCT3 PE=1 SV=4 |
| P62258 | 179.59 | 2.61E+07 | 16 | 14-3-3 protein epsilon OS=Homo sapiens OX=9606 GN=YWHAE PE=1 SV=1 |
| P27635 | 185.06 | 3.46E+07 | 16 | 60S ribosomal protein L10 OS=Homo sapiens OX=9606 GN=RPL10 PE=1 SV=4 |

| Accession | -10 lgP | Area | Peptides | Description |
|---|---|---|---|---|
| Q6NYC8 | 151.41 | 1.99E+07 | 16 | Phostensin OS=Homo sapiens OX=9606 GN=PPP1R18 PE=1 SV=1 |
| Q9NVI7 | 140.95 | 7.55E+06 | 16 | ATPase family AAA domain-containing protein 3A OS=Homo sapiens OX=9606 GN=ATAD3A PE=1 SV=2 |
| Q9Y446 | 158.01 | 1.86E+07 | 16 | Plakophilin-3 OS=Homo sapiens OX=9606 GN=PKP3 PE=1 SV=1 |
| P09914 | 187.96 | 1.83E+07 | 16 | Interferon-induced protein with tetratricopeptide repeats 1 OS=Homo sapiens OX=9606 GN=IFIT1 PE=1 SV=2 |
| O00571 | 193.57 | 1.78E+07 | 16 | ATP-dependent RNA helicase DDX3X OS=Homo sapiens OX=9606 GN=DDX3X PE=1 SV=3 |
| Q9BY89 | 153.88 | 6.69E+06 | 16 | Uncharacterized protein KIAA1671 OS=Homo sapiens OX=9606 GN=KIAA1671 PE=1 SV=2 |
| Q96HS1 | 204.66 | 5.54E+07 | 16 | Serine/threonine-protein phosphatase PGAM5 mitochondrial OS=Homo sapiens OX=9606 GN=PGAM5 PE=1 SV=2 |
| P0CG38 | 176.9 | 4.35E+04 | 16 | POTE ankyrin domain family member I OS=Homo sapiens OX=9606 GN=POTEI PE=3 SV=1 |
| P51114 | 161.1 | 9.37E+06 | 15 | Fragile X mental retardation syndrome-related protein 1 OS=Homo sapiens OX=9606 GN=FXR1 PE=1 SV=3 |
| Q9Y265 | 190.72 | 1.25E+07 | 15 | RuvB-like 1 OS=Homo sapiens OX=9606 GN=RUVBL1 PE=1 SV=1 |
| P09651 | 176.2 | 1.72E+07 | 15 | Heterogeneous nuclear ribonucleoprotein A1 OS=Homo sapiens OX=9606 GN=HNRNPA1 PE=1 SV=5 |
| Q99832 | 151.43 | 1.67E+07 | 15 | T-complex protein 1 subunit eta OS=Homo sapiens OX=9606 GN=CCT7 PE=1 SV=2 |
| P17858 | 177.24 | 4.13E+06 | 15 | ATP-dependent 6-phosphofructokinase liver type OS=Homo sapiens OX=9606 GN=PFKL PE=1 SV=6 |
| P62701 | 150.09 | 3.17E+06 | 15 | 40S ribosomal protein S4 X isoform OS=Homo sapiens OX=9606 GN=RPS4X PE=1 SV=2 |
| Q9P2E9 | 165.2 | 1.41E+07 | 15 | Ribosome-binding protein 1 OS=Homo sapiens OX=9606 GN=RRBP1 PE=1 SV=5 |
| P11586 | 137.91 | 3.07E+06 | 14 | C-1-tetrahydrofolate synthase cytoplasmic OS=Homo sapiens OX=9606 GN=MTHFD1 PE=1 SV=4 |
| O15231 | 142.06 | 1.39E+07 | 14 | Zinc finger protein 185 OS=Homo sapiens OX=9606 GN=ZNF185 PE=1 SV=3 |
| P61981 | 139.92 | 7.89E+06 | 14 | 14-3-3 protein gamma OS=Homo sapiens OX=9606 GN=YWHAG PE=1 SV=2 |
| P52597 | 186.42 | 2.55E+07 | 14 | Heterogeneous nuclear ribonucleoprotein F OS=Homo sapiens OX=9606 GN=HNRNPF PE=1 SV=3 |
| P53621 | 134.19 | 2.27E+06 | 14 | Coatomer subunit alpha OS=Homo sapiens OX=9606 GN=COPA PE=1 SV=2 |
| P40227 | 159.3 | 1.46E+07 | 14 | T-complex protein 1 subunit zeta OS=Homo sapiens OX=9606 GN=CCT6A PE=1 SV=3 |

| Accession | -10 lgP | Area | Peptides | Description |
|---|---|---|---|---|
| Q01813 | 166.61 | 6.95E+06 | 14 | ATP-dependent 6-phosphofructokinase platelet type OS=Homo sapiens OX=9606 GN=PFKP PE=1 SV=2 |
| Q9Y4K1 | 166.64 | 7.72E+06 | 14 | Beta/gamma crystallin domain-containing protein 1 OS=Homo sapiens OX=9606 GN=CRYBG1 PE=1 SV=3 |
| O15020 | 121.34 | 2.57E+06 | 14 | Spectrin beta chain non-erythrocytic 2 OS=Homo sapiens OX=9606 GN=SPTBN2 PE=1 SV=3 |
| P07195 | 176.23 | 1.67E+07 | 14 | L-lactate dehydrogenase B chain OS=Homo sapiens OX=9606 GN=LDHB PE=1 SV=2 |
| P16144 | 170.78 | 5.92E+06 | 14 | Integrin beta-4 OS=Homo sapiens OX=9606 GN=ITGB4 PE=1 SV=5 |
| Q5T6F2 | 185.96 | 2.45E+07 | 13 | Ubiquitin-associated protein 2 OS=Homo sapiens OX=9606 GN=UBAP2 PE=1 SV=1 |
| P35637 | 178.63 | 6.26E+07 | 13 | RNA-binding protein FUS OS=Homo sapiens OX=9606 GN=FUS PE=1 SV=1 |
| Q14315 | 160.42 | 3.54E+06 | 13 | Filamin-C OS=Homo sapiens OX=9606 GN=FLNC PE=1 SV=3 |
| P62269 | 139.57 | 4.15E+07 | 13 | 40S ribosomal protein S18 OS=Homo sapiens OX=9606 GN=RPS18 PE=1 SV=3 |
| P12268 | 176.41 | 1.19E+07 | 13 | Inosine-5'-monophosphate dehydrogenase 2 OS=Homo sapiens OX=9606 GN=IMPDH2 PE=1 SV=2 |
| Q9BUF5 | 177.92 | 2.02E+06 | 13 | Tubulin beta-6 chain OS=Homo sapiens OX=9606 GN=TUBB6 PE=1 SV=1 |
| P62280 | 152.49 | 3.49E+07 | 13 | 40S ribosomal protein S11 OS=Homo sapiens OX=9606 GN=RPS11 PE=1 SV=3 |
| Q9Y5A9 | 128.36 | 2.25E+07 | 13 | YTH domain-containing family protein 2 OS=Homo sapiens OX=9606 GN=YTHDF2 PE=1 SV=2 |
| P61160 | 155.19 | 1.65E+07 | 13 | Actin-related protein 2 OS=Homo sapiens OX=9606 GN=ACTR2 PE=1 SV=1 |
| Q13751 | 166.44 | 7.82E+06 | 13 | Laminin subunit beta-3 OS=Homo sapiens OX=9606 GN=LAMB3 PE=1 SV=1 |
| P16615 | 152.29 | 4.88E+06 | 13 | Sarcoplasmic/endoplasmic reticulum calcium ATPase 2 OS=Homo sapiens OX=9606 GN=ATP2A2 PE=1 SV=1 |
| Q02878 | 171.62 | 4.19E+07 | 12 | 60S ribosomal protein L6 OS=Homo sapiens OX=9606 GN=RPL6 PE=1 SV=3 |
| Q9Y230 | 159.3 | 1.27E+07 | 12 | RuvB-like 2 OS=Homo sapiens OX=9606 GN=RUVBL2 PE=1 SV=3 |
| Q14126 | 166.11 | 7.62E+06 | 12 | Desmoglein-2 OS=Homo sapiens OX=9606 GN=DSG2 PE=1 SV=2 |
| O60506 | 152.51 | 5.09E+06 | 12 | Heterogeneous nuclear ribonucleoprotein Q OS=Homo sapiens OX=9606 GN=SYNCRIP PE=1 SV=2 |
| O43852 | 146.01 | 6.10E+07 | 12 | Calumenin OS=Homo sapiens OX=9606 GN=CALU PE=1 SV=2 |

| Accession | -10 lgP | Area | Peptides | Description |
|---|---|---|---|---|
| P61247 | 173.34 | 3.94E+07 | 12 | 40S ribosomal protein S3a OS=Homo sapiens OX=9606 GN=RPS3A PE=1 SV=2 |
| Q9UPQ0 | 168.56 | 6.82E+06 | 12 | LIM and calponin homology domains-containing protein 1 OS=Homo sapiens OX=9606 GN=LIMCH1 PE=1 SV=4 |
| Q13310 | 173.38 | 2.76E+06 | 12 | Polyadenylate-binding protein 4 OS=Homo sapiens OX=9606 GN=PABPC4 PE=1 SV=1 |
| Q32P51 | 148.66 | 2.62E+05 | 12 | Heterogeneous nuclear ribonucleoprotein A1-like 2 OS=Homo sapiens OX=9606 GN=HNRNPA1L2 PE=2 SV=2 |
| P22695 | 159.78 | 1.29E+07 | 11 | Cytochrome b-c1 complex subunit 2 mitochondrial OS=Homo sapiens OX=9606 GN=UQCRC2 PE=1 SV=3 |
| P31946 | 161.01 | 6.44E+06 | 11 | 14-3-3 protein beta/alpha OS=Homo sapiens OX=9606 GN=YWHAB PE=1 SV=3 |
| P00338 | 144.34 | 5.86E+06 | 11 | L-lactate dehydrogenase A chain OS=Homo sapiens OX=9606 GN=LDHA PE=1 SV=2 |
| P50570 | 130.83 | 8.14E+06 | 11 | Dynamin-2 OS=Homo sapiens OX=9606 GN=DNM2 PE=1 SV=2 |
| P41252 | 151.64 | 4.42E+06 | 11 | Isoleucine--tRNA ligase cytoplasmic OS=Homo sapiens OX=9606 GN=IARS1 PE=1 SV=2 |
| P61158 | 167.98 | 1.09E+07 | 11 | Actin-related protein 3 OS=Homo sapiens OX=9606 GN=ACTR3 PE=1 SV=3 |
| P10809 | 173.36 | 1.02E+07 | 11 | 60 kDa heat shock protein mitochondrial OS=Homo sapiens OX=9606 GN=HSPD1 PE=1 SV=2 |
| P05141 | 117.05 | 8.03E+06 | 11 | ADP/ATP translocase 2 OS=Homo sapiens OX=9606 GN=SLC25A5 PE=1 SV=7 |
| P62917 | 143.73 | 4.25E+07 | 11 | 60S ribosomal protein L8 OS=Homo sapiens OX=9606 GN=RPL8 PE=1 SV=2 |
| P31040 | 148.49 | 9.54E+06 | 11 | Succinate dehydrogenase [ubiquinone] flavoprotein subunit mitochondrial OS=Homo sapiens OX=9606 GN=SDHA PE=1 SV=2 |
| P48634 | 122.27 | 3.96E+06 | 11 | Protein PRRC2A OS=Homo sapiens OX=9606 GN=PRRC2A PE=1 SV=3 |
| P07814 | 148.2 | 6.46E+06 | 11 | Bifunctional glutamate/proline--tRNA ligase OS=Homo sapiens OX=9606 GN=EPRS1 PE=1 SV=5 |
| P47755 | 148.63 | 6.42E+06 | 11 | F-actin-capping protein subunit alpha-2 OS=Homo sapiens OX=9606 GN=CAPZA2 PE=1 SV=3 |
| P67809 | 148.22 | 2.10E+07 | 11 | Y-box-binding protein 1 OS=Homo sapiens OX=9606 GN=YBX1 PE=1 SV=3 |
| P12236 | 117.07 | 3.17E+06 | 11 | ADP/ATP translocase 3 OS=Homo sapiens OX=9606 GN=SLC25A6 PE=1 SV=4 |
| Q96SB3 | 130.83 | 6.57E+06 | 10 | Neurabin-2 OS=Homo sapiens OX=9606 GN=PPP1R9B PE=1 SV=3 |

| Accession | -10 lgP | Area | Peptides | Description |
|---|---|---|---|---|
| P63244 | 175.8 | 1.42E+07 | 10 | Receptor of activated protein C kinase 1 OS=Homo sapiens OX=9606 GN=RACK1 PE=1 SV=3 |
| Q01085 | 165 | 2.00E+07 | 10 | Nucleolysin TIAR OS=Homo sapiens OX=9606 GN=TIAL1 PE=1 SV=1 |
| Q00325 | 115.56 | 4.60E+07 | 10 | Phosphate carrier protein mitochondrial OS=Homo sapiens OX=9606 GN=SLC25A3 PE=1 SV=2 |
| Q07666 | 163.01 | 9.50E+07 | 10 | KH domain-containing RNA-binding signal transduction-associated protein 1 OS=Homo sapiens OX=9606 GN=KHDRBS1 PE=1 SV=1 |
| Q9Y3I0 | 165.38 | 7.20E+06 | 10 | RNA-splicing ligase RtcB homolog OS=Homo sapiens OX=9606 GN=RTCB PE=1 SV=1 |
| Q92499 | 127.19 | 8.36E+06 | 10 | ATP-dependent RNA helicase DDX1 OS=Homo sapiens OX=9606 GN=DDX1 PE=1 SV=2 |
| P21980 | 135.72 | 2.53E+06 | 10 | Protein-glutamine gamma-glutamyltransferase 2 OS=Homo sapiens OX=9606 GN=TGM2 PE=1 SV=2 |
| P49591 | 150.91 | 7.00E+06 | 10 | Serine--tRNA ligase cytoplasmic OS=Homo sapiens OX=9606 GN=SARS1 PE=1 SV=3 |
| P13489 | 138.18 | 1.09E+07 | 10 | Ribonuclease inhibitor OS=Homo sapiens OX=9606 GN=RNH1 PE=1 SV=2 |
| P06748 | 159.49 | 5.09E+07 | 10 | Nucleophosmin OS=Homo sapiens OX=9606 GN=NPM1 PE=1 SV=2 |
| P28288 | 153.58 | 2.73E+06 | 10 | ATP-binding cassette sub-family D member 3 OS=Homo sapiens OX=9606 GN=ABCD3 PE=1 SV=1 |
| O43390 | 142.33 | 1.34E+06 | 10 | Heterogeneous nuclear ribonucleoprotein R OS=Homo sapiens OX=9606 GN=HNRNPR PE=1 SV=1 |
| Q8WZ42 | 69.46 |  | 10 | Titin OS=Homo sapiens OX=9606 GN=TTN PE=1 SV=4 |
| P16403 | 163.59 | 5.86E+07 | 9 | Histone H1.2 OS=Homo sapiens OX=9606 GN=H1-2 PE=1 SV=2 |
| P14136 | 125.84 | 3.06E+06 | 9 | Glial fibrillary acidic protein OS=Homo sapiens OX=9606 GN=GFAP PE=1 SV=1 |
| Q92598 | 127.97 | 1.64E+06 | 9 | Heat shock protein 105 kDa OS=Homo sapiens OX=9606 GN=HSPH1 PE=1 SV=1 |
| Q9UN86 | 148.19 | 1.66E+07 | 9 | Ras GTPase-activating protein-binding protein 2 OS=Homo sapiens OX=9606 GN=G3BP2 PE=1 SV=2 |
| Q14444 | 152.55 | 1.74E+07 | 9 | Caprin-1 OS=Homo sapiens OX=9606 GN=CAPRIN1 PE=1 SV=2 |
| Q9UDY2 | 107.24 | 6.25E+06 | 9 | Tight junction protein ZO-2 OS=Homo sapiens OX=9606 GN=TJP2 PE=1 SV=2 |
| O95487 | 147.51 | 1.67E+07 | 9 | Protein transport protein Sec24B OS=Homo sapiens OX=9606 GN=SEC24B PE=1 SV=2 |
| Q9UM54 | 142.16 | 6.25E+06 | 9 | Unconventional myosin-VI OS=Homo sapiens OX=9606 GN=MYO6 PE=1 SV=4 |

| Accession | -10 lgP | Area | Peptides | Description |
|---|---|---|---|---|
| Q04917 | 126.53 | 5.00E+06 | 9 | 14-3-3 protein eta OS=Homo sapiens OX=9606 GN=YWHAH PE=1 SV=4 |
| P14868 | 105.9 | 6.09E+06 | 9 | Aspartate--tRNA ligase cytoplasmic OS=Homo sapiens OX=9606 GN=DARS1 PE=1 SV=2 |
| Q8NC51 | 148.29 | 1.52E+07 | 9 | Plasminogen activator inhibitor 1 RNA-binding protein OS=Homo sapiens OX=9606 GN=SERBP1 PE=1 SV=2 |
| Q14697 | 107.65 | 4.50E+06 | 9 | Neutral alpha-glucosidase AB OS=Homo sapiens OX=9606 GN=GANAB PE=1 SV=3 |
| P14866 | 134.1 | 1.23E+07 | 9 | Heterogeneous nuclear ribonucleoprotein L OS=Homo sapiens OX=9606 GN=HNRNPL PE=1 SV=2 |
| P26599 | 152.21 | 1.69E+07 | 9 | Polypyrimidine tract-binding protein 1 OS=Homo sapiens OX=9606 GN=PTBP1 PE=1 SV=1 |
| P36542 | 122.02 | 1.57E+07 | 9 | ATP synthase subunit gamma mitochondrial OS=Homo sapiens OX=9606 GN=ATP5F1C PE=1 SV=1 |
| P54136 | 67.09 | 6.91E+06 | 9 | Arginine--tRNA ligase cytoplasmic OS=Homo sapiens OX=9606 GN=RARS1 PE=1 SV=2 |
| P11277 | 105.84 | 2.26E+04 | 9 | Spectrin beta chain erythrocytic OS=Homo sapiens OX=9606 GN=SPTB PE=1 SV=5 |
| P12235 | 104 | 9.92E+05 | 9 | ADP/ATP translocase 1 OS=Homo sapiens OX=9606 GN=SLC25A4 PE=1 SV=4 |
| Q8NF91 | 58.5 | 2.11E+05 | 9 | Nesprin-1 OS=Homo sapiens OX=9606 GN=SYNE1 PE=1 SV=4 |
| P62753 | 143.27 | 1.93E+07 | 8 | 40S ribosomal protein S6 OS=Homo sapiens OX=9606 GN=RPS6 PE=1 SV=1 |
| Q9Y285 | 117.29 | 4.54E+06 | 8 | Phenylalanine--tRNA ligase alpha subunit OS=Homo sapiens OX=9606 GN=FARSA PE=1 SV=3 |
| P67775 | 135.08 | 4.80E+06 | 8 | Serine/threonine-protein phosphatase 2A catalytic subunit alpha isoform OS=Homo sapiens OX=9606 GN=PPP2CA PE=1 SV=1 |
| P26373 | 110 | 4.33E+07 | 8 | 60S ribosomal protein L13 OS=Homo sapiens OX=9606 GN=RPL13 PE=1 SV=4 |
| P62263 | 163.19 | 2.80E+07 | 8 | 40S ribosomal protein S14 OS=Homo sapiens OX=9606 GN=RPS14 PE=1 SV=3 |
| O95573 | 113.42 | 5.15E+06 | 8 | Fatty acid CoA ligase Acsl3 OS=Homo sapiens OX=9606 GN=ACSL3 PE=1 SV=3 |
| Q9NR30 | 100.84 | 1.79E+06 | 8 | Nucleolar RNA helicase 2 OS=Homo sapiens OX=9606 GN=DDX21 PE=1 SV=5 |
| P61106 | 128.11 | 4.84E+06 | 8 | Ras-related protein Rab-14 OS=Homo sapiens OX=9606 GN=RAB14 PE=1 SV=4 |
| Q16658 | 133.97 | 5.98E+06 | 8 | Fascin OS=Homo sapiens OX=9606 GN=FSCN1 PE=1 SV=3 |
| P51991 | 115.33 | 9.19E+06 | 8 | Heterogeneous nuclear ribonucleoprotein A3 OS=Homo sapiens OX=9606 GN=HNRNPA3 PE=1 SV=2 |

| Accession | -10 lgP | Area | Peptides | Description |
|---|---|---|---|---|
| Q9NX63 | 124.81 | 2.06E+07 | 8 | MICOS complex subunit MIC19 OS=Homo sapiens OX=9606 GN=CHCHD3 PE=1 SV=1 |
| O00159 | 103.23 | 5.98E+06 | 8 | Unconventional myosin-Ic OS=Homo sapiens OX=9606 GN=MYO1C PE=1 SV=4 |
| Q9NZB2 | 134.75 | 2.57E+06 | 8 | Constitutive coactivator of PPAR-gamma-like protein 1 OS=Homo sapiens OX=9606 GN=FAM120A PE=1 SV=2 |
| Q9Y2W1 | 127.36 | 7.49E+06 | 8 | Thyroid hormone receptor-associated protein 3 OS=Homo sapiens OX=9606 GN=THRAP3 PE=1 SV=2 |
| O75694 | 88.44 | 2.25E+06 | 8 | Nuclear pore complex protein Nup155 OS=Homo sapiens OX=9606 GN=NUP155 PE=1 SV=1 |
| O14639 | 150.29 | 5.36E+06 | 8 | Actin-binding LIM protein 1 OS=Homo sapiens OX=9606 GN=ABLIM1 PE=1 SV=3 |
| P08237 | 102.47 | 6.30E+05 | 8 | ATP-dependent 6-phosphofructokinase muscle type OS=Homo sapiens OX=9606 GN=PFKM PE=1 SV=2 |
| Q13492 | 127.02 | 5.71E+06 | 7 | Phosphatidylinositol-binding clathrin assembly protein OS=Homo sapiens OX=9606 GN=PICALM PE=1 SV=2 |
| P61254 | 109.35 | 2.18E+07 | 7 | 60S ribosomal protein L26 OS=Homo sapiens OX=9606 GN=RPL26 PE=1 SV=1 |
| P31483 | 126.7 | 5.06E+06 | 7 | Nucleolysin TIA-1 isoform p40 OS=Homo sapiens OX=9606 GN=TIA1 PE=1 SV=3 |
| O75874 | 126.64 | 7.84E+06 | 7 | Isocitrate dehydrogenase [NADP] cytoplasmic OS=Homo sapiens OX=9606 GN=IDH1 PE=1 SV=2 |
| Q02978 | 98.97 | 9.12E+06 | 7 | Mitochondrial 2-oxoglutarate/malate carrier protein OS=Homo sapiens OX=9606 GN=SLC25A11 PE=1 SV=3 |
| O43684 | 137.04 | 6.31E+06 | 7 | Mitotic checkpoint protein BUB3 OS=Homo sapiens OX=9606 GN=BUB3 PE=1 SV=1 |
| Q07955 | 121.33 | 5.55E+06 | 7 | Serine/arginine-rich splicing factor 1 OS=Homo sapiens OX=9606 GN=SRSF1 PE=1 SV=2 |
| P62277 | 101.79 | 9.66E+06 | 7 | 40S ribosomal protein S13 OS=Homo sapiens OX=9606 GN=RPS13 PE=1 SV=2 |
| P07910 | 121.91 | 7.46E+06 | 7 | Heterogeneous nuclear ribonucleoproteins C1/C2 OS=Homo sapiens OX=9606 GN=HNRNPC PE=1 SV=4 |
| P53007 | 117.46 | 7.72E+06 | 7 | Tricarboxylate transport protein mitochondrial OS=Homo sapiens OX=9606 GN=SLC25A1 PE=1 SV=2 |
| O00299 | 137.8 | 4.44E+06 | 7 | Chloride intracellular channel protein 1 OS=Homo sapiens OX=9606 GN=CLIC1 PE=1 SV=4 |
| Q13637 | 127.45 | 5.00E+06 | 7 | Ras-related protein Rab-32 OS=Homo sapiens OX=9606 GN=RAB32 PE=1 SV=3 |
| Q53EP0 | 120.37 | 6.62E+06 | 7 | Fibronectin type III domain-containing protein 3B OS=Homo sapiens OX=9606 GN=FNDC3B PE=1 SV=2 |

| Accession | -10lgP | Area | Peptides | Description |
|---|---|---|---|---|
| Q12906 | 107.71 | 5.99E+06 | 7 | Interleukin enhancer-binding factor 3 OS=Homo sapiens OX=9606 GN=ILF3 PE=1 SV=3 |
| Q9NZM1 | 103.44 | 2.30E+06 | 7 | Myoferlin OS=Homo sapiens OX=9606 GN=MYOF PE=1 SV=1 |
| P18583 | 94.81 | 3.87E+06 | 7 | Protein SON OS=Homo sapiens OX=9606 GN=SON PE=1 SV=4 |
| O15427 | 111.78 | 1.40E+07 | 7 | Monocarboxylate transporter 4 OS=Homo sapiens OX=9606 GN=SLC16A3 PE=1 SV=1 |
| Q8TDB6 | 105.47 | 2.82E+06 | 7 | E3 ubiquitin-protein ligase DTX3L OS=Homo sapiens OX=9606 GN=DTX3L PE=1 SV=1 |
| P16989 | 117.7 | 3.50E+05 | 7 | Y-box-binding protein 3 OS=Homo sapiens OX=9606 GN=YBX3 PE=1 SV=4 |
| Q9Y512 | 99.74 | 5.83E+06 | 7 | Sorting and assembly machinery component 50 homolog OS=Homo sapiens OX=9606 GN=SAMM50 PE=1 SV=3 |
| Q13435 | 87.55 | 3.36E+06 | 7 | Splicing factor 3B subunit 2 OS=Homo sapiens OX=9606 GN=SF3B2 PE=1 SV=2 |
| Q9NR12 | 94.64 | 6.53E+06 | 6 | PDZ and LIM domain protein 7 OS=Homo sapiens OX=9606 GN=PDLIM7 PE=1 SV=1 |
| P16401 | 129.03 | 1.37E+07 | 6 | Histone H1.5 OS=Homo sapiens OX=9606 GN=H1-5 PE=1 SV=3 |
| P13010 | 85.67 | 9.41E+05 | 6 | X-ray repair cross-complementing protein 5 OS=Homo sapiens OX=9606 GN=XRCC5 PE=1 SV=3 |
| Q99700 | 96.22 | 2.62E+06 | 6 | Ataxin-2 OS=Homo sapiens OX=9606 GN=ATXN2 PE=1 SV=2 |
| P08708 | 101.39 | 5.53E+06 | 6 | 40S ribosomal protein S17 OS=Homo sapiens OX=9606 GN=RPS17 PE=1 SV=2 |
| Q9UJS0 | 78.68 | 2.04E+06 | 6 | Calcium-binding mitochondrial carrier protein Aralar2 OS=Homo sapiens OX=9606 GN=SLC25A13 PE=1 SV=2 |
| P62899 | 97.54 | 2.35E+07 | 6 | 60S ribosomal protein L31 OS=Homo sapiens OX=9606 GN=RPL31 PE=1 SV=1 |
| P62136 | 101.59 | 2.81E+06 | 6 | Serine/threonine-protein phosphatase PP1-alpha catalytic subunit OS=Homo sapiens OX=9606 GN=PPP1CA PE=1 SV=1 |
| O15144 | 114.4 | 8.19E+06 | 6 | Actin-related protein 2/3 complex subunit 2 OS=Homo sapiens OX=9606 GN=ARPC2 PE=1 SV=1 |
| O43175 | 125.45 | 4.02E+06 | 6 | D-3-phosphoglycerate dehydrogenase OS=Homo sapiens OX=9606 GN=PHGDH PE=1 SV=4 |
| P45880 | 129.52 | 4.10E+06 | 6 | Voltage-dependent anion-selective channel protein 2 OS=Homo sapiens OX=9606 GN=VDAC2 PE=1 SV=2 |
| Q9NSE4 | 103.53 | 1.35E+06 | 6 | Isoleucine--tRNA ligase mitochondrial OS=Homo sapiens OX=9606 GN=IARS2 PE=1 SV=2 |

| Accession | -10lgP | Area | Peptides | Description |
|---|---|---|---|---|
| P61313 | 127.51 | 2.33E+07 | 6 | 60S ribosomal protein L15 OS=Homo sapiens OX=9606 GN=RPL15 PE=1 SV=2 |
| Q5JSZ5 | 81.41 | 2.47E+06 | 6 | Protein PRRC2B OS=Homo sapiens OX=9606 GN=PRRC2B PE=1 SV=2 |
| Q06830 | 103.65 | 5.96E+06 | 6 | Peroxiredoxin-1 OS=Homo sapiens OX=9606 GN=PRDX1 PE=1 SV=1 |
| Q08J23 | 95.11 | 2.29E+06 | 6 | RNA cytosine C (5)-methyltransferase NSUN2 OS=Homo sapiens OX=9606 GN=NSUN2 PE=1 SV=2 |
| P20700 | 115.8 | 1.21E+06 | 6 | Lamin-B1 OS=Homo sapiens OX=9606 GN=LMNB1 PE=1 SV=2 |
| P62140 | 106.73 | 3.50E+06 | 6 | Serine/threonine-protein phosphatase PP1-beta catalytic subunit OS=Homo sapiens OX=9606 GN=PPP1CB PE=1 SV=3 |
| Q9Y520 | 78.87 | 2.13E+06 | 6 | Protein PRRC2C OS=Homo sapiens OX=9606 GN=PRRC2C PE=1 SV=4 |
| P33993 | 103.08 | 2.17E+06 | 6 | DNA replication licensing factor MCM7 OS=Homo sapiens OX=9606 GN=MCM7 PE=1 SV=4 |
| P46940 | 103.4 | 1.48E+06 | 6 | Ras GTPase-activating-like protein IQGAP1 OS=Homo sapiens OX=9606 GN=IQGAP1 PE=1 SV=1 |
| P43243 | 87.01 | 7.36E+06 | 6 | Matrin-3 OS=Homo sapiens OX=9606 GN=MATR3 PE=1 SV=2 |
| P35222 | 131.75 | 1.72E+06 | 6 | Catenin beta-1 OS=Homo sapiens OX=9606 GN=CTNNB1 PE=1 SV=1 |
| Q96CS3 | 127.92 | 1.30E+06 | 6 | FAS-associated factor 2 OS=Homo sapiens OX=9606 GN=FAF2 PE=1 SV=2 |
| P14625 | 114.55 | 2.09E+06 | 6 | Endoplasmin OS=Homo sapiens OX=9606 GN=HSP90B1 PE=1 SV=1 |
| Q92804 | 112.83 | 7.55E+05 | 6 | TATA-binding protein-associated factor 2N OS=Homo sapiens OX=9606 GN=TAF15 PE=1 SV=1 |
| O95757 | 108.62 | 4.70E+04 | 6 | Heat shock 70 kDa protein 4L OS=Homo sapiens OX=9606 GN=HSPA4L PE=1 SV=3 |
| P46063 | 107.06 | 4.51E+05 | 6 | ATP-dependent DNA helicase Q1 OS=Homo sapiens OX=9606 GN=RECQL PE=1 SV=3 |
| Q92928 | 99.51 | 1.63E+06 | 6 | Putative Ras-related protein Rab-1C OS=Homo sapiens OX=9606 GN=RAB1C PE=5 SV=2 |
| O43776 | 96.94 | 3.09E+06 | 6 | Asparagine--tRNA ligase cytoplasmic OS=Homo sapiens OX=9606 GN=NARS1 PE=1 SV=1 |
| P62879 | 76.66 | 4.51E+05 | 6 | Guanine nucleotide-binding protein G(I)/G(S)/G(T) subunit beta-2 OS=Homo sapiens OX=9606 GN=GNB2 PE=1 SV=3 |
| Q14160 | 72.79 | 1.32E+06 | 6 | Protein scribble homolog OS=Homo sapiens OX=9606 GN=SCRIB PE=1 SV=4 |

| Accession | -10lgP | Area | Peptides | Description |
|---|---|---|---|---|
| Q8IZT6 | 60.49 | 2.49E+06 | 6 | Abnormal spindle-like microcephaly-associated protein OS=Homo sapiens OX=9606 GN=ASPM PE=1 SV=2 |
| Q9BXP5 | 64.81 | 8.24E+05 | 5 | Serrate RNA effector molecule homolog OS=Homo sapiens OX=9606 GN=SRRT PE=1 SV=1 |
| Q9Y281 | 96.3 | 9.21E+05 | 5 | Cofilin-2 OS=Homo sapiens OX=9606 GN=CFL2 PE=1 SV=1 |
| P60866 | 111.55 | 2.30E+07 | 5 | 40S ribosomal protein S20 OS=Homo sapiens OX=9606 GN=RPS20 PE=1 SV=1 |
| Q96N67 | 74.3 | 1.28E+06 | 5 | Dedicator of cytokinesis protein 7 OS=Homo sapiens OX=9606 GN=DOCK7 PE=1 SV=4 |
| P05161 | 103.49 | 5.04E+07 | 5 | Ubiquitin-like protein ISG15 OS=Homo sapiens OX=9606 GN=ISG15 PE=1 SV=5 |
| O75083 | 85.33 | 2.09E+06 | 5 | WD repeat-containing protein 1 OS=Homo sapiens OX=9606 GN=WDR1 PE=1 SV=4 |
| Q14764 | 81.46 | 4.20E+06 | 5 | Major vault protein OS=Homo sapiens OX=9606 GN=MVP PE=1 SV=4 |
| P46778 | 74.56 | 6.80E+06 | 5 | 60S ribosomal protein L21 OS=Homo sapiens OX=9606 GN=RPL21 PE=1 SV=2 |
| O14497 | 108.81 | 1.75E+06 | 5 | AT-rich interactive domain-containing protein 1A OS=Homo sapiens OX=9606 GN=ARID1A PE=1 SV=3 |
| Q9Y6N5 | 115.84 | 2.36E+06 | 5 | Sulfide:quinone oxidoreductase mitochondrial OS=Homo sapiens OX=9606 GN=SQOR PE=1 SV=1 |
| Q9NYF8 | 82.56 | 2.22E+06 | 5 | Bcl-2-associated transcription factor 1 OS=Homo sapiens OX=9606 GN=BCLAF1 PE=1 SV=2 |
| P42677 | 107.03 | 1.13E+07 | 5 | 40S ribosomal protein S27 OS=Homo sapiens OX=9606 GN=RPS27 PE=1 SV=3 |
| P61026 | 107 | 5.93E+05 | 5 | Ras-related protein Rab-10 OS=Homo sapiens OX=9606 GN=RAB10 PE=1 SV=1 |
| P15311 | 71.84 | 3.55E+06 | 5 | Ezrin OS=Homo sapiens OX=9606 GN=EZR PE=1 SV=4 |
| Q12965 | 88.44 | 1.92E+06 | 5 | Unconventional myosin-Ie OS=Homo sapiens OX=9606 GN=MYO1E PE=1 SV=2 |
| P16885 | 73.67 | 8.07E+05 | 5 | 1-phosphatidylinositol 4 5-bisphosphate phosphodiesterase gamma-2 OS=Homo sapiens OX=9606 GN=PLCG2 PE=1 SV=4 |
| P48047 | 105.58 | 4.47E+06 | 5 | ATP synthase subunit O mitochondrial OS=Homo sapiens OX=9606 GN=ATP5PO PE=1 SV=1 |
| Q13838 | 101.07 | 1.72E+06 | 5 | Spliceosome RNA helicase DDX39B OS=Homo sapiens OX=9606 GN=DDX39B PE=1 SV=1 |
| P56537 | 131.32 | 9.95E+06 | 5 | Eukaryotic translation initiation factor 6 OS=Homo sapiens OX=9606 GN=EIF6 PE=1 SV=1 |
| Q13200 | 84.56 | 1.40E+06 | 5 | 26S proteasome non-ATPase regulatory subunit 2 OS=Homo sapiens OX=9606 GN=PSMD2 PE=1 SV=3 |

| Accession | -10lgP | Area | Peptides | Description |
|---|---|---|---|---|
| O14964 | 80.27 | 4.06E+06 | 5 | Hepatocyte growth factor-regulated tyrosine kinase substrate OS=Homo sapiens OX=9606 GN=HGS PE=1 SV=1 |
| Q86VP1 | 80.33 | 2.90E+06 | 5 | Tax1-binding protein 1 OS=Homo sapiens OX=9606 GN=TAX1BP1 PE=1 SV=2 |
| P53618 | 116.62 | 3.21E+06 | 5 | Coatomer subunit beta OS=Homo sapiens OX=9606 GN=COPB1 PE=1 SV=3 |
| O00425 | 74.03 | 1.94E+06 | 5 | Insulin-like growth factor 2 mRNA-binding protein 3 OS=Homo sapiens OX=9606 GN=IGF2BP3 PE=1 SV=2 |
| Q99613 | 69.2 | 2.12E+06 | 5 | Eukaryotic translation initiation factor 3 subunit C OS=Homo sapiens OX=9606 GN=EIF3C PE=1 SV=1 |
| P23588 | 111.45 | 9.46E+06 | 5 | Eukaryotic translation initiation factor 4B OS=Homo sapiens OX=9606 GN=EIF4B PE=1 SV=2 |
| P62995 | 96.72 | 5.31E+06 | 5 | Transformer-2 protein homolog beta OS=Homo sapiens OX=9606 GN=TRA2B PE=1 SV=1 |
| O94905 | 92.53 | 4.90E+05 | 5 | Erlin-2 OS=Homo sapiens OX=9606 GN=ERLIN2 PE=1 SV=1 |
| P49792 | 87.23 | 5.12E+05 | 5 | E3 SUMO-protein ligase RanBP2 OS=Homo sapiens OX=9606 GN=RANBP2 PE=1 SV=2 |
| P62873 | 67.98 | 3.05E+05 | 5 | Guanine nucleotide-binding protein G(I)/G(S)/G(T) subunit beta-1 OS=Homo sapiens OX=9606 GN=GNB1 PE=1 SV=3 |
| O14980 | 66.58 | 1.16E+06 | 5 | Exportin-1 OS=Homo sapiens OX=9606 GN=XPO1 PE=1 SV=1 |
| Q6P2Q9 | 59.61 | 9.75E+05 | 5 | Pre-mRNA-processing-splicing factor 8 OS=Homo sapiens OX=9606 GN=PRPF8 PE=1 SV=2 |
| P78332 | 57.28 | 1.14E+05 | 5 | RNA-binding protein 6 OS=Homo sapiens OX=9606 GN=RBM6 PE=1 SV=5 |
| Q92878 | 48.45 | 4.25E+05 | 5 | DNA repair protein RAD50 OS=Homo sapiens OX=9606 GN=RAD50 PE=1 SV=1 |
| O15269 | 69.46 | 8.82E+05 | 4 | Serine palmitoyltransferase 1 OS=Homo sapiens OX=9606 GN=SPTLC1 PE=1 SV=1 |
| Q96I24 | 85.32 | 1.53E+06 | 4 | Far upstream element-binding protein 3 OS=Homo sapiens OX=9606 GN=FUBP3 PE=1 SV=2 |
| O15143 | 90.16 | 1.47E+06 | 4 | Actin-related protein 2/3 complex subunit 1B OS=Homo sapiens OX=9606 GN=ARPC1B PE=1 SV=3 |
| P62829 | 129.02 | 7.68E+06 | 4 | 60S ribosomal protein L23 OS=Homo sapiens OX=9606 GN=RPL23 PE=1 SV=1 |
| Q9UJZ1 | 90.48 | 2.05E+06 | 4 | Stomatin-like protein 2 mitochondrial OS=Homo sapiens OX=9606 GN=STOML2 PE=1 SV=1 |
| P25205 | 79.69 | 9.00E+05 | 4 | DNA replication licensing factor MCM3 OS=Homo sapiens OX=9606 GN=MCM3 PE=1 SV=3 |

| Accession | -10lgP | Area | Peptides | Description |
|---|---|---|---|---|
| P62913 | 93.74 | 2.23E+07 | 4 | 60S ribosomal protein L11 OS=Homo sapiens OX=9606 GN=RPL11 PE=1 SV=2 |
| P04792 | 127.61 | 3.52E+06 | 4 | Heat shock protein beta-1 OS=Homo sapiens OX=9606 GN=HSPB1 PE=1 SV=2 |
| P62266 | 100.14 | 1.53E+07 | 4 | 40S ribosomal protein S23 OS=Homo sapiens OX=9606 GN=RPS23 PE=1 SV=3 |
| Q14847 | 103.27 | 5.94E+06 | 4 | LIM and SH3 domain protein 1 OS=Homo sapiens OX=9606 GN=LASP1 PE=1 SV=2 |
| O00560 | 67.89 | 1.82E+06 | 4 | Syntenin-1 OS=Homo sapiens OX=9606 GN=SDCBP PE=1 SV=1 |
| Q53GQ0 | 58.66 | 5.71E+06 | 4 | Very-long-chain 3-oxoacyl-CoA reductase OS=Homo sapiens OX=9606 GN=HSD17B12 PE=1 SV=2 |
| P29317 | 122.08 | 1.51E+06 | 4 | Ephrin type-A receptor 2 OS=Homo sapiens OX=9606 GN=EPHA2 PE=1 SV=2 |
| O43615 | 67.07 | 1.61E+06 | 4 | Mitochondrial import inner membrane translocase subunit TIM44 OS=Homo sapiens OX=9606 GN=TIMM44 PE=1 SV=2 |
| P27105 | 96.57 | 1.73E+06 | 4 | Stomatin OS=Homo sapiens OX=9606 GN=STOM PE=1 SV=3 |
| P0C0S5 | 71.69 | 1.38E+06 | 4 | Histone H2A.Z OS=Homo sapiens OX=9606 GN=H2AZ1 PE=1 SV=2 |
| Q96FW1 | 95.6 | 9.95E+05 | 4 | Ubiquitin thioesterase OTUB1 OS=Homo sapiens OX=9606 GN=OTUB1 PE=1 SV=2 |
| Q9Y3A5 | 60.49 | 1.95E+06 | 4 | Ribosome maturation protein SBDS OS=Homo sapiens OX=9606 GN=SBDS PE=1 SV=4 |
| P33991 | 90.12 | 2.32E+06 | 4 | DNA replication licensing factor MCM4 OS=Homo sapiens OX=9606 GN=MCM4 PE=1 SV=5 |
| Q9Y3Z3 | 89.1 | 2.07E+06 | 4 | Deoxynucleoside triphosphate triphosphohydrolase SAMHD1 OS=Homo sapiens OX=9606 GN=SAMHD1 PE=1 SV=2 |
| Q9Y224 | 71.06 | 3.16E+06 | 4 | RNA transcription translation and transport factor protein OS=Homo sapiens OX=9606 GN=RTRAF PE=1 SV=1 |
| P50914 | 103.73 | 2.54E+07 | 4 | 60S ribosomal protein L14 OS=Homo sapiens OX=9606 GN=RPL14 PE=1 SV=4 |
| P50416 | 100.38 | 2.56E+06 | 4 | Carnitine O-palmitoyltransferase 1 liver isoform OS=Homo sapiens OX=9606 GN=CPT1A PE=1 SV=2 |
| P48729 | 93.26 | 1.68E+05 | 4 | Casein kinase I isoform alpha OS=Homo sapiens OX=9606 GN=CSNK1A1 PE=1 SV=2 |
| O75477 | 93.19 | 1.70E+05 | 4 | Erlin-1 OS=Homo sapiens OX=9606 GN=ERLIN1 PE=1 SV=2 |
| P61006 | 87.56 | 6.94E+05 | 4 | Ras-related protein Rab-8A OS=Homo sapiens OX=9606 GN=RAB8A PE=1 SV=1 |

| Accession | -10lgP | Area | Peptides | Description |
|---|---|---|---|---|
| Q86VP6 | 81.03 | 7.17E+05 | 4 | Cullin-associated NEDD8-dissociated protein 1 OS=Homo sapiens OX=9606 GN=CAND1 PE=1 SV=2 |
| Q9H3U1 | 80.9 | 2.96E+05 | 4 | Protein unc-45 homolog A OS=Homo sapiens OX=9606 GN=UNC45A PE=1 SV=1 |
| Q16576 | 75.3 | 3.39E+06 | 4 | Histone-binding protein RBBP7 OS=Homo sapiens OX=9606 GN=RBBP7 PE=1 SV=1 |
| Q13363 | 69.52 | 3.46E+05 | 4 | C-terminal-binding protein 1 OS=Homo sapiens OX=9606 GN=CTBP1 PE=1 SV=2 |
| P35221 | 65.39 | 1.66E+06 | 4 | Catenin alpha-1 OS=Homo sapiens OX=9606 GN=CTNNA1 PE=1 SV=1 |
| Q9UQ35 | 64.35 | 9.86E+05 | 4 | Serine/arginine repetitive matrix protein 2 OS=Homo sapiens OX=9606 GN=SRRM2 PE=1 SV=2 |
| Q9H845 | 61.28 | 1.80E+06 | 4 | Complex I assembly factor ACAD9 mitochondrial OS=Homo sapiens OX=9606 GN=ACAD9 PE=1 SV=1 |
| O95163 | 60.72 | 4.02E+05 | 4 | Elongator complex protein 1 OS=Homo sapiens OX=9606 GN=ELP1 PE=1 SV=3 |
| Q8WUM0 | 60.03 | 1.03E+06 | 4 | Nuclear pore complex protein Nup133 OS=Homo sapiens OX=9606 GN=NUP133 PE=1 SV=2 |
| Q13148 | 58.7 | 3.98E+06 | 4 | TAR DNA-binding protein 43 OS=Homo sapiens OX=9606 GN=TARDBP PE=1 SV=1 |
| Q7L576 | 58.09 | 6.86E+05 | 4 | Cytoplasmic FMR1-interacting protein 1 OS=Homo sapiens OX=9606 GN=CYFIP1 PE=1 SV=1 |
| Q9UII4 | 57.04 | 1.33E+05 | 4 | E3 ISG15--protein ligase HERC5 OS=Homo sapiens OX=9606 GN=HERC5 PE=1 SV=2 |
| P27824 | 56.91 | 4.73E+05 | 4 | Calnexin OS=Homo sapiens OX=9606 GN=CANX PE=1 SV=2 |
| Q5T4S7 | 56.57 | 1.03E+06 | 4 | E3 ubiquitin-protein ligase UBR4 OS=Homo sapiens OX=9606 GN=UBR4 PE=1 SV=1 |
| P55060 | 54.13 | 1.91E+05 | 4 | Exportin-2 OS=Homo sapiens OX=9606 GN=CSE1L PE=1 SV=3 |
| O60437 | 49.08 | 5.41E+05 | 4 | Periplakin OS=Homo sapiens OX=9606 GN=PPL PE=1 SV=4 |
| P47897 | 48.92 | 7.87E+05 | 4 | Glutamine--tRNA ligase OS=Homo sapiens OX=9606 GN=QARS1 PE=1 SV=1 |
| O43166 | 45.11 | 1.60E+06 | 4 | Signal-induced proliferation-associated 1-like protein 1 OS=Homo sapiens OX=9606 GN=SIPA1L1 PE=1 SV=4 |
| P54577 | 45.09 | 8.34E+04 | 4 | Tyrosine--tRNA ligase cytoplasmic OS=Homo sapiens OX=9606 GN=YARS1 PE=1 SV=4 |
| Q14789 | 38.63 | 4.56E+05 | 4 | Golgin subfamily B member 1 OS=Homo sapiens OX=9606 GN=GOLGB1 PE=1 SV=2 |
| Q7Z333 | 37.5 | 1.24E+05 | 4 | Probable helicase senataxin OS=Homo sapiens OX=9606 GN=SETX PE=1 SV=4 |

| Accession | -10lgP | Area | Peptides | Description |
|---|---|---|---|---|
| P42167 | 72.95 | 6.64E+05 | 3 | Lamina-associated polypeptide 2 isoforms beta/gamma OS=Homo sapiens OX=9606 GN=TMPO PE=1 SV=2 |
| Q99729 | 90.66 | 4.38E+05 | 3 | Heterogeneous nuclear ribonucleoprotein A/B OS=Homo sapiens OX=9606 GN=HNRNPAB PE=1 SV=2 |
| Q6NZI2 | 93.06 | 5.03E+05 | 3 | Caveolae-associated protein 1 OS=Homo sapiens OX=9606 GN=CAVIN1 PE=1 SV=1 |
| P19525 | 85 | 6.87E+05 | 3 | Interferon-induced double-stranded RNA-activated protein kinase OS=Homo sapiens OX=9606 GN=EIF2AK2 PE=1 SV=2 |
| P39748 | 63.48 | 7.41E+05 | 3 | Flap endonuclease 1 OS=Homo sapiens OX=9606 GN=FEN1 PE=1 SV=1 |
| P84090 | 91.55 | 1.33E+06 | 3 | Enhancer of rudimentary homolog OS=Homo sapiens OX=9606 GN=ERH PE=1 SV=1 |
| Q3MHD2 | 98.64 | 4.77E+06 | 3 | Protein LSM12 homolog OS=Homo sapiens OX=9606 GN=LSM12 PE=1 SV=2 |
| P46783 | 82.06 | 2.26E+06 | 3 | 40S ribosomal protein S10 OS=Homo sapiens OX=9606 GN=RPS10 PE=1 SV=1 |
| P33992 | 64.62 | 7.36E+05 | 3 | DNA replication licensing factor MCM5 OS=Homo sapiens OX=9606 GN=MCM5 PE=1 SV=5 |
| P35613 | 114.4 | 9.30E+06 | 3 | Basigin OS=Homo sapiens OX=9606 GN=BSG PE=1 SV=2 |
| Q14166 | 56.55 | 4.79E+05 | 3 | Tubulin--tyrosine ligase-like protein 12 OS=Homo sapiens OX=9606 GN=TTLL12 PE=1 SV=2 |
| P08174 | 64.59 | 2.47E+06 | 3 | Complement decay-accelerating factor OS=Homo sapiens OX=9606 GN=CD55 PE=1 SV=4 |
| P62910 | 98.55 | 1.61E+07 | 3 | 60S ribosomal protein L32 OS=Homo sapiens OX=9606 GN=RPL32 PE=1 SV=2 |
| Q8IY63 | 86.59 | 1.83E+05 | 3 | Angiomotin-like protein 1 OS=Homo sapiens OX=9606 GN=AMOTL1 PE=1 SV=1 |
| Q9NS69 | 85.51 | 1.96E+06 | 3 | Mitochondrial import receptor subunit TOM22 homolog OS=Homo sapiens OX=9606 GN=TOMM22 PE=1 SV=3 |
| P52209 | 53.74 | 1.21E+06 | 3 | 6-phosphogluconate dehydrogenase decarboxylating OS=Homo sapiens OX=9606 GN=PGD PE=1 SV=3 |
| Q5SSJ5 | 47.84 | 3.31E+06 | 3 | Heterochromatin protein 1-binding protein 3 OS=Homo sapiens OX=9606 GN=HP1BP3 PE=1 SV=1 |
| Q9UMS4 | 48.53 | 1.55E+06 | 3 | Pre-mRNA-processing factor 19 OS=Homo sapiens OX=9606 GN=PRPF19 PE=1 SV=1 |
| P20042 | 85.08 | 2.09E+06 | 3 | Eukaryotic translation initiation factor 2 subunit 2 OS=Homo sapiens OX=9606 GN=EIF2S2 PE=1 SV=2 |
| P30740 | 65.34 | 1.66E+06 | 3 | Leukocyte elastase inhibitor OS=Homo sapiens OX=9606 GN=SERPINB1 PE=1 SV=1 |
| P35268 | 74.59 | 2.51E+06 | 3 | 60S ribosomal protein L22 OS=Homo sapiens OX=9606 GN=RPL22 PE=1 SV=2 |

| Accession | -10lgP | Area | Peptides | Description |
|---|---|---|---|---|
| Q15029 | 69.25 | 5.36E+05 | 3 | 116 kDa U5 small nuclear ribonucleoprotein component OS=Homo sapiens OX=9606 GN=EFTUD2 PE=1 SV=1 |
| P05362 | 69.84 | 1.18E+06 | 3 | Intercellular adhesion molecule 1 OS=Homo sapiens OX=9606 GN=ICAM1 PE=1 SV=2 |
| Q9NSD9 | 61.13 | 1.15E+06 | 3 | Phenylalanine--tRNA ligase beta subunit OS=Homo sapiens OX=9606 GN=FARSB PE=1 SV=3 |
| Q71RC2 | 64.51 | 1.66E+06 | 3 | La-related protein 4 OS=Homo sapiens OX=9606 GN=LARP4 PE=1 SV=3 |
| P38159 | 73.05 | 4.16E+06 | 3 | RNA-binding motif protein X chromosome OS=Homo sapiens OX=9606 GN=RBMX PE=1 SV=3 |
| Q14498 | 86.67 | 1.22E+07 | 3 | RNA-binding protein 39 OS=Homo sapiens OX=9606 GN=RBM39 PE=1 SV=2 |
| Q96P70 | 100.81 | 7.69E+05 | 3 | Importin-9 OS=Homo sapiens OX=9606 GN=IPO9 PE=1 SV=3 |
| Q15185 | 98.69 | 1.08E+06 | 3 | Prostaglandin E synthase 3 OS=Homo sapiens OX=9606 GN=PTGES3 PE=1 SV=1 |
| Q6NUK1 | 94.56 | 5.59E+05 | 3 | Calcium-binding mitochondrial carrier protein SCaMC-1 OS=Homo sapiens OX=9606 GN=SLC25A24 PE=1 SV=2 |
| P42224 | 87.72 | 2.02E+05 | 3 | Signal transducer and activator of transcription 1-alpha/beta OS=Homo sapiens OX=9606 GN=STAT1 PE=1 SV=2 |
| Q9H2U1 | 84.12 | 7.57E+05 | 3 | ATP-dependent DNA/RNA helicase DHX36 OS=Homo sapiens OX=9606 GN=DHX36 PE=1 SV=2 |
| O95786 | 83.83 | 7.89E+05 | 3 | Antiviral innate immune response receptor RIG-I OS=Homo sapiens OX=9606 GN=DDX58 PE=1 SV=2 |
| P33176 | 80.04 | 8.71E+05 | 3 | Kinesin-1 heavy chain OS=Homo sapiens OX=9606 GN=KIF5B PE=1 SV=1 |
| P19623 | 76.21 | 5.27E+05 | 3 | Spermidine synthase OS=Homo sapiens OX=9606 GN=SRM PE=1 SV=1 |
| Q13242 | 75.51 | 9.79E+05 | 3 | Serine/arginine-rich splicing factor 9 OS=Homo sapiens OX=9606 GN=SRSF9 PE=1 SV=1 |
| O15145 | 75.05 | 1.73E+06 | 3 | Actin-related protein 2/3 complex subunit 3 OS=Homo sapiens OX=9606 GN=ARPC3 PE=1 SV=3 |
| P49750 | 74.06 | 1.49E+06 | 3 | YLP motif-containing protein 1 OS=Homo sapiens OX=9606 GN=YLPM1 PE=1 SV=4 |
| Q32MZ4 | 73.53 | 4.76E+05 | 3 | Leucine-rich repeat flightless-interacting protein 1 OS=Homo sapiens OX=9606 GN=LRRFIP1 PE=1 SV=2 |
| Q96RQ3 | 72.99 | 1.03E+06 | 3 | Methylcrotonoyl-CoA carboxylase subunit alpha mitochondrial OS=Homo sapiens OX=9606 GN=MCCC1 PE=1 SV=3 |
| Q8N0X7 | 72.13 | 1.07E+06 | 3 | Spartin OS=Homo sapiens OX=9606 GN=SPART PE=1 SV=1 |

| Accession | -10lgP | Area | Peptides | Description |
|---|---|---|---|---|
| Q9UQE7 | 71.35 | 4.79E+05 | 3 | Structural maintenance of chromosomes protein 3 OS=Homo sapiens OX=9606 GN=SMC3 PE=1 SV=2 |
| Q96IU4 | 71.3 | 1.27E+06 | 3 | Protein ABHD14B OS=Homo sapiens OX=9606 GN=ABHD14B PE=1 SV=1 |
| P27816 | 69.87 | 2.20E+06 | 3 | Microtubule-associated protein 4 OS=Homo sapiens OX=9606 GN=MAP4 PE=1 SV=3 |
| Q9Y2L1 | 68.63 | 3.52E+05 | 3 | Exosome complex exonuclease RRP44 OS=Homo sapiens OX=9606 GN=DIS3 PE=1 SV=2 |
| P04908 | 67.02 | 2.57E+05 | 3 | Histone H2A type 1-B/E OS=Homo sapiens OX=9606 GN=H2AC4 PE=1 SV=2 |
| Q9BR76 | 63.46 | 2.73E+05 | 3 | Coronin-1B OS=Homo sapiens OX=9606 GN=CORO1B PE=1 SV=1 |
| Q9BYK8 | 63.44 | 6.34E+05 | 3 | Helicase with zinc finger domain 2 OS=Homo sapiens OX=9606 GN=HELZ2 PE=1 SV=6 |
| P04899 | 63.32 | 2.63E+06 | 3 | Guanine nucleotide-binding protein G(i) subunit alpha-2 OS=Homo sapiens OX=9606 GN=GNAI2 PE=1 SV=3 |
| P30084 | 62.85 | 1.79E+06 | 3 | Enoyl-CoA hydratase mitochondrial OS=Homo sapiens OX=9606 GN=ECHS1 PE=1 SV=4 |
| P26639 | 60.69 | 3.33E+06 | 3 | Threonine--tRNA ligase 1 cytoplasmic OS=Homo sapiens OX=9606 GN=TARS1 PE=1 SV=3 |
| O95373 | 59.93 | 7.68E+05 | 3 | Importin-7 OS=Homo sapiens OX=9606 GN=IPO7 PE=1 SV=1 |
| P31689 | 59.58 | 8.88E+05 | 3 | DnaJ homolog subfamily A member 1 OS=Homo sapiens OX=9606 GN=DNAJA1 PE=1 SV=2 |
| Q99439 | 59.38 | 1.07E+06 | 3 | Calponin-2 OS=Homo sapiens OX=9606 GN=CNN2 PE=1 SV=4 |
| P49588 | 59.11 | 3.70E+05 | 3 | Alanine--tRNA ligase cytoplasmic OS=Homo sapiens OX=9606 GN=AARS1 PE=1 SV=2 |
| P17655 | 57.55 | 8.25E+05 | 3 | Calpain-2 catalytic subunit OS=Homo sapiens OX=9606 GN=CAPN2 PE=1 SV=6 |
| Q13595 | 57.43 | 2.52E+05 | 3 | Transformer-2 protein homolog alpha OS=Homo sapiens OX=9606 GN=TRA2A PE=1 SV=1 |
| P51532 | 56.8 | 5.57E+05 | 3 | Transcription activator BRG1 OS=Homo sapiens OX=9606 GN=SMARCA4 PE=1 SV=2 |
| P30153 | 55.27 | 2.99E+07 | 3 | Serine/threonine-protein phosphatase 2A 65 kDa regulatory subunit A alpha isoform OS=Homo sapiens OX=9606 GN=PPP2R1A PE=1 SV=4 |
| O43592 | 50.97 | 1.00E+05 | 3 | Exportin-T OS=Homo sapiens OX=9606 GN=XPOT PE=1 SV=2 |
| P63010 | 49.78 | 4.16E+05 | 3 | AP-2 complex subunit beta OS=Homo sapiens OX=9606 GN=AP2B1 PE=1 SV=1 |
| Q14974 | 48.5 | 6.10E+05 | 3 | Importin subunit beta-1 OS=Homo sapiens OX=9606 GN=KPNB1 PE=1 SV=2 |

| Accession | -10lgP | Area | Peptides | Description |
|---|---|---|---|---|
| Q7L4E1 | 48.18 | | 3 | Mitoguardin 2 OS=Homo sapiens OX=9606 GN=MIGA2 PE=1 SV=1 |
| P49674 | 46.98 | 1.08E+06 | 3 | Casein kinase I isoform epsilon OS=Homo sapiens OX=9606 GN=CSNK1E PE=1 SV=1 |
| O75436 | 46.98 | 7.33E+05 | 3 | Vacuolar protein sorting-associated protein 26A OS=Homo sapiens OX=9606 GN=VPS26A PE=1 SV=2 |
| P52948 | 46.46 | 2.03E+06 | 3 | Nuclear pore complex protein Nup98-Nup96 OS=Homo sapiens OX=9606 GN=NUP98 PE=1 SV=4 |
| O60333 | 45.3 | 2.88E+05 | 3 | Kinesin-like protein KIF1B OS=Homo sapiens OX=9606 GN=KIF1B PE=1 SV=5 |
| Q5H9R7 | 45.15 | 2.13E+05 | 3 | Serine/threonine-protein phosphatase 6 regulatory subunit 3 OS=Homo sapiens OX=9606 GN=PPP6R3 PE=1 SV=2 |
| P31930 | 42.89 | 8.26E+05 | 3 | Cytochrome b-c1 complex subunit 1 mitochondrial OS=Homo sapiens OX=9606 GN=UQCRC1 PE=1 SV=3 |
| Q6UB35 | 42.78 | 3.02E+05 | 3 | Monofunctional C1-tetrahydrofolate synthase mitochondrial OS=Homo sapiens OX=9606 GN=MTHFD1L PE=1 SV=1 |
| O75306 | 42.28 | 4.10E+05 | 3 | NADH dehydrogenase [ubiquinone] iron-sulfur protein 2 mitochondrial OS=Homo sapiens OX=9606 GN=NDUFS2 PE=1 SV=2 |
| P13693 | 42.24 | 3.43E+05 | 3 | Translationally-controlled tumor protein OS=Homo sapiens OX=9606 GN=TPT1 PE=1 SV=1 |
| Q9Y6V0 | 41.67 | 0.00E+00 | 3 | Protein piccolo OS=Homo sapiens OX=9606 GN=PCLO PE=1 SV=5 |
| Q5M775 | 40.63 | 4.62E+04 | 3 | Cytospin-B OS=Homo sapiens OX=9606 GN=SPECC1 PE=1 SV=1 |
| Q5T5P2 | 40.14 | 9.82E+05 | 3 | Sickle tail protein homolog OS=Homo sapiens OX=9606 GN=KIAA1217 PE=1 SV=2 |
| Q12769 | 39.61 | 3.31E+05 | 3 | Nuclear pore complex protein Nup160 OS=Homo sapiens OX=9606 GN=NUP160 PE=1 SV=3 |
| Q9Y2G9 | 39.29 | 2.06E+08 | 3 | Protein strawberry notch homolog 2 OS=Homo sapiens OX=9606 GN=SBNO2 PE=2 SV=3 |
| Q02809 | 39.24 | 1.21E+06 | 3 | Procollagen-lysine 2-oxoglutarate 5-dioxygenase 1 OS=Homo sapiens OX=9606 GN=PLOD1 PE=1 SV=2 |
| Q9Y2J2 | 38.56 | | 3 | Band 4.1-like protein 3 OS=Homo sapiens OX=9606 GN=EPB41L3 PE=1 SV=2 |
| O43157 | 37.69 | 5.75E+04 | 3 | Plexin-B1 OS=Homo sapiens OX=9606 GN=PLXNB1 PE=1 SV=3 |
| O00505 | 37.6 | 1.02E+06 | 3 | Importin subunit alpha-4 OS=Homo sapiens OX=9606 GN=KPNA3 PE=1 SV=2 |
| Q9BVP2 | 37.42 | 1.09E+05 | 3 | Guanine nucleotide-binding protein-like 3 OS=Homo sapiens OX=9606 GN=GNL3 PE=1 SV=2 |

| Accession | -10lgP | Area | Peptides | Description |
|---|---|---|---|---|
| Q12830 | 33.97 | 1.17E+06 | 3 | Nucleosome-remodeling factor subunit BPTF OS=Homo sapiens OX=9606 GN=BPTF PE=1 SV=3 |
| Q9Y613 | 33.32 | 5.20E+05 | 3 | FH1/FH2 domain-containing protein 1 OS=Homo sapiens OX=9606 GN=FHOD1 PE=1 SV=3 |
| P13164 | 70.88 | 8.39E+05 | 2 | Interferon-induced transmembrane protein 1 OS=Homo sapiens OX=9606 GN=IFITM1 PE=1 SV=3 |
| Q07021 | 60.8 | 6.23E+05 | 2 | Complement component 1 Q subcomponent-binding protein mitochondrial OS=Homo sapiens OX=9606 GN=C1QBP PE=1 SV=1 |
| P84103 | 41.43 | 6.19E+05 | 2 | Serine/arginine-rich splicing factor 3 OS=Homo sapiens OX=9606 GN=SRSF3 PE=1 SV=1 |
| P47914 | 88.32 | 3.11E+07 | 2 | 60S ribosomal protein L29 OS=Homo sapiens OX=9606 GN=RPL29 PE=1 SV=2 |
| P30566 | 31.87 | 1.63E+06 | 2 | Adenylosuccinate lyase OS=Homo sapiens OX=9606 GN=ADSL PE=1 SV=2 |
| Q96JB2 | 27.16 | 4.58E+05 | 2 | Conserved oligomeric Golgi complex subunit 3 OS=Homo sapiens OX=9606 GN=COG3 PE=1 SV=3 |
| Q16630 | 54.21 | 4.66E+05 | 2 | Cleavage and polyadenylation specificity factor subunit 6 OS=Homo sapiens OX=9606 GN=CPSF6 PE=1 SV=2 |
| P06703 | 43.25 | 5.48E+06 | 2 | Protein S100-A6 OS=Homo sapiens OX=9606 GN=S100A6 PE=1 SV=1 |
| Q15084 | 86.84 | 8.53E+05 | 2 | Protein disulfide-isomerase A6 OS=Homo sapiens OX=9606 GN=PDIA6 PE=1 SV=1 |
| P30536 | 54.51 | 6.79E+06 | 2 | Translocator protein OS=Homo sapiens OX=9606 GN=TSPO PE=1 SV=3 |
| Q9H4M9 | 65.06 | 8.13E+05 | 2 | EH domain-containing protein 1 OS=Homo sapiens OX=9606 GN=EHD1 PE=1 SV=2 |
| P13987 | 61.36 | 2.69E+06 | 2 | CD59 glycoprotein OS=Homo sapiens OX=9606 GN=CD59 PE=1 SV=1 |
| P63241 | 56.72 | 3.62E+06 | 2 | Eukaryotic translation initiation factor 5A-1 OS=Homo sapiens OX=9606 GN=EIF5A PE=1 SV=2 |
| P24539 | 36.25 | 3.72E+06 | 2 | ATP synthase F(0) complex subunit B1 mitochondrial OS=Homo sapiens OX=9606 GN=ATP5PB PE=1 SV=2 |
| Q9NZ01 | 53.05 | 2.92E+06 | 2 | Very-long-chain enoyl-CoA reductase OS=Homo sapiens OX=9606 GN=TECR PE=1 SV=1 |
| P62633 | 77.17 | 2.45E+06 | 2 | CCHC-type zinc finger nucleic acid binding protein OS=Homo sapiens OX=9606 GN=CNBP PE=1 SV=1 |
| Q16629 | 66.95 | 2.28E+06 | 2 | Serine/arginine-rich splicing factor 7 OS=Homo sapiens OX=9606 GN=SRSF7 PE=1 SV=1 |
| Q969Q0 | 51.82 | 1.11E+07 | 2 | 60S ribosomal protein L36a-like OS=Homo sapiens OX=9606 GN=RPL36AL PE=1 SV=3 |
| P62841 | 63.75 | 1.01E+07 | 2 | 40S ribosomal protein S15 OS=Homo sapiens OX=9606 GN=RPS15 PE=1 SV=2 |

| Accession | -10lgP | Area | Peptides | Description |
|---|---|---|---|---|
| Q01650 | 76.48 | 8.32E+05 | 2 | Large neutral amino acids transporter small subunit 1 OS=Homo sapiens OX=9606 GN=SLC7A5 PE=1 SV=2 |
| O95747 | 73.1 | 6.96E+05 | 2 | Serine/threonine-protein kinase OSR1 OS=Homo sapiens OX=9606 GN=OXSR1 PE=1 SV=1 |
| O75439 | 72.39 | 1.50E+06 | 2 | Mitochondrial-processing peptidase subunit beta OS=Homo sapiens OX=9606 GN=PMPCB PE=1 SV=2 |
| Q5K651 | 68.88 | 3.67E+05 | 2 | Sterile alpha motif domain-containing protein 9 OS=Homo sapiens OX=9606 GN=SAMD9 PE=1 SV=1 |
| Q9BPX5 | 66.51 | 1.27E+06 | 2 | Actin-related protein 2/3 complex subunit 5-like protein OS=Homo sapiens OX=9606 GN=ARPC5L PE=1 SV=1 |
| Q92616 | 63.73 | 7.13E+05 | 2 | eIF-2-alpha kinase activator GCN1 OS=Homo sapiens OX=9606 GN=GCN1 PE=1 SV=6 |
| Q96QD8 | 63.43 | 8.50E+05 | 2 | Sodium-coupled neutral amino acid transporter 2 OS=Homo sapiens OX=9606 GN=SLC38A2 PE=1 SV=2 |
| Q8NFJ5 | 62.22 | 9.22E+05 | 2 | Retinoic acid-induced protein 3 OS=Homo sapiens OX=9606 GN=GPRC5A PE=1 SV=2 |
| Q8WUF5 | 61.84 | 3.94E+05 | 2 | RelA-associated inhibitor OS=Homo sapiens OX=9606 GN=PPP1R13L PE=1 SV=4 |
| P55735 | 61.52 | 4.66E+05 | 2 | Protein SEC13 homolog OS=Homo sapiens OX=9606 GN=SEC13 PE=1 SV=3 |
| O00303 | 60.23 | 1.46E+05 | 2 | Eukaryotic translation initiation factor 3 subunit F OS=Homo sapiens OX=9606 GN=EIF3F PE=1 SV=1 |
| Q53H96 | 59.41 | 4.90E+05 | 2 | Pyrroline-5-carboxylate reductase 3 OS=Homo sapiens OX=9606 GN=PYCR3 PE=1 SV=3 |
| Q9NXW2 | 58.07 | 3.21E+05 | 2 | DnaJ homolog subfamily B member 12 OS=Homo sapiens OX=9606 GN=DNAJB12 PE=1 SV=5 |
| Q9Y2J4 | 57.42 | 4.56E+05 | 2 | Angiomotin-like protein 2 OS=Homo sapiens OX=9606 GN=AMOTL2 PE=1 SV=3 |
| Q92785 | 56.76 | 1.49E+05 | 2 | Zinc finger protein ubi-d4 OS=Homo sapiens OX=9606 GN=DPF2 PE=1 SV=2 |
| O94874 | 55.41 | 3.99E+05 | 2 | E3 UFM1-protein ligase 1 OS=Homo sapiens OX=9606 GN=UFL1 PE=1 SV=2 |
| Q13188 | 55.14 | 3.67E+05 | 2 | Serine/threonine-protein kinase 3 OS=Homo sapiens OX=9606 GN=STK3 PE=1 SV=2 |
| P15407 | 54.81 | 5.45E+06 | 2 | Fos-related antigen 1 OS=Homo sapiens OX=9606 GN=FOSL1 PE=1 SV=1 |
| Q13547 | 53.57 | 8.19E+05 | 2 | Histone deacetylase 1 OS=Homo sapiens OX=9606 GN=HDAC1 PE=1 SV=1 |
| O00410 | 52.04 | 8.94E+05 | 2 | Importin-5 OS=Homo sapiens OX=9606 GN=IPO5 PE=1 SV=4 |
| Q5JWF2 | 50.25 | 3.35E+05 | 2 | Guanine nucleotide-binding protein G(s) subunit alpha isoforms XLas OS=Homo sapiens OX=9606 GN=GNAS PE=1 SV=2 |

| Accession | -10lgP | Area | Peptides | Description |
|---|---|---|---|---|
| O15260 | 50.03 | 2.93E+05 | 2 | Surfeit locus protein 4 OS=Homo sapiens OX=9606 GN=SURF4 PE=1 SV=3 |
| Q12904 | 49.94 | 5.89E+05 | 2 | Aminoacyl tRNA synthase complex-interacting multifunctional protein 1 OS=Homo sapiens OX=9606 GN=AIMP1 PE=1 SV=2 |
| Q86YQ8 | 48.91 | 5.58E+04 | 2 | Copine-8 OS=Homo sapiens OX=9606 GN=CPNE8 PE=1 SV=2 |
| O96008 | 48 | 5.64E+05 | 2 | Mitochondrial import receptor subunit TOM40 homolog OS=Homo sapiens OX=9606 GN=TOMM40 PE=1 SV=1 |
| Q9P2R7 | 47.64 | 5.64E+05 | 2 | Succinate--CoA ligase [ADP-forming] subunit beta mitochondrial OS=Homo sapiens OX=9606 GN=SUCLA2 PE=1 SV=3 |
| P25398 | 45.65 | 2.99E+06 | 2 | 40S ribosomal protein S12 OS=Homo sapiens OX=9606 GN=RPS12 PE=1 SV=3 |
| P02786 | 44.97 | 1.09E+06 | 2 | Transferrin receptor protein 1 OS=Homo sapiens OX=9606 GN=TFRC PE=1 SV=2 |
| Q5VZF2 | 44.91 | 2.05E+05 | 2 | Muscleblind-like protein 2 OS=Homo sapiens OX=9606 GN=MBNL2 PE=1 SV=2 |
| Q16401 | 44.86 | 2.72E+05 | 2 | 26S proteasome non-ATPase regulatory subunit 5 OS=Homo sapiens OX=9606 GN=PSMD5 PE=1 SV=3 |
| P35237 | 44.59 | 2.42E+05 | 2 | Serpin B6 OS=Homo sapiens OX=9606 GN=SERPINB6 PE=1 SV=3 |
| Q13418 | 43.67 | 1.91E+06 | 2 | Integrin-linked protein kinase OS=Homo sapiens OX=9606 GN=ILK PE=1 SV=2 |
| P78406 | 43.5 | 1.53E+06 | 2 | mRNA export factor OS=Homo sapiens OX=9606 GN=RAE1 PE=1 SV=1 |
| Q16543 | 42.93 | 8.48E+05 | 2 | Hsp90 co-chaperone Cdc37 OS=Homo sapiens OX=9606 GN=CDC37 PE=1 SV=1 |
| P41743 | 41.42 | 1.86E+05 | 2 | Protein kinase C iota type OS=Homo sapiens OX=9606 GN=PRKCI PE=1 SV=2 |
| Q14244 | 41.09 | 3.77E+05 | 2 | Ensconsin OS=Homo sapiens OX=9606 GN=MAP7 PE=1 SV=1 |
| P84095 | 41.05 | 2.01E+06 | 2 | Rho-related GTP-binding protein RhoG OS=Homo sapiens OX=9606 GN=RHOG PE=1 SV=1 |
| Q6PKG0 | 40.27 | 4.21E+04 | 2 | La-related protein 1 OS=Homo sapiens OX=9606 GN=LARP1 PE=1 SV=2 |
| P84550 | 39.6 | 5.35E+05 | 2 | SKI family transcriptional corepressor 1 OS=Homo sapiens OX=9606 GN=SKOR1 PE=1 SV=1 |
| A0FGR8 | 39.07 | 6.97E+05 | 2 | Extended synaptotagmin-2 OS=Homo sapiens OX=9606 GN=ESYT2 PE=1 SV=1 |
| Q8NF37 | 38.45 | 4.65E+05 | 2 | Lysophosphatidylcholine acyltransferase 1 OS=Homo sapiens OX=9606 GN=LPCAT1 PE=1 SV=2 |

| Accession | -10lgP | Area | Peptides | Description |
|---|---|---|---|---|
| Q14203 | 38.4 | 3.28E+06 | 2 | Dynactin subunit 1 OS=Homo sapiens OX=9606 GN=DCTN1 PE=1 SV=3 |
| Q96Q11 | 38.32 | 1.43E+06 | 2 | CCA tRNA nucleotidyltransferase 1 mitochondrial OS=Homo sapiens OX=9606 GN=TRNT1 PE=1 SV=2 |
| Q9Y6G9 | 38.18 | 6.02E+05 | 2 | Cytoplasmic dynein 1 light intermediate chain 1 OS=Homo sapiens OX=9606 GN=DYNC1LI1 PE=1 SV=3 |
| Q9UNM6 | 37.9 | 2.42E+05 | 2 | 26S proteasome non-ATPase regulatory subunit 13 OS=Homo sapiens OX=9606 GN=PSMD13 PE=1 SV=2 |
| Q96KP1 | 36.96 | 1.34E+05 | 2 | Exocyst complex component 2 OS=Homo sapiens OX=9606 GN=EXOC2 PE=1 SV=1 |
| Q8NDB2 | 35.74 | 9.13E+05 | 2 | B-cell scaffold protein with ankyrin repeats OS=Homo sapiens OX=9606 GN=BANK1 PE=1 SV=3 |
| Q9UKM9 | 35.68 | 2.16E+05 | 2 | RNA-binding protein Raly OS=Homo sapiens OX=9606 GN=RALY PE=1 SV=1 |
| P62191 | 35.43 | 4.85E+05 | 2 | 26S proteasome regulatory subunit 4 OS=Homo sapiens OX=9606 GN=PSMC1 PE=1 SV=1 |
| Q9UL45 | 35.37 | 6.65E+05 | 2 | Biogenesis of lysosome-related organelles complex 1 subunit 6 OS=Homo sapiens OX=9606 GN=BLOC1S6 PE=1 SV=1 |
| O75150 | 35.18 | 5.00E+05 | 2 | E3 ubiquitin-protein ligase BRE1B OS=Homo sapiens OX=9606 GN=RNF40 PE=1 SV=5 |
| P10321 | 35.01 | 2.69E+06 | 2 | HLA class I histocompatibility antigen C alpha chain OS=Homo sapiens OX=9606 GN=HLA-C PE=1 SV=3 |
| Q9Y6D9 | 34.92 | 8.98E+05 | 2 | Mitotic spindle assembly checkpoint protein MAD1 OS=Homo sapiens OX=9606 GN=MAD1L1 PE=1 SV=2 |
| P35659 | 34.81 | 1.48E+06 | 2 | Protein DEK OS=Homo sapiens OX=9606 GN=DEK PE=1 SV=1 |
| P15170 | 34.7 | 6.07E+05 | 2 | Eukaryotic peptide chain release factor GTP-binding subunit ERF3A OS=Homo sapiens OX=9606 GN=GSPT1 PE=1 SV=1 |
| P46060 | 34.46 | 3.01E+05 | 2 | Ran GTPase-activating protein 1 OS=Homo sapiens OX=9606 GN=RANGAP1 PE=1 SV=1 |
| P23921 | 34.33 | 4.80E+05 | 2 | Ribonucleoside-diphosphate reductase large subunit OS=Homo sapiens OX=9606 GN=RRM1 PE=1 SV=1 |
| Q9H074 | 33.95 | 2.30E+05 | 2 | Polyadenylate-binding protein-interacting protein 1 OS=Homo sapiens OX=9606 GN=PAIP1 PE=1 SV=1 |
| Q9NVV4 | 33.18 | 4.95E+05 | 2 | Poly(A) RNA polymerase mitochondrial OS=Homo sapiens OX=9606 GN=MTPAP PE=1 SV=1 |
| Q9BVA0 | 29.49 | 4.06E+05 | 2 | Katanin p80 WD40 repeat-containing subunit B1 OS=Homo sapiens OX=9606 GN=KATNB1 PE=1 SV=1 |
| Q9HCE1 | 29.31 | 3.03E+05 | 2 | Helicase MOV-10 OS=Homo sapiens OX=9606 GN=MOV10 PE=1 SV=2 |

| Accession | -10lgP | Area | Peptides | Description |
|---|---|---|---|---|
| Q92482 | 29.3 | 4.36E+05 | 2 | Aquaporin-3 OS=Homo sapiens OX=9606 GN=AQP3 PE=1 SV=2 |
| Q9H9T3 | 29.17 | 3.76E+04 | 2 | Elongator complex protein 3 OS=Homo sapiens OX=9606 GN=ELP3 PE=1 SV=2 |
| Q8NE71 | 29.1 | 5.40E+05 | 2 | ATP-binding cassette sub-family F member 1 OS=Homo sapiens OX=9606 GN=ABCF1 PE=1 SV=2 |
| Q6ZNE9 | 26.6 | 8.20E+05 | 2 | RUN and FYVE domain-containing protein 4 OS=Homo sapiens OX=9606 GN=RUFY4 PE=1 SV=2 |
| Q6PIF6 | 26.22 | 2.51E+05 | 2 | Unconventional myosin-VIIb OS=Homo sapiens OX=9606 GN=MYO7B PE=1 SV=2 |
| P07305 | 22.27 | 1.24E+06 | 1 | Histone H1.0 OS=Homo sapiens OX=9606 GN=H1-0 PE=1 SV=3 |
| A0A0C4DH67 | 41 | 7.85E+07 | 1 | Immunoglobulin kappa variable 1-8 OS=Homo sapiens OX=9606 GN=IGKV1-8 PE=3 SV=1 |
| P15153 | 40.57 | 1.40E+06 | 1 | Ras-related C3 botulinum toxin substrate 2 OS=Homo sapiens OX=9606 GN=RAC2 PE=1 SV=1 |
| P62854 | 36.14 | 9.76E+06 | 1 | 40S ribosomal protein S26 OS=Homo sapiens OX=9606 GN=RPS26 PE=1 SV=3 |
| P05198 | 45.27 | 7.68E+05 | 1 | Eukaryotic translation initiation factor 2 subunit 1 OS=Homo sapiens OX=9606 GN=EIF2S1 PE=1 SV=3 |
| P62306 | 42.04 | 7.70E+05 | 1 | Small nuclear ribonucleoprotein F OS=Homo sapiens OX=9606 GN=SNRPF PE=1 SV=1 |
| Q15181 | 42.43 | 2.39E+05 | 1 | Inorganic pyrophosphatase OS=Homo sapiens OX=9606 GN=PPA1 PE=1 SV=2 |
| P63208 | 51.43 | 5.15E+05 | 1 | S-phase kinase-associated protein 1 OS=Homo sapiens OX=9606 GN=SKP1 PE=1 SV=2 |
| P35080 | 53.19 | 1.90E+06 | 1 | Profilin-2 OS=Homo sapiens OX=9606 GN=PFN2 PE=1 SV=3 |
| P61619 | 39.59 | 2.86E+06 | 1 | Protein transport protein Sec61 subunit alpha isoform 1 OS=Homo sapiens OX=9606 GN=SEC61A1 PE=1 SV=2 |
| Q9UHD1 | 68.64 | 9.36E+04 | 1 | Cysteine and histidine-rich domain-containing protein 1 OS=Homo sapiens OX=9606 GN=CHORDC1 PE=1 SV=2 |
| Q92522 | 64.54 | 4.46E+05 | 1 | Histone H1.10 OS=Homo sapiens OX=9606 GN=H1-10 PE=1 SV=1 |
| Q9H3P7 | 56 | 1.74E+05 | 1 | Golgi resident protein GCP60 OS=Homo sapiens OX=9606 GN=ACBD3 PE=1 SV=4 |
| P17275 | 54.08 | 2.13E+06 | 1 | Transcription factor jun-B OS=Homo sapiens OX=9606 GN=JUNB PE=1 SV=1 |
| P22314 | 53.67 | 3.58E+05 | 1 | Ubiquitin-like modifier-activating enzyme 1 OS=Homo sapiens OX=9606 GN=UBA1 PE=1 SV=3 |
| Q15427 | 52.85 | 1.04E+05 | 1 | Splicing factor 3B subunit 4 OS=Homo sapiens OX=9606 GN=SF3B4 PE=1 SV=1 |

| Accession | -10lgP | Area | Peptides | Description |
|---|---|---|---|---|
| P63173 | 52.67 | 3.37E+05 | 1 | 60S ribosomal protein L38 OS=Homo sapiens OX=9606 GN=RPL38 PE=1 SV=2 |
| Q9ULX6 | 51.7 | 4.13E+05 | 1 | A-kinase anchor protein 8-like OS=Homo sapiens OX=9606 GN=AKAP8L PE=1 SV=4 |
| P61019 | 50.97 | 1.03E+05 | 1 | Ras-related protein Rab-2A OS=Homo sapiens OX=9606 GN=RAB2A PE=1 SV=1 |
| Q03519 | 49.89 | 1.08E+05 | 1 | Antigen peptide transporter 2 OS=Homo sapiens OX=9606 GN=TAP2 PE=1 SV=1 |
| P17081 | 48.99 | 4.10E+05 | 1 | Rho-related GTP-binding protein RhoQ OS=Homo sapiens OX=9606 GN=RHOQ PE=1 SV=2 |
| P40616 | 48.27 | 3.40E+05 | 1 | ADP-ribosylation factor-like protein 1 OS=Homo sapiens OX=9606 GN=ARL1 PE=1 SV=1 |
| P56134 | 47.99 | 1.60E+05 | 1 | ATP synthase subunit f mitochondrial OS=Homo sapiens OX=9606 GN=ATP5MF PE=1 SV=3 |
| Q8IXQ6 | 46.42 | 1.80E+05 | 1 | Protein mono-ADP-ribosyltransferase PARP9 OS=Homo sapiens OX=9606 GN=PARP9 PE=1 SV=2 |
| P51148 | 46.25 | 7.89E+05 | 1 | Ras-related protein Rab-5C OS=Homo sapiens OX=9606 GN=RAB5C PE=1 SV=2 |
| Q9NTJ3 | 45.03 | 3.38E+05 | 1 | Structural maintenance of chromosomes protein 4 OS=Homo sapiens OX=9606 GN=SMC4 PE=1 SV=2 |
| Q9UKS6 | 43.93 | 6.22E+05 | 1 | Protein kinase C and casein kinase substrate in neurons protein 3 OS=Homo sapiens OX=9606 GN=PACSIN3 PE=1 SV=2 |
| O43572 | 42.74 | 2.09E+05 | 1 | A-kinase anchor protein 10 mitochondrial OS=Homo sapiens OX=9606 GN=AKAP10 PE=1 SV=2 |
| Q96HN2 | 42.01 | 1.63E+05 | 1 | Adenosylhomocysteinase 3 OS=Homo sapiens OX=9606 GN=AHCYL2 PE=1 SV=1 |
| O95817 | 41.41 | 1.24E+05 | 1 | BAG family molecular chaperone regulator 3 OS=Homo sapiens OX=9606 GN=BAG3 PE=1 SV=3 |
| Q9Y2A7 | 40.1 | 7.07E+04 | 1 | Nck-associated protein 1 OS=Homo sapiens OX=9606 GN=NCKAP1 PE=1 SV=1 |
| Q15025 | 39.67 | 9.79E+05 | 1 | TNFAIP3-interacting protein 1 OS=Homo sapiens OX=9606 GN=TNIP1 PE=1 SV=2 |
| O15511 | 39.64 | 5.60E+04 | 1 | Actin-related protein 2/3 complex subunit 5 OS=Homo sapiens OX=9606 GN=ARPC5 PE=1 SV=3 |
| Q9NRP0 | 37.98 | 1.64E+05 | 1 | Oligosaccharyltransferase complex subunit OSTC OS=Homo sapiens OX=9606 GN=OSTC PE=1 SV=1 |
| Q96IZ0 | 36.59 | 2.88E+05 | 1 | PRKC apoptosis WT1 regulator protein OS=Homo sapiens OX=9606 GN=PAWR PE=1 SV=1 |
| P62333 | 36.32 | 1.79E+05 | 1 | 26S proteasome regulatory subunit 10B OS=Homo sapiens OX=9606 GN=PSMC6 PE=1 SV=1 |
| Q6UN15 | 35.82 | 5.22E+05 | 1 | Pre-mRNA 3'-end-processing factor FIP1 OS=Homo sapiens OX=9606 GN=FIP1L1 PE=1 SV=1 |

| Accession | -10lgP | Area | Peptides | Description |
|---|---|---|---|---|
| Q96HC4 | 35.42 | 1.20E+05 | 1 | PDZ and LIM domain protein 5 OS=Homo sapiens OX=9606 GN=PDLIM5 PE=1 SV=5 |
| P01116 | 34.85 | 2.57E+05 | 1 | GTPase KRas OS=Homo sapiens OX=9606 GN=KRAS PE=1 SV=1 |
| P11310 | 34.72 | 2.80E+05 | 1 | Medium-chain specific acyl-CoA dehydrogenase mitochondrial OS=Homo sapiens OX=9606 GN=ACADM PE=1 SV=1 |
| Q96FS4 | 33.96 | 1.95E+07 | 1 | Signal-induced proliferation-associated protein 1 OS=Homo sapiens OX=9606 GN=SIPA1 PE=1 SV=1 |
| Q9BX46 | 33.65 | 1.78E+06 | 1 | RNA-binding protein 24 OS=Homo sapiens OX=9606 GN=RBM24 PE=1 SV=1 |
| P0DSN7 | 33.32 | | 1 | Probable non-functional immunoglobulinn kappa variable 1D-37 OS=Homo sapiens OX=9606 GN=IGKV1D-37 PE=1 SV=1 |
| P05387 | 33.31 | 5.28E+05 | 1 | 60S acidic ribosomal protein P2 OS=Homo sapiens OX=9606 GN=RPLP2 PE=1 SV=1 |
| P51149 | 33.14 | 8.36E+04 | 1 | Ras-related protein Rab-7a OS=Homo sapiens OX=9606 GN=RAB7A PE=1 SV=1 |
| P36776 | 33.05 | 2.52E+05 | 1 | Lon protease homolog mitochondrial OS=Homo sapiens OX=9606 GN=LONP1 PE=1 SV=2 |
| Q92997 | 33.04 | 1.24E+06 | 1 | Segment polarity protein dishevelled homolog DVL-3 OS=Homo sapiens OX=9606 GN=DVL3 PE=1 SV=2 |
| P17980 | 32.67 | 1.93E+05 | 1 | 26S proteasome regulatory subunit 6A OS=Homo sapiens OX=9606 GN=PSMC3 PE=1 SV=3 |
| P35606 | 31.78 | 3.38E+04 | 1 | Coatomer subunit beta' OS=Homo sapiens OX=9606 GN=COPB2 PE=1 SV=2 |
| O75934 | 31.71 | 1.18E+06 | 1 | Pre-mRNA-splicing factor SPF27 OS=Homo sapiens OX=9606 GN=BCAS2 PE=1 SV=1 |
| P09496 | 31.58 | 2.83E+06 | 1 | Clathrin light chain A OS=Homo sapiens OX=9606 GN=CLTA PE=1 SV=1 |
| Q52LJ0 | 31.2 | 1.08E+05 | 1 | Protein FAM98B OS=Homo sapiens OX=9606 GN=FAM98B PE=1 SV=2 |
| Q969S3 | 30.04 | 8.80E+04 | 1 | Zinc finger protein 622 OS=Homo sapiens OX=9606 GN=ZNF622 PE=1 SV=1 |
| Q86X55 | 29.53 | 2.92E+05 | 1 | Histone-arginine methyltransferase CARM1 OS=Homo sapiens OX=9606 GN=CARM1 PE=1 SV=3 |
| Q02413 | 29.47 | 3.80E+05 | 1 | Desmoglein-1 OS=Homo sapiens OX=9606 GN=DSG1 PE=1 SV=2 |
| P56539 | 29.21 | 3.92E+05 | 1 | Caveolin-3 OS=Homo sapiens OX=9606 GN=CAV3 PE=1 SV=1 |
| Q8WU79 | 28.51 | 7.31E+05 | 1 | Stromal membrane-associated protein 2 OS=Homo sapiens OX=9606 GN=SMAP2 PE=1 SV=1 |

| Accession | -10lgP | Area | Peptides | Description |
|---|---|---|---|---|
| Q9NUQ8 | 27.96 | 1.57E+04 | 1 | ATP-binding cassette sub-family F member 3 OS=Homo sapiens OX=9606 GN=ABCF3 PE=1 SV=2 |
| Q13796 | 27.93 | 2.07E+05 | 1 | Protein Shroom2 OS=Homo sapiens OX=9606 GN=SHROOM2 PE=1 SV=1 |
| P07384 | 27.73 | 6.63E+04 | 1 | Calpain-1 catalytic subunit OS=Homo sapiens OX=9606 GN=CAPN1 PE=1 SV=1 |
| P19387 | 27.66 | 7.28E+04 | 1 | DNA-directed RNA polymerase II subunit RPB3 OS=Homo sapiens OX=9606 GN=POLR2C PE=1 SV=2 |
| O75643 | 26.65 | 3.29E+05 | 1 | U5 small nuclear ribonucleoprotein 200 kDa helicase OS=Homo sapiens OX=9606 GN=SNRNP200 PE=1 SV=2 |
| Q16186 | 26.56 | 1.08E+05 | 1 | Proteasomal ubiquitin receptor ADRM1 OS=Homo sapiens OX=9606 GN=ADRM1 PE=1 SV=2 |
| Q86X29 | 25.42 | 0.00E+00 | 1 | Lipolysis-stimulated lipoprotein receptor OS=Homo sapiens OX=9606 GN=LSR PE=1 SV=4 |
| Q969N2 | 25.24 | 1.11E+05 | 1 | GPI transamidase component PIG-T OS=Homo sapiens OX=9606 GN=PIGT PE=1 SV=1 |
| P62070 | 25.12 | 2.50E+05 | 1 | Ras-related protein R-Ras2 OS=Homo sapiens OX=9606 GN=RRAS2 PE=1 SV=1 |
| Q9NZN4 | 24.76 | 4.69E+05 | 1 | EH domain-containing protein 2 OS=Homo sapiens OX=9606 GN=EHD2 PE=1 SV=2 |
| Q9NP79 | 24.72 | 1.52E+05 | 1 | Vacuolar protein sorting-associated protein VTA1 homolog OS=Homo sapiens OX=9606 GN=VTA1 PE=1 SV=1 |
| Q9NY57 | 24.68 | 9.34E+06 | 1 | Serine/threonine-protein kinase 32B OS=Homo sapiens OX=9606 GN=STK32B PE=2 SV=1 |
| P46087 | 23.81 | 3.93E+05 | 1 | Probable 28S rRNA (cytosine(4447)-C(5))-methyltransferase OS=Homo sapiens OX=9606 GN=NOP2 PE=1 SV=2 |
| Q96KR1 | 23.7 | 3.06E+05 | 1 | Zinc finger RNA-binding protein OS=Homo sapiens OX=9606 GN=ZFR PE=1 SV=2 |
| P20020 | 23.3 | 5.88E+05 | 1 | Plasma membrane calcium-transporting ATPase 1 OS=Homo sapiens OX=9606 GN=ATP2B1 PE=1 SV=4 |
| Q13772 | 22.88 | 2.61E+05 | 1 | Nuclear receptor coactivator 4 OS=Homo sapiens OX=9606 GN=NCOA4 PE=1 SV=1 |
| P11172 | 22.42 | 6.52E+04 | 1 | Uridine 5'-monophosphate synthase OS=Homo sapiens OX=9606 GN=UMPS PE=1 SV=1 |
| O60884 | 22.36 | 2.06E+06 | 1 | DnaJ homolog subfamily A member 2 OS=Homo sapiens OX=9606 GN=DNAJA2 PE=1 SV=1 |
| O95140 | 22.3 | 5.00E+05 | 1 | Mitofusin-2 OS=Homo sapiens OX=9606 GN=MFN2 PE=1 SV=3 |

| Accession | -10lgP | Area | Peptides | Description |
|---|---|---|---|---|
| O76031 | 22.23 | 2.72E+05 | 1 | ATP-dependent Clp protease ATP-binding subunit clpX-like mitochondrial OS=Homo sapiens OX=9606 GN=CLPX PE=1 SV=2 |
| Q92674 | 21.23 | 8.06E+04 | 1 | Centromere protein I OS=Homo sapiens OX=9606 GN=CENPI PE=1 SV=2 |
| P53680 | 21.13 | 1.97E+05 | 1 | AP-2 complex subunit sigma OS=Homo sapiens OX=9606 GN=AP2S1 PE=1 SV=2 |
| O15031 | 21.04 | 1.23E+05 | 1 | Plexin-B2 OS=Homo sapiens OX=9606 GN=PLXNB2 PE=1 SV=3 |
| P06312 | 20.75 | 3.66E+05 | 1 | Immunoglobulin kappa variable 4-1 OS=Homo sapiens OX=9606 GN=IGKV4-1 PE=1 SV=1 |
| Q9P0L0 | 20.63 | 6.77E+05 | 1 | Vesicle-associated membrane protein-associated protein A OS=Homo sapiens OX=9606 GN=VAPA PE=1 SV=3 |
| Q99442 | 20.57 | 0.00E+00 | 1 | Translocation protein SEC62 OS=Homo sapiens OX=9606 GN=SEC62 PE=1 SV=1 |
| Q96NG3 | 20.2 | 8.50E+05 | 1 | Outer dynein arm-docking complex subunit 4 OS=Homo sapiens OX=9606 GN=ODAD4 PE=1 SV=2 |

**Table S4. The contact list between TRIM21, TRIM29 and USP18 in the trimeric complex.**

| Chain 1 | Residue 1 | Chain 2 | Residue 2 | Interaction type |
|---|---|---|---|---|
| TRIM21 | Glu304 | TRIM29 | Met548 | Hydrogen bond |
| TRIM21 | Arg310 | TRIM29 | Gln499 | Hydrogen bond |
| TRIM21 | Ser317 | TRIM29 | Asn525 | Hydrogen bond |
| TRIM21 | Thr457 | TRIM29 | Lys478 | Hydrogen bond |
| TRIM21 | Asp326 | USP18 | Asn353 | Hydrogen bond |
| TRIM21 | Asp326 | USP18 | Asn355 | Hydrogen bond |
| TRIM21 | Ser327 | USP18 | Tyr356 | Hydrogen bond |
| TRIM21 | Trp299 | USP18 | Gly352 | Hydrogen bond |
| TRIM21 | Asp313 | USP18 | Tyr274 | Hydrogen bond |
| TRIM21 | Thr314 | USP18 | Ser272 | Hydrogen bond |
| TRIM21 | Gln315 | USP18 | Ser272 | Hydrogen bond |
| TRIM21 | Gln315 | USP18 | Asn353 | Hydrogen bond |
| TRIM21 | Gln315 | USP18 | Pro354 | Hydrogen bond |
| USP18 | Asp200 | TRIM29 | Lys554 | Salt bridge |
| USP18 | Lys204 | TRIM29 | Asn444 | Hydrogen bond |
| USP18 | Lys207 | TRIM29 | Ser552 | Hydrogen bond |
| USP18 | Asp211 | TRIM29 | Ala555 | Hydrogen bond |
| USP18 | Cys215 | TRIM29 | Glu443 | Hydrogen bond |
| USP18 | Asp280 | TRIM29 | Met427 | Hydrogen bond |
| USP18 | Ser282 | TRIM29 | Lys426 | Hydrogen bond |
| USP18 | Ser282 | TRIM29 | Cys428 | Hydrogen bond |
| USP18 | Gln283 | TRIM29 | Cys428 | Hydrogen bond |
| USP18 | Gln283 | TRIM29 | Met468 | Hydrogen bond |

**Table S5. The binding free energy between TRIM29 dimer model with ubiquitin or without ubiquitin.**

| Contribution* (unit: kcal/mol) | Model without ubiquitin | Model with ubiquitin |
|---|---|---|
| $\Delta E_{vdw}$ | -374.42 ± 15.20 | -432.93 ± 15.30 |
| $\Delta E_{ele}$ | -809.95 ± 69.90 | -853.78 ± 102.28 |
| $\Delta G_{polar}$ | 943.33 ± 69.51 | 1011.71 ± 102.65 |
| $\Delta G_{nonpolar}$ | -31.88 ± 5.78 | -43.59 ± 6.64 |
| $\Delta G_{total}$ | -209.16 ± 19.81 | -331.41 ± 18.58 |

* The contribution to the binding free energy (ΔGtotal) from the Van der Waals and electrostatic interactions is represented by ΔEvdw and ΔEele, respectively. The polar and nonpolar solvation energy contributions to ΔGtotal are represented by ΔGpolar and ΔGnonpolar, respectively.

† The binding free energy was obtained from the MM/PBSA calculation.

**Table S6. Correlations between USP18 expression and the clinical characteristics of patients with locoregionally advanced NPC.**

| Characteristic | Low expression group (%) n = 107 | High expression group (%) n = 93 | $p$-value* |
|---|---|---|---|
| Age | | | |
| ≤45 | 53 (49.5%) | 52 (55.9%) | 0.367 |
| ＞45 | 54 (50.5%) | 41 (44.1%) | |
| Sex | | | |
| Male | 76 (71.0%) | 73 (78.5%) | 0.227 |
| Female | 31 (29.0%) | 20 (21.5%) | |
| TNM stage† | | | |
| I/II | 43 (40.2%) | 28 (30.1%) | 0.137 |
| III/IV | 64 (59.8%) | 65 (69.9%) | |
| Locoregional recurrence | | | 0.004 |
| Yes | 8 (7.5%) | 20 (21.5%) | |
| No | 99 (92.5%) | 73 (78.5%) | |
| Disease | | | |
| Yes | 18 (16.8%) | 42 (45.2%) | ＜0.001 |
| No | 89 (83.2%) | 51 (54.8%) | |
| Death | | | |
| Yes | 11 (10.3%) | 30 (32.3%) | ＜0.001 |
| No | 96 (89.7%) | 63 (67.7%) | |

* Two-sided Chi-square test. All patients were restaged according to the AJCC Cancer Staging Manual, 8$^{th}$ edition.

**Table S7. Univariate and multivariate Cox regression analysis of prognostic factors according to different end points.**

Overall survival

| Variation | Univariate analysis | | Multivariate analysis | |
|---|---|---|---|---|
| | *p* | HR (95% CI) | *p* | HR (95% CI) |
| USP18 level (low vs high) | ＜0.001 | 3.916 (1.954-7.846) | 0.001 | 3.483 (1.719-7.059) |
| TNM stage (III/IV vs I/II) | 0.004 | 1.870 (1.215-2.876) | 0.021 | 1.646 (1.077-2.514) |
| Age (≤45y vs ＞45y) | 0.407 | 1.304 (0.696-2.444) | 0.278 | 1.422 (0.753-2.684) |
| Sex（male vs female） | 0.089 | 0.471 (0.198-1.122) | 0.224 | 0.58 (0.241-1.395) |

Disease-free survival

| Variation | Univariate analysis | | Multivariate analysis | |
|---|---|---|---|---|
| | *p* | HR (95% CI) | *p* | HR (95% CI) |
| USP18 level (low vs high) | ＜0.001 | 3.685 (2.116-6.417) | ＜0.001 | 3.180 (1.799-5.621) |
| TNM stage (III/IV vs I/II) | 0.001 | 1.726 (1.239-2.406) | 0.007 | 1.561 (1.128-2.160) |
| Age (≤45y vs ＞45y) | 0.903 | 1.032 (0.621-1.715) | 0.492 | 1.203 (0.710-2.040) |
| Sex（male vs female） | 0.022 | 0.438 (0.216-0.890) | 0.088 | 0.536 (0.262-1.098) |

Locoregional survival

| Variation | Univariate analysis | | Multivariate analysis | |
|---|---|---|---|---|
| | *p* | HR (95% CI) | *p* | HR (95% CI) |
| USP18 level (low vs high) | 0.002 | 3.752 (1.647-8.547) | 0.003 | 3.575 (1.539-8.304) |
| TNM stage (III/IV vs I/II) | 0.553 | 1.144 (0.733-1.785) | 0.755 | 1.072 (0.693-1.658) |
| Age (≤45y vs ＞45y) | 0.909 | 1.044 (0.497-2.195) | 0.664 | 1.188 (0.547-2.576) |
| Sex（male vs female） | 0.785 | 0.888 (0.377-2.089) | 0.899 | 1.058 (0.441-2.542) |

**Table S8. List of primers used in this study.**

| Gene | Sequence (forward: 5′→3′) |
|---|---|
| sgRNA and shRNA | |
| USP18-sg1 | CCTGTATACGATCCGGGTGA |
| USP18-sg2 | AGGGCACGTTGCACTTCTGC |
| shTRIM29-1 | GCCACGTTGAGAAGATGTGCAA |
| shTRIM29-2 | CGGACCTGAGCCGTAACTTCATTG |
| siRNA | |
| USP18-si1 | CCAGGGAGTTATCAAGCAA |
| USP18-si2 | CATCCGGAATGCTGTGGAT |
| RT-qPCR | |
| USP18-F | TGGACAGACCTGCTGCCTTAAC |
| USP18-R | TGGACAGACCTGCTGCCTTAAC |
| TRIM29-F | GAGGATGAAGCTGAGAAGTGGC |
| TRIM29-R | CCTCCTTTTGCTTCTCCAGGTC |
| TRIM21-F | CAGAACTCAGGAGTGTGTGCCA |
| TRIM21-R | TCCAAGCCTCACTTGTCTCCGA |
| GAPDH-F | GTCTCCTCTGACTTCAACAGCG |
| GAPDH-R | ACCACCCTGTTGCTGTAGCCAA |
| Actin-F | TCACCAACTGGGACGACATG |
| Actin-R | GTCACCGGAGTCCATCCGAT |
| IFIT1-F | GCCTTGCTGAAGTGTGGAGGAA |
| IFIT1-R | ATCCAGGCGATAGGCAGAGATC |
| IFIT2-F | GGAGCAGATTCTGAGGCTTTGC |
| IFIT2-R | GGATGAGGCTTCCAGACTCCAA |
| MX1-F | GGCTGTTTACCAGACTCCGACA |
| MX1-R | CACAAAGCCTGGCAGCTCTCTA |
| ISG15-F | CTCTGAGCATCCTGGTGAGGAA |
| ISG15-R | AAGGTCAGCCAGAACAGGTCGT |
| OAS1-F | AGGAAAGGTGCTTCCGAGGTAG |
| OAS1-R | GGACTGAGGAAGACAACCAGGT |
| CXCL10-F | GGTGAGAAGAGATGTCTGAATCC |
| CXCL10-R | GTCCATCCTTGGAAGCACTGCA |

**Table S9. List of antibodies used in this study.**

| Antibodies | Source | Catalogue No. |
|---|---|---|
| For Western blot | | |
| anti-GAPDH | Proteintech | 60004-1-Ig |
| anti-α-Tubulin | Proteintech | 66031-1-Ig |
| anti-Flag | Sigma | F1804 |
| anti-HA | Sigma | H6908 |
| anti-Myc | Cell Signaling Technology | 2278S |
| anti-USP18 | Cell Signaling Technology | 4813S |
| anti-TRIM21 | Proteintech | 12108-1-AP |
| anti-TRIM29 | Proteintech | 17542-1-AP |
| anti-Fibrillarin | Proteintech | 16021-1-AP |
| anti-H3 | Proteintech | 17168-1-AP |
| Anti-mouse IgG, HRP-linked | Cell Signaling Technology | 7076S |
| Anti-mouse IgG, HRP-linked | Cell Signaling Technology | 7074S |
| Co-Immunoprecipitation | | |
| anti-Flag | Sigma | F1804 |
| anti-Myc | Cell Signaling Technology | 2278S |
| anti-USP18 | Cell Signaling Technology | 4813S |
| anti-TRIM21 | Proteintech | 12108-1-AP |
| Rabbit IgG control | Proteintech | 30000-0-AP |
| Mouse IgG | Proteintech | B900620 |
| Immunofluorescence | | |
| anti-Myc | Cell Signaling Technology | 2278S |
| anti-Flag | Sigma | F1804 |
| anti-TRIM29 | Proteintech | 17542-1-AP |
| Phospho-Histone H2A.X (Ser139) (D7T2V) | Cell Signaling Technology | 80312S |
| anti-ISG15 | Proteintech | 15981-1-AP |
| For Immunohistochemistry | | |
| anti-USP18 | Abmart | PHZ9559M |
